# Synthesis, Physicochemical Characterization, and Biocidal Evaluation of Three Novel Aminobenzoic Acid-Derived Schiff Bases Featuring Intramolecular Hydrogen Bonding

**DOI:** 10.3390/ijms262110801

**Published:** 2025-11-06

**Authors:** Alexander Carreño, Vania Artigas, Belén Gómez-Arteaga, Evys Ancede-Gallardo, Marjorie Cepeda-Plaza, Jorge I. Martínez-Araya, Roxana Arce, Manuel Gacitúa, Camila Videla, Marcelo Preite, María Carolina Otero, Catalina Guerra, Rubén Polanco, Ignacio Fuentes, Pedro Marchant, Osvaldo Inostroza, Fernando Gil, Juan A. Fuentes

**Affiliations:** 1 Departamento de Ciencias Químicas, Facultad de Ciencias Exactas, Universidad Andres Bello, Av. República 275, Santiago 8370146, Chile; marjorie.cepeda@unab.cl (M.C.-P.); jorge.martinez@unab.cl (J.I.M.-A.); roxana.arce@unab.cl (R.A.); 2 Laboratorio de Síntesis Organometálica, Centro de Nanociencias Aplicadas (CANS), Facultad de Ciencias Exactas, Universidad Andres Bello, Av. República 330, Santiago 8370186, Chile; vania.artigas@pucv.cl (V.A.); belen.gomez@usach.cl (B.G.-A.); eancedeg@gmail.com (E.A.-G.); camila.videla.e@gmail.com (C.V.); 3 Millennium Institute on Green Ammonia as Energy Vector (MIGA), Av. Vicuña Mackenna 4860, Macul, Santiago 7820436, Chile; 4 Facultad de Ingeniería y Ciencias, Universidad Diego Portales, Ejército 441, Santiago 8370191, Chile; manuel.gacitua@mail.udp.cl; 5 Escuela de Química y Farmacia, Facultad de Medicina, Universidad Andres Bello, Sazié 2320, Santiago 7591538, Chile; maria.otero@unab.cl; 6 Departamento de Química Orgánica, Facultad de Química y de Farmacia, Pontificia Universidad Católica de Chile, Av. Vicuña Mackenna 4860, Santiago 7820436, Chile; mpreite@uc.cl; 7 Laboratorio de Hongos Fitopatógenos, Centro de Biotecnología Vegetal (CBV), Facultad de Ciencias de la Vida, Universidad Andres Bello, Av. República 330, Santiago 8370186, Chile; c.guerraramrez@uandresbello.edu (C.G.); rpolanco@unab.cl (R.P.); 8 Laboratorio de Genética y Patogénesis Bacteriana, Centro de Investigación de Resiliencia a Pandemias, Facultad de Ciencias de la Vida, Universidad Andres Bello, República 330, Santiago 8370186, Chile; ignaciofuentesc547@gmail.com (I.F.); marchant573@gmail.com (P.M.); 9 Doctorado en Biotecnología, Facultad de Ciencias de la Vida, Universidad Andrés Bello, República 330, Santiago 8370186, Chile; 10 School of Medicine, Faculty of Medicine, Universidad de los Andes, Santiago 7620001, Chile; osv.inostroza.t@gmail.com (O.I.); frgil@uandes.cl (F.G.); 11 Microbiota-Host Interactions & Clostridia Research Group, Center for Biomedical Research and Innovation (CIIB), Universidad de los Andes, Av. Monseñor Álvaro del Portillo 12455, Santiago 7620001, Chile

**Keywords:** Schiff bases, aminobenzoic acid, intramolecular hydrogen bonding, biocidal activity, hela cell viability, cyclic voltammetry, X-ray crystallography, MEP, LHS

## Abstract

Metal-free aminobenzoic acid-derived Schiff bases are attractive antimicrobial leads because their azomethine (–C=N–) functionality enables tunable electronic properties and target engagement. We investigated whether halogenation on the phenolic ring would modulate the redox behavior and enhance antibacterial potency, and hypothesized that heavier halogens would favorably tune physicochemical and electronic descriptors. We synthesized three derivatives (SB-3/Cl, SB-4/Br, and SB-5/I) and confirmed their structures using FTIR, ^1^H- and ^13^C-NMR, UV-Vis, and HRMS. For SB-5, single-crystal X-ray diffraction and Hirshfeld analysis verified the intramolecular O–H⋯N hydrogen bond and key packing contacts. Cyclic voltammetry revealed an irreversible oxidation (aminobenzoic ring) and, for the halogenated series, a reversible reduction associated with the imine; peak positions and reversibility trends are consistent with halogen electronic effects and DFT-based MEP/LHS descriptors. Antimicrobial testing showed that SB-5 was selectively potent against Gram-positive aerobes, with low-to-mid micromolar MICs across the panel. Among anaerobes, activity was more substantial: *Clostridioides difficile* was inhibited at 0.1 µM, and SB-3/SB-5 reduced its sporulation at sub-MICs, while *Blautia coccoides* was highly susceptible (MIC 0.01 µM). No activity was detected against Gram-negative bacteria at the tested concentrations. In the fungal assay, *Botrytis cinerea* displayed only a transient fungistatic response without complete growth inhibition. In mammalian cells (HeLa), the compounds displayed clear concentration-dependent behavior. Overall, halogenation, particularly iodination, emerges as a powerful tool to couple redox tuning with selective Gram-positive activity and a favorable cellular tolerance window, nominating SB-5 as a promising scaffold for further antimicrobial optimization.

## 1. Introduction

Schiff bases are imines formed by condensing primary amines with aldehydes or ketones and are characterized by the azomethine group (–C=N–) [1,2,3]. Their structural versatility permits extensive substitution (aryl, alkyl, heteroaryl, cycloalkyl), underpinning broad utility in catalysis, dyes/pigments, polymer stabilization, and organic synthesis, alongside notable antibacterial, antifungal, antiviral, anti-inflammatory, and antioxidant activities [1,2,3,4,5,6,7,8,9]. Depending on the metal coordination, they are broadly categorized as metal-coordinated or metal-free. Coordination complexes (e.g., with Cu, Zn, Fe) may display enhanced stability and bioactivity, whereas metal-free systems offer synthetic simplicity and remain widely used in biochemical research [10,11,12,13,14,15,16].

The antimicrobial activity of aminobenzoic acid–derived Schiff bases (metal-free) is often linked to the azomethine group, which can form hydrogen bonds at microbial enzyme sites and disrupt essential pathways [3,6,17,18,19,20,21]. Rational substitution enables electronic and steric tuning that strengthens such interactions, improves molecular stability, and modulates solubility in biological media [3,17,18,19]. Within this framework, halogenation is a particularly effective strategy, as the type and position of halogens significantly influence electronic distribution and redox behavior, while steric bulk and lipophilicity impact membrane permeation and target engagement [17,18,19,22]. Although prior studies have focused on halogenated phenolic scaffolds, related principles can be leveraged in distinct Schiff base frameworks to optimize antimicrobial performance [17,18,19].

Guided by these considerations, we hypothesized that tailored halogen substitution would modulate electronic properties and redox profiles of aminobenzoic acid–derived Schiff bases (metal-free) and, in turn, their biocidal activity. This study aimed to synthesize and validate three aminobenzoic acid-derived Schiff bases bearing intramolecular hydrogen bonding, and to elucidate how halogen substitution modulates their physicochemical/redox properties (via cyclic voltammetry and MEP/LHS descriptors) and their biocidal activity against bacteria and fungi, alongside mammalian cytotoxicity. We therefore synthesized three novel derivatives (SB-3, SB-4, SB-5) featuring intramolecular hydrogen bonding (IHB) that stabilizes a six-membered pseudo-aromatic ring, as established by spectroscopic characterization (FTIR, NMR, UV-Vis) and high-resolution mass spectrometry [23,24]. Electrochemical analysis by cyclic voltammetry revealed halogen-dependent redox modulation, which we interpreted in conjunction with electronic structure descriptors (molecular electrostatic potential [MEP] and local hardness/softness [LHS]) to rationalize reactivity trends [25,26,27]. Finally, we evaluated the antibacterial activity against both Gram-positive and Gram-negative strains, the antifungal effects, and the cytotoxicity to HeLa cells to delineate the structure-bioactivity relationships. Collectively, our results show that halogenation significantly influences the physicochemical and biocidal profiles of aminobenzoic Schiff bases, and identify the iodine-substituted SB-5 as a promising candidate for further antimicrobial development.

## 2. Results and Discussion

### 2.1. Synthesis and Characterization of Schiff Bases

**Figure 1:**
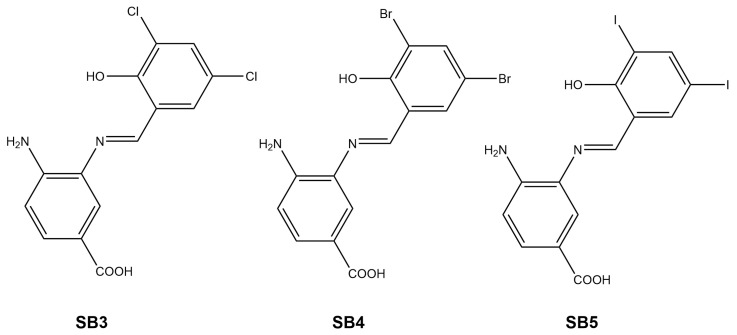
Chemical structures of (*E*)-4-amino-3-((3,5-di-chloride-2-hydroxybenzylidene)amino) benzoic acid (SB-3), (*E*)-4-amino-3-((3,5-di-bromide-2-hydroxybenzylidene)amino) benzoic acid (SB-4) and (*E*)-4-amino-3-((3,5-di-iodide-2-hydroxybenzylidene)amino) benzoic acid (SB-5).

SB-3, SB-4, and SB-5 (Figure 1) are aminobenzoic acid derivatives bearing a phenolic ring linked via an azomethine group and stabilized by an intramolecular hydrogen bond (IHB) between the phenolic –OH and the Schiff base nitrogen (–N=C–). As previously described, each compound was synthesized by directly condensing a primary amine with the corresponding substituted aldehyde in methanol at room temperature (Scheme S1) [17,18]. All three Schiff bases were synthesized in high yields (see Section 3.2.1, Section 3.2.2 and Section 3.2.3) and exhibited a characteristic light orange color.

These derivatives are highly soluble in dimethyl sulfoxide (DMSO) and dimethylformamide (DMF), less soluble in methanol and acetonitrile, and essentially insoluble in water at ambient temperature. Analysis of their melting points revealed that SB-5, which carries two iodine atoms, has the highest melting point, followed by SB-4 (two bromine atoms) and SB-3 (two chlorine atoms). These trends are consistent with the nature of the substituents and support the proposed structures. Further details on reaction yields and other constants for these newly synthesized aminobenzoic acid-derived Schiff Bases are provided in Table S1.

High-resolution electrospray mass spectrometry (HRMS) confirmed the molecular assignments, showing ions at *m*/*z* 324.9973 for SB-3, 412.9035 for SB-4, and 508.8863 for SB-5 (Figures S1–S3). The spectral and isotopic analyses of SB3, SB4, and SB5 strongly support their proposed compositions. For SB3, the HRMS spectrum displays a characteristic isotopic pattern with peaks at [M]^+^, [M + 2]^+^, and [M + 4]^+^, consistent with the presence of two chlorine atoms and their natural isotopic distribution (^35^Cl and ^37^Cl), showing relative intensities of approximately 100:60:10 [28,29,30] (Figure S1). Similarly, SB4 exhibits isotopic multiplicity due to ^79^Br and ^81^Br, confirming the presence of bromine atoms [31] (Figure S2). In the case of SB5, the HRMS spectrum shows a single molecular ion peak with a distinctive isotopic signature arising from two iodine atoms, predominantly iodine-127 [32] (Figure S3).

**Figure 2:**
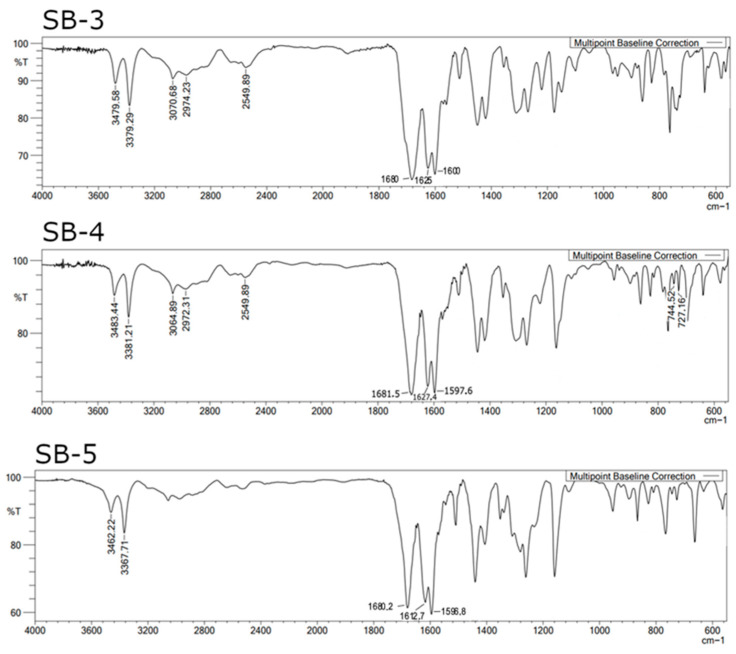
FTIR spectra of SB-3, SB-4, and SB-5.

**Figure 3:**
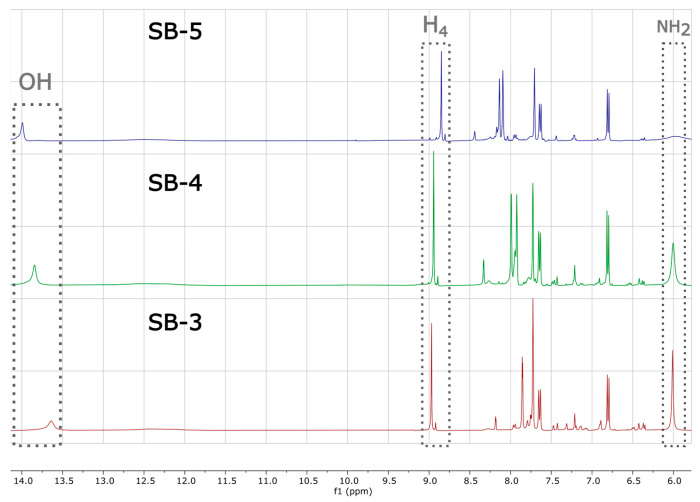
^1^H-NMR spectra of SB-3, SB-4, and SB-5 recorded in deuterated DMSO.

The FTIR spectra of SB-3, SB-4, and SB-5 (KBr pellet) showed broad absorption bands between 2500 and 4000 cm^−1^ (See Figure 2), assigned to a νOH band, two distinctive, strong, and sharp symmetric and asymmetric νNH_2_ bands, and strong absorption near 1680 cm^−1^ typical of a carboxylic acid group. Vibrations at 1625–1600 cm^−1^ were consistent with the azomethine (–C=N–) stretching [33,34], while signals around 1600 cm^−1^ correspond to aromatic –C=C– stretching, in line with previous reports for related Schiff bases [35,36]. The shifting of the azomethine (νC=N) band and the νOH bands are further indicators of the presence of an intramolecular hydrogen bond (IHB) [37,38]. For more details, see Figures S4–S6. To confirm the proposed structures, we performed 1D and 2D NMR studies, including ^1^H- and ^13^C-NMR spectra in deuterated DMSO, with a D_2_O exchange, DEPT-45, and HHCOSY experiments. The ^1^H-NMR spectrum was carried out in DMSO-_d6_ as the solvent (see Figure S7 for the arbitrary proton numbering of SB compounds). The peaks observed around 2.50 and 3.33 ppm are assigned to the DMSO-_d6_ residual peak and water signals in all the ^1^H-NMR spectra reported here [39]. As expected, SB-3, SB-4, and SB-5 exhibited two narrow signals around 5.99 ppm and 13.82 ppm (Figure 3), assigned to the amino group (–NH_2_) protons and the hydroxyl group (–OH), respectively (For more details, see Figures S8–S10; and the expanded aromatic region is shown in Figures S11–S13). The narrow, low-intensity signal around 13.0 ppm indicates intramolecular hydrogen bonding (IHB), confirmed by SB-5 X-ray diffraction (see below). On the other hand, a less intense, broader signal appeared around 12.32 ppm, assigned to the carboxylic group (–COOH) (Table S2). All these assignments were confirmed by D_2_O exchange (Figures S14–S16; compare with Figures S8–S10). In the case of the azomethine proton (H4), it was observed as a singlet at around 8.93 ppm. The other aromatic protons corresponding to the amino-benzoic acid ring were observed around 6.80 ppm (d, H2) and 7.68 ppm (s, H6). Regarding H1, it appeared as a multiplet that was partially overlapped with H5 at approximately 7.66–7.52 ppm. Finally, H6 (found in the phenol ring) appeared at 7.38 ppm (d). All these assignments were confirmed by HHCOSY (Figures S17–S19). These experiments confirmed our assignment of all signals to their respective protons.

To further confirm the structural characterization, the ^13^C-NMR spectra of these compounds were obtained (Figures S20–S22). Fourteen distinct carbon signals were noted in the 170–110 ppm range, consistent with the presence of the aromatic and carboxylic acid moieties. Notably, signals attributed to the carboxyl (–COOH) and azomethine (–N=C–) carbons [40,41] were observed at d 167 ppm and 161 ppm, respectively, in agreement with previous reports for related Schiff bases [3]. Table S3 provides a summary of the key assignments.

Additionally, the phenolic carbon atom bonded to the –OH substituent appeared at 156.36 ppm in both SB-3 and SB-4 and at 160.04 ppm in SB-5. To distinguish quaternary carbons from tertiary carbons, a DEPT-45 experiment was performed, revealing six tertiary carbons and thereby confirming the structures of all three compounds (Figures S23–S25).

Combined analyses indicated that, in the ^1^H NMR spectra, the halogen induces a downfield shift in the –OH proton when changing from chlorine to iodine, whereas the azomethine proton (H4) exhibits an upfield shift (Table S2). In the ^13^C NMR spectra, a downfield shift in the azomethine carbon was observed upon substitution with iodine relative to chlorine. This effect may be explained by an intramolecular hydrogen bond (IHB) that forms a six-membered pseudo-aromatic ring involving the azomethine nitrogen and the phenolic ring’s hydrogen, modulated by the specific halogen substituent [24,42,43]. The increased electron-withdrawing capacity at position 3 of the phenolic ring also enhances the inductive effect [44]. Additionally, the presence of halogen atoms can establish a halogen–carbon dipole that favors the interaction with the neighboring –OH group in the phenolic ring [45]. Consequently, the formation of an IHB becomes progressively more favorable from chlorine to iodine.

### 2.2. UV-Vis Studies

The UV-Vis absorption spectra of SB-3, SB-4, and SB-5 were measured in aerated methanol (ε = 32.6) and DMSO (ε = 46.7) at room temperature. The resulting spectroscopic data are summarized in Table 1. The spectra of the three compounds in DMSO and MeOH (Figures S26–S28) show two distinct absorption bands for each compound in both solvents. The first, narrower band appears near 280 nm in methanol and 288 nm in DMSO, corresponding to combined n → π * transitions of the C=O and –C=N– groups, as well as π → π * transitions within the aromatic rings [46]. A second, broader band is centered at 393 nm in methanol and 408 nm in DMSO, showing a bathochromic shift with increasing solvent polarity (Table 1). This broader band undergoes a slightly more pronounced shift to higher energies than the narrower band under more polar conditions, supporting its assignment as a π → π * transition influenced by intramolecular charge transfer. In particular, the azomethine group (–C=N–), which forms an intramolecular hydrogen bond (IHB), appears to lower the π → π * transition energy, which is consistent with observations reported for structurally related compounds [47,48,49,50]. SB-3 exhibits relatively stable interactions in both methanol and DMSO, whereas SB-4 and SB-5 show stronger affinity for DMSO compared to methanol. These findings are consistent with the electronic nature of the Schiff base derivatives, in which increasing halogen size and polarizability (from chlorine to iodine) substantially affect their interactions with solvent polarity. The intramolecular hydrogen bond (IHB), previously confirmed by FTIR, ^1^H-NMR, and D_2_O exchange, further supports these observations. Beyond the structural and photophysical characterization, the luminescent properties of the SB series were also explored to gain a deeper understanding of their electronic behavior. Emission spectra of SB-3 and SB-4 were recorded in DMSO and MeOH (Figure S28), revealing a distinct emission band centered around 450 nm for both compounds. SB-4 exhibited a noticeable red shift relative to SB-3 in both solvents. In contrast, SB-5 displayed significantly weaker fluorescence and a noisy emission profile. Unlike the other derivatives, its emission was too weak and dominated by noise, which hindered reliable spectral characterization.

On the other hand, the emission intensity across the SB series decreased progressively from the dichlorinated to the diiodinated phenolic-substituted Schiff base, in agreement with the enhanced spin–orbit coupling effect associated with iodine atoms. This behavior is reflected in the quantum yield values: SB-3 exhibited 0.267 and 0.101 in DMSO and MeOH, respectively, while SB-4 showed markedly lower values of 0.018 and 0.016 in the same solvents. These findings underscore the impact of heavy-atom substitution within the phenolic moiety on the compounds’ luminescent efficiency [51,52].

### 2.3. X-Ray Structure of SB-5

Among the synthesized compounds, SB-5 was the only one for which a crystalline structure was successfully obtained. Spectroscopic analyses were performed on SB-5 and compared with data from complementary techniques, including FTIR and NMR. This comprehensive approach provides structural validation for SB-5 and serves as a reference for characterizing SB-3 and SB-4, which could not be crystallized.

**Figure 4:**
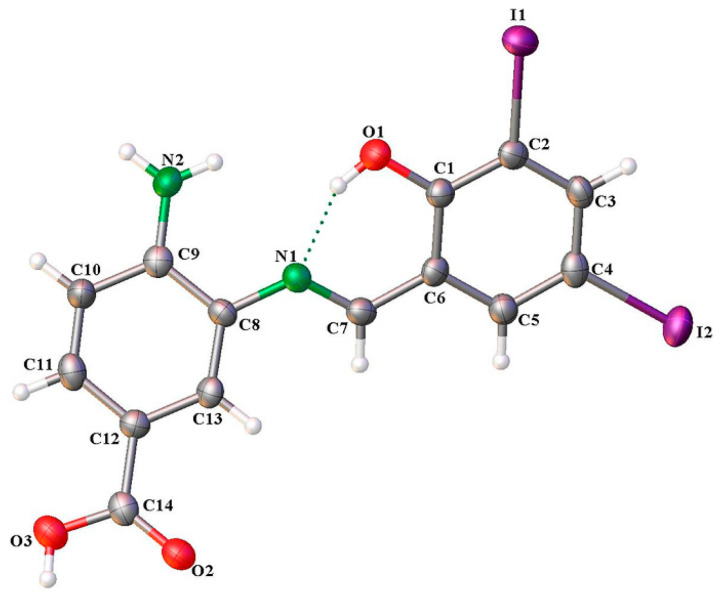
ORTEP drawings of compound SB-5. Thermal ellipsoids are drawn at 50% probability.

ORTEP representations of SB-5, along with its atom numbering scheme, are depicted in Figure 4. The compound crystallized in the monoclinic P2_1_/c space group, with a single molecular entity in the asymmetric unit. Selected bond distances and bond angles are detailed in Table 2.

Table 3 summarizes the intramolecular and intermolecular interactions that confirm the presence of intramolecular hydrogen bonding (IHB) between the R–O–H group and the imino nitrogen, as indicated by the FTIR analysis. The O(1)–H⋯N(1) interaction forms a six-membered pseudo-aromatic ring, stabilizing the resonant fragment ⋯HO=C–C=C–N⋯. Additionally, the structural framework [O(1)–C(1)–C(6)–C(7)–N(1)] exhibits bond angles of 120.9° for C(1)–C(6)–C(7) and 121.2° for C(6)–C(7)–N(1) (Table S4), values that are close to the ideal 120°, suggesting that these atoms adopt sp^2^ hybridization. Furthermore, the bond length analysis of C(1)–O(1), C(1)–C(6), C(6)–C(7), and C(7)–N(1) (Table S4) in SB-5 supports the presence of a conjugated π-system, consistent with the structural arrangement [O(1)=C(1)–C(6)=C(7)–N(1)] [50,53,54].

The formation of the six-membered pseudo-aromatic ring is further supported by the downfield shift in the –OH signal at 13.99 ppm in the ^1^H-NMR spectrum, as well as by the characteristic carbon signal of the azomethine group observed in the ^13^C-NMR spectrum. Table 3 summarizes both intramolecular and intermolecular interactions in the SB-5 structure. Intramolecular interactions include hydrogen bonding between the oxygen of the 3,5-diiodophenol fragment and the imino nitrogen (N1), reinforcing the structural stability of the Schiff base.

**Figure 5:**
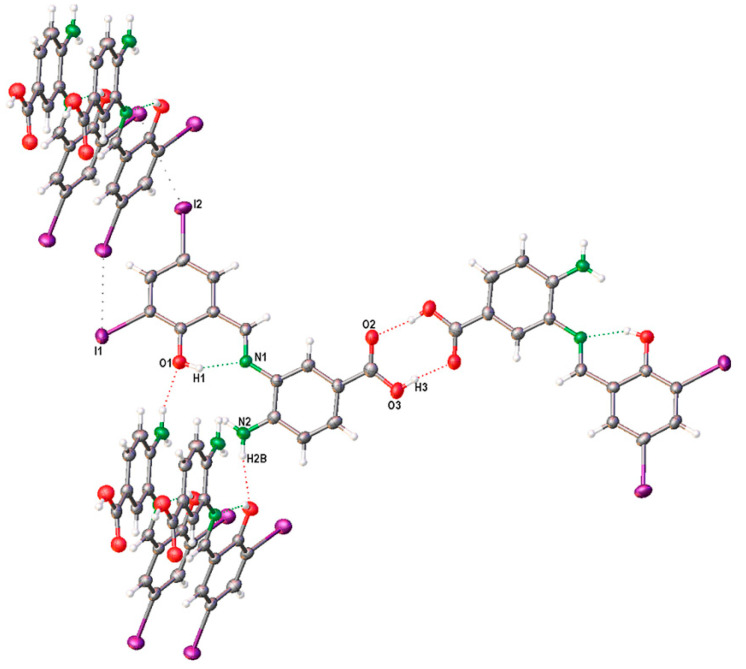
Molecular packing properties of the SB-5 compound.

Conversely, the intermolecular interactions responsible for the crystalline packing of SB-5 indicate that crystal stabilization is achieved through intermolecular hydrogen bonds. Specifically, N(2)–H(2B)⋯O(1) interactions occur between the amine group of the 3,4-diaminobenzoic acid fragment and the oxygen of the 3,5-diiodophenol fragment. Additionally, O(3)–H(3)⋯O(2) hydrogen bonds are observed between carboxyl groups of the 3,4-diaminobenzoic acid fragment. These interactions are structurally correlated by a two-fold screw axis parallel to the *b*-axis and an inversion symmetry centered at the origin, respectively. Furthermore, the crystal packing is further stabilized by intermolecular halogen interactions (I(1)⋯I(2)), which are related through a *b*-glide plane perpendicular to the *a*-axis (Figure 4 and Figure 5).

Finally, the structural analysis revealed that the plane defined by the [OCCCN] framework (plane 1) forms a dihedral angle with the substituted phenyl ring of the 3,4-diaminobenzoic acid fragment (plane 2). The magnitude of this dihedral angle suggests that these two planes are not coplanar, indicating a lack of extended conjugation between them (Table S4).

Further evidence supporting the formation of the six-membered pseudo-aromatic ring is provided by the downfield shift in the –OH signal at 13.99 ppm in the ^1^H-NMR spectrum, as well as by the characteristic carbon resonance of the azomethine group in the ^13^C NMR spectrum. Table 3 summarizes both intramolecular and intermolecular interactions in the SB-5 structure. Intramolecular stabilization is primarily mediated by hydrogen bonding between the oxygen of the 3,5-diiodophenol fragment and the imino nitrogen (N1).

Conversely, intermolecular interactions involved in the crystalline packing of SB-5 indicate that the crystal structure is stabilized through hydrogen bonding. Specifically, N(2)–H(2B)⋯O(1) interactions occur between the amine group of the 3,4-diaminobenzoic acid fragment and the oxygen of the 3,5-diiodophenol fragment, while O(3)–H(3)⋯O(2) interactions are observed between carboxyl groups within the 3,4-diaminobenzoic acid moiety. These interactions are structurally correlated by a two-fold screw axis parallel to the *b*-axis and an inversion center at the origin, respectively.

**Figure 6:**
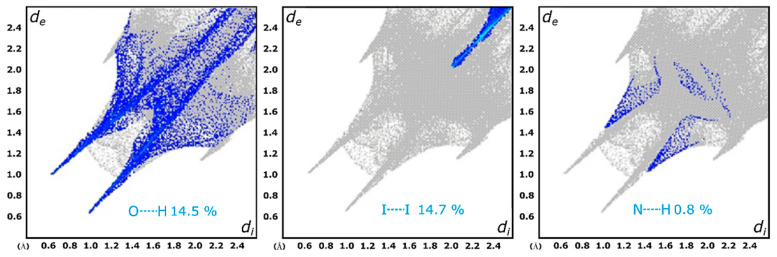
Hirshfeld-surface–derived 2D fingerprint plots for SB-5 highlighting the principal intermolecular contact contributions. The gray background shows the full fingerprint (all contacts on the Hirshfeld surface of SB-5; *d*i, Å, is the distance from the surface to the nearest atom inside; *d*e, Å, to the nearest atom outside). Blue points are the decomposed fingerprints for the specified contact in each panel—O⋯H/H⋯O, I⋯I, or N⋯H/H⋯N—i.e., only contacts of that type overlaid on the gray background. The blue percentage in each panel denotes that interaction’s contribution to the Hirshfeld surface area.

Additionally, crystal packing is further stabilized by intermolecular halogen interactions (I(1)⋯I(2)), which are related through a *b*-glide plane perpendicular to the *a*-axis (Figure 5). These structural features collectively contribute to the stability and organization of the SB-5 crystalline lattice. The intermolecular interactions within the crystal structure of SB-5 were quantified using Hirshfeld surface [55,56] analysis and fingerprint plots, as illustrated in Figure 6. The mapped surfaces, analyzed with respect to *d_norm_*, highlight key interaction regions. The most significant contributions of different intermolecular interactions in SB-5 are summarized in Figure 5. Fingerprint plot analysis reveals that the predominant interactions in the crystal packing of SB-5 are O⋯H and I⋯I contacts, accounting for 14.5% and 14.7% of the total interactions, respectively. These correspond to hydrogen bonding and halogen-halogen interactions, which are crucial for stabilizing the crystal structure. In contrast, a significantly lower contribution (0.8%) was observed for N⋯H contacts, indicating a minimal role of nitrogen in intermolecular stabilization.

Using HRMS, FTIR, 1D and 2D NMR, and UV-Vis spectroscopy, we elucidated the structures of SB-3, SB-4, and SB-5, including the presence of an intramolecular hydrogen bond (IHB). Furthermore, X-ray crystallographic analyses corroborate the SB-5 structure, including the IHB. Altogether, these analyses confirmed the proposed structures for all compounds.

The exclusive formation of SB-3, SB-4, and SB-5 via the *meta* amino group (3-NH_2_) of 3,4-diaminobenzoic acid is consistent with classical substituent effects and basicity-nucleophilicity correlations in anilines. The carboxyl substituent withdraws electron density more strongly at the para position (resonance + inductive) than at *meta* (predominantly inductive), lowering the basicity/nucleophilicity of the 4-NH_2_ relative to 3-NH_2_ (as captured by *para* vs. *meta* Hammett constants for –CO_2_R) [57,58]. Under standard imine-forming conditions, the more basic/nucleophilic aniline site reacts faster and/or is favored at equilibrium, biasing condensation toward 3-NH_2_ [57,58]. In addition, aminobenzoic acids can engage in acid-amine hydrogen-bonding self-association in solution, which may further attenuate the adequate availability of the *para* amino lone pair, depending on the solvent [59]. Together, these electronic and supramolecular factors rationalize the observed meta-selective imine formation in our series.

### 2.4. Electrochemical Behaviors

**Figure 7:**
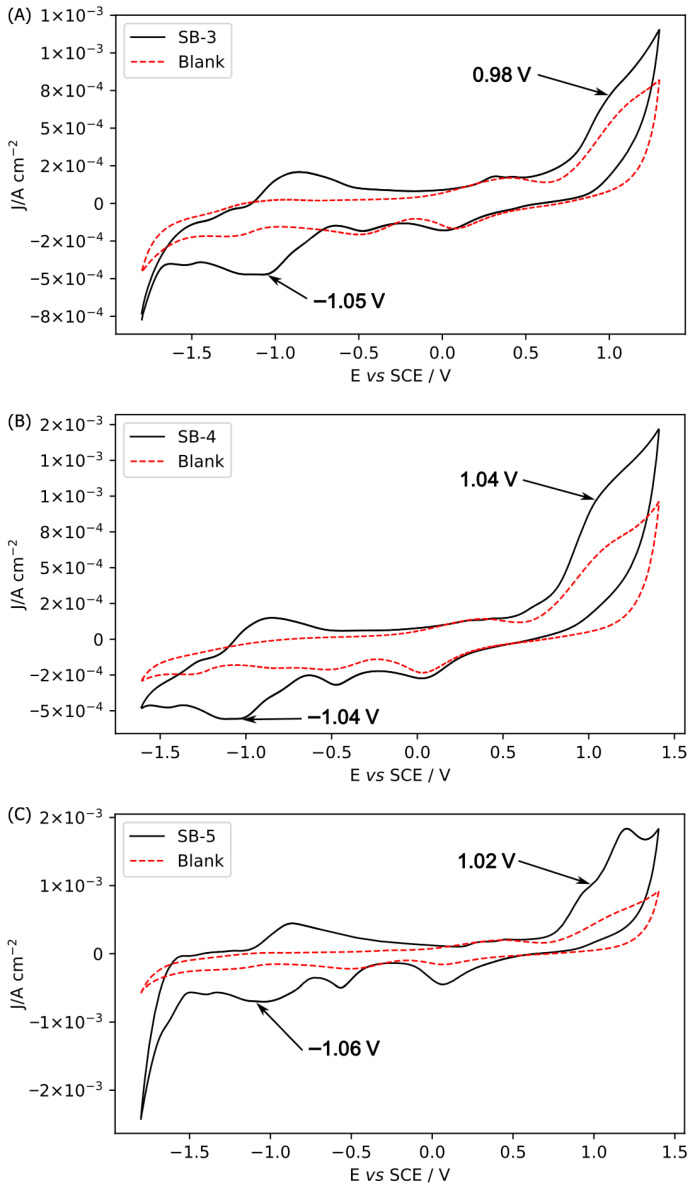
Cyclic voltammetry profiles of SB Schiff bases (—) and blank (---). Interface: Pt|10^−3^ mol L^−1^ of compound + 10^−1^ mol L^−1^ of TBAPF_6_ in anhydrous ACN under an argon atmosphere. Scan rate: 200 mV s^−1^. (**A**) SB-3; (**B**) SB-4; and (**C**) SB-5.

Electrochemical profiling of the new SB series was conducted to elucidate the relationship between their structural features and physicochemical properties, particularly redox potentials. Accordingly, cyclic voltammetry was employed to examine the oxidation and reduction behaviors of SB-3, SB-4, and SB-5, using SB-1 as a reference (previously reported in acetonitrile [3]). Additionally, the cyclic voltammograms of these compounds were compared with those of a control solution of TBAPF_6_ in anhydrous acetonitrile (ACN; Figure 7). To differentiate the redox processes of the compounds from secondary reactions with the solvent (e.g., acetonitrile), it is essential to conduct a working window study (Figures S29–S31) [49]. Comparable profiles have been reported for analogous Schiff bases with an intramolecular hydrogen bond (IHB) [49].

The cyclic voltammetry profile for SB-1 was reported using anhydrous acetonitrile as the solvent (irreversible oxidation peak at 1.23 V and an irreversible reduction peak at −0.90 V) [3]. In that study, SB-1 exhibited an irreversible oxidation peak at 1.03 V. This oxidation is typically attributed to the aminobenzoic ring, most likely at the –NH_2_ group. The significant potential difference is likely due to the electron-withdrawing effect of the carboxylic acid group on SB-1, which increases the energy required to oxidize the amine group. Regarding reduction processes, SB-1 showed an irreversible peak at −1.38 V. This reduction is ascribed to intramolecular reductive coupling of the azomethine group via a self-protonation reaction, as observed for other related Schiff bases. This phenomenon explains the results observed for SB-1. This finding helped assign the observed results for the isostructural Schiff bases to SB-1 (summarized in Table 4).

SB-3, SB-4, and SB-5 constitute a new series of isostructural with SB-1, in which the substituents on the phenolic ring have been modified at the 3 and 5 positions (Figure 1). All these compounds exhibit irreversible oxidation attributed to the aminobenzoic moiety and irreversible reduction due to intramolecular reductive coupling of the azomethine group (Figure 7). All these compounds exhibit irreversible oxidation attributed to the aminobenzoic moiety. Unlike SB-1, which exhibits irreversible reduction, compounds SB-3, SB-4, and SB-5, which incorporate halogen substituents, exhibit reversible reduction. Halogen substituents can significantly influence the redox properties of chemical compounds through their inductive and resonance effects [60]. This suggests that halogen groups may stabilize redox intermediates, thereby facilitating the reversibility of electrochemical reactions, as previously proposed [61,62,63].

The differences observed in the oxidation and reduction peaks depend on the nature of the substituent on the phenolic ring. For instance, *tert*-butyl substituents, due to their considerable bulk, increase steric hindrance and thereby influence the molecule’s reactivity. The appearance of an additional wave during the reverse scan of SB-3, SB-4, and SB-5 (reversible process) causes a shift in the half-wave potential of the reduction to less negative values. As a result, the observed reduction potential for SB-1 differs from that of the other compounds.

In the context of electronic structural analysis, chlorine, bromine, and iodine act as deactivating groups, although their effects differ due to their varying sizes and electronegativities. Chlorine, being the most electronegative, withdraws electron density from the ring and imposes minimal steric hindrance due to its smaller size than bromine and iodine. Although the iodine substituent is larger than the other halogens, it remains smaller than a *tert*-butyl group. In our analysis, the reduction potentials of the three halogenated compounds are very similar despite differences in the electronic effects of the halogen atoms. This may be attributed to the fact that the site of the molecule undergoing reduction is relatively distant from the region where the halogens are located. In other words, the reduction does not occur directly on the phenolic ring.

All tested Schiff bases (SB-1, SB-3, SB-4, and SB-5) exhibit irreversible oxidation peaks consistent with the oxidation of the aminobenzoic ring. However, only SB-1 displays an irreversible reduction peak, whereas SB-3, SB-4, and SB-5 show reversible reduction peaks associated with the azomethine group. The shifts in peak potentials among the derivatives highlight the influence of substituents on the phenolic ring [17,18], where both electronic (electronegativity) and steric effects modulate redox behavior.

The robust structural characterization of SB-3, SB-4, and SB-5, including the confirmation of a stabilizing intramolecular hydrogen bond and distinct redox behavior, provides a strong foundation for investigating their antimicrobial and cytotoxic profiles. In the subsequent sections, we evaluate these compounds against a range of bacterial and fungal pathogens, as well as in mammalian cell viability assays, to elucidate their potential as selective antimicrobial agents.

### 2.5. Analysis of Local Reactivity

**Figure 8:**
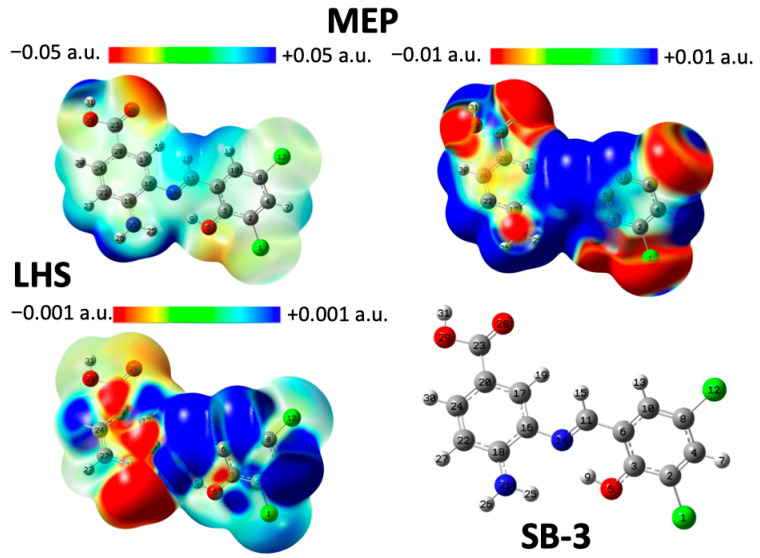
Local reactivity descriptors of SB-3. Molecular electrostatic potential (MEP) and local hypersoftness (LHS) values as 3D pictures. For purposes of qualitative comparison, isovalues of MEP are displayed at ±0.05 *hartree*·*e*^−1^ (or ±0.05 a.u.), at ±0.01 *hartree*·*e*^−1^ (or ±0.01 a.u.), and isovalues of LHS are displayed at ±0.001 *e*^3^·*hartree*^−2^·*bohr*^−3^ (or ±0.001 a.u.). These values of MEP and LHS were projected onto an electron density isosurface of 0.001 *e*·*bohr*^−3^. The corresponding atom numbering is shown on the right.

**Figure 9:**
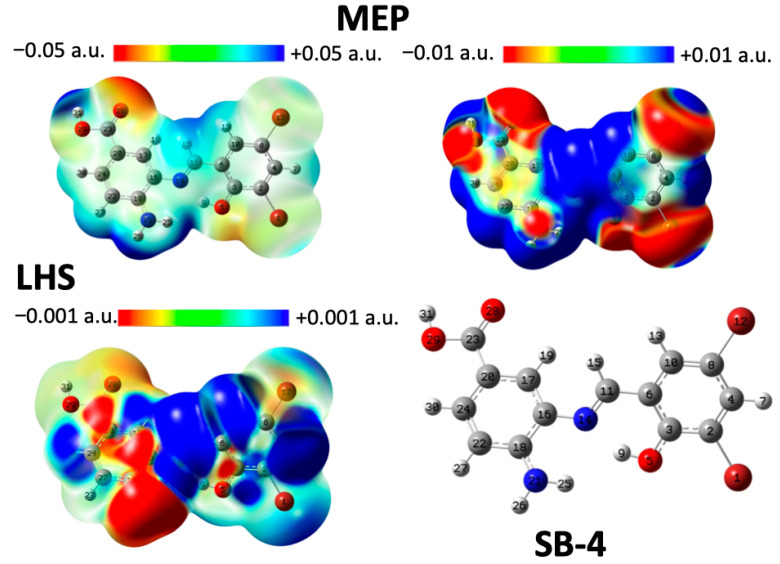
Local reactivity descriptors of SB-4. Molecular electrostatic potential (MEP) and local hypersoftness (LHS) values as 3D pictures. For purposes of qualitative comparison, isovalues of MEP are displayed at ±0.05 *hartree*·*e*^−1^ (or ±0.05 a.u.), at ±0.01 *hartree*·*e*^−1^ (or ±0.01 a.u.), and isovalues of LHS are displayed at ±0.001 *e*^3^·*hartree*^−2^·*bohr*^−3^ (or ±0.001 a.u.). These values of MEP and LHS were projected onto an electron density isosurface of 0.001 *e*·*bohr*^−3^. The corresponding atom numbering is shown on the right.

**Figure 10:**
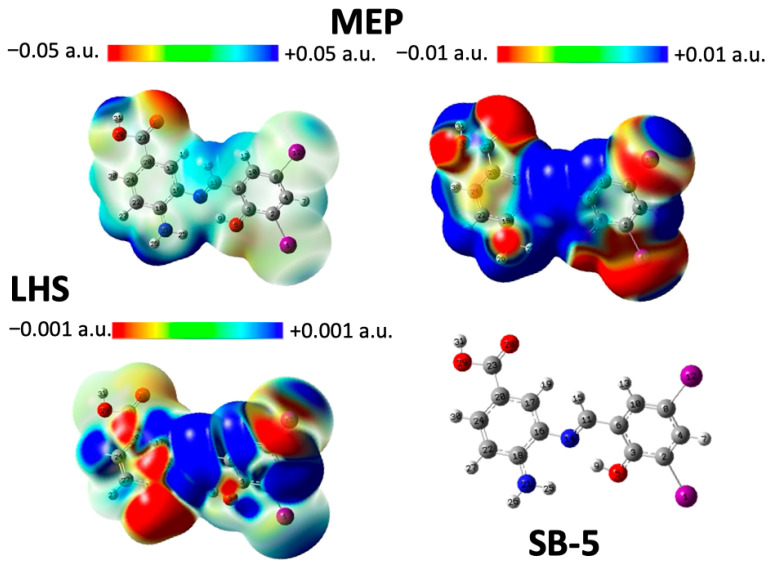
Local reactivity descriptors of SB-5. Molecular electrostatic potential (MEP) and local hypersoftness (LHS) values as 3D pictures. For purposes of qualitative comparison, isovalues of MEP are displayed at ±0.05 *hartree*·*e*^−1^ (or ±0.05 a.u.), at ±0.01 *hartree*·*e*^−1^ (or ±0.01 a.u.), and isovalues of LHS are displayed at ±0.001 *e*^3^·*hartree*^−2^·*bohr*^−3^ (or ±0.001 a.u.). These values of MEP and LHS were projected onto an electron density isosurface of 0.001 *e*·*bohr*^−3^. The corresponding atom numbering is shown on the right.

Density Functional Theory (DFT) [62] was used to study the chemical reactivity and site selectivity of SB-3, SB-4, and SB-5. For this purpose, the molecular electrostatic potential (MEP) and local hypersoftness (LHS) were calculated [64]. The integrated LHS values are expressed in $e^{3}\cdot {hartree}^{-2}$, and atom-specific LHS values can be found in the Supplementary Materials (Table S5). At the B3LYP/df-TZVPPD level of theory [65,66], both molecular electrostatic potential (MEP) and local hypersoftness (LHS) descriptors yielded qualitatively consistent results [67]. LHS was computed exclusively via the finite difference approximation (FDA), the most accurate method currently available for this descriptor, rendering comparisons with the frontier molecular orbital approximation (FMOA) unnecessary [64]. Diffuse functions were essential due to the nature of the FDA, which involves evaluating the system’s energy and electron density in the presence of a weakly bound electron. This requirement applies regardless of whether the structure is anionic. Visualization of MEP and LHS was performed by mapping each descriptor onto a 0.001 a.u. electron density isosurface, following the methodology proposed by Peter Politzer and collaborators [64]. MEP analysis of SB-3, SB-4, and SB-5 consistently revealed the presence of acidic hydrogen atoms (Figure 8, Figure 9 and Figure 10). In SB-3, two such protons were identified: atom 31 (0.0739 a.u.) and atom 26 on the amino group (0.0742 a.u.), both of which were surrounded by electron-deficient regions. The carbonyl oxygen (atom 28) exhibited the most negative potential (−0.0529 a.u.), indicating its susceptibility to electrophilic attack. SB-4 showed similar features, with acidic hydrogens on atoms 31 (0.0741 a.u.) and 26 (0.0745 a.u.), and a carbonyl oxygen at −0.0526 a.u. In SB-5, the acidic hydrogens were located at 0.0738 and 0.0743 a.u., whereas the carbonyl oxygen reached −0.0531 a.u. These findings confirm the presence of consistent acidic and electrophilic regions across all three molecules, with the carbonyl oxygen being the most electron-rich site. LHS analysis identified the imine group (–N=C–H) as the most nucleophilically reactive site in SB-3, SB-4, and SB-5. In SB-3, the highest LHS value (0.0106 a.u.) was observed between hydrogen and carbon (atoms 15 and 11), followed by nitrogen (atom 14) at 0.0050 a.u. A hydrogen atom (atom 7) adjacent to a carbon flanked by two chlorinated carbons showed 0.0053 a.u. The amino nitrogen (atom 21) exhibited the most negative LHS (−0.00284 a.u.), indicating a strong electron-donating capacity. SB-4 followed the same pattern, with maximum LHS values of 0.0105 a.u. (H–C), 0.0049 a.u. (N), and 0.0055 a.u. for a hydrogen near brominated carbons. The amino nitrogen again showed the lowest value (−0.00281 a.u.). In SB-5, the highest LHS was 0.0108 a.u. (H–C), with nitrogen at 0.0051 a.u., and a hydrogen near brominated carbons at 0.0060 a.u. The amino nitrogen reached −0.00245 a.u., confirming its role as the primary electron donor.

On the other hand, global reactivity was assessed using chemical potential (μ), molecular hardness (η), global softness (S), and electrophilicity indices (ω, ω^−^, ω^+^, Δω) [68,69,70,71]. The chemical potential, which indicates the tendency of electrons to escape from equilibrium, followed the trend: SB-5 > SB-3 > SB-4. The molecular hardness, which reflects resistance to charge transfer, decreased in the order SB-3 > SB-4 > SB-5, which is consistent with the increasing polarizability of the halogens from Cl to I. In the case of the global softness (S), which is inversely related to hardness and linked to polarizability, it increased in the order: SB-3 < SB-4 < SB-5, suggesting that heavier halogens enhance overall molecular reactivity. Finally, electrophilicity (ω) showed minimal variation between SB-4 and SB-5, while the electron-donating power (ω^−^) remained nearly identical across all three molecules. This aligns with the LHS findings, which show that amino nitrogen is the primary electron donor, with only slight differences among the compounds. In contrast, the electron-accepting power (ω^+^) increased in the order SB-3 < SB-4 < SB-5, indicating a clear distinction in their ability to accept electrons. However, the net electrophilicity (Δω) showed negligible differences, suggesting that the global electrophilic behavior was not significantly different. Therefore, any experimental differences in reactivity are likely due to local effects, such as larger σ-holes (MEP) and varied donor/acceptor tendencies (LHS). In brief, MEP analysis showed that halogen atoms exhibit little reactivity over a broad potential range. However, when the scale is narrowed, from [−0.05, 0.5] to [−0.01, 0.01], σ-holes become visible and increase in size from Cl to I, reflecting greater polarizability. LHS analysis revealed subtle differences in the halogen behavior. While the chlorine atoms showed minimal variation, the iodine atoms in SB-5 exhibited a stronger electron-donating tendency. These differences were not captured by MEP alone, highlighting LHS’s sensitivity to local reactivity. Together, MEP and LHS offered complementary insights into the electronic effects of halogen substitution in Schiff bases.

The global descriptors revealed an increase in softness and electron-accepting power from SB-3 to SB-5, which may help explain the observed biological activity trends, particularly the enhanced performance of SB-5 (Table S5). These electronic characteristics, such as more pronounced σ-holes and greater polarizability, could account for the antimicrobial selectivity and potency, supporting the correlation between electronic structure and biological activity.

On the other hand, the electrochemical behavior previously described correlates well with the electronic structure revealed by MEP and LHS analyses. Irreversible oxidation is consistently attributed to the aminobenzoic moiety, while reversible reduction is associated with the azomethine group. MEP maps identified the carbonyl oxygen as the most susceptible site for electrophilic attacks, supporting its involvement in oxidation processes. LHS analysis highlighted the imine group as the most susceptible region to nucleophilic attacks, consistent with its role in reduction. The presence of halogen substituents contributes to the stabilization of redox intermediates through electronic effects, thereby facilitating their reversibility. Despite variations in halogen properties, the similarity in reduction potentials suggests that the redox-active sites are spatially distant from the substituent positions. This integrated theoretical and experimental approach provides a comprehensive understanding of how electronic and steric factors govern the redox behavior of these Schiff bases.

### 2.6. Analysis of Antimicrobial Activity

Building on the structural and electrochemical insights obtained, we assessed the antimicrobial efficacy of these Schiff bases. Given that halogen substituents can influence molecular interactions with microbial targets, we evaluated the compounds against both Gram-positive and Gram-negative bacteria. We further extended our analysis to include fungal pathogens and mammalian cell cytotoxicity. These studies were designed to determine whether the favorable redox and structural characteristics translate into selective biological activity.

To systematically assess the antimicrobial properties of the synthesized Schiff bases, we evaluated their activity against a variety of microorganisms, including Gram-negative and Gram-positive bacteria. Gram-negative bacteria are characterized by a complex cell envelope comprising an inner cytoplasmic membrane and an outer membrane, rich in lipopolysaccharides (LPS) and porin proteins, that is separated by a periplasmic space and reinforced by a relatively thin peptidoglycan layer [72]. These features render them resilient to environmental stressors and pose significant challenges for the penetration of antibiotics. Given their wide distribution and clinical relevance in both pathogenic and non-pathogenic contexts [72], we tested the Schiff bases against *Salmonella enterica* serovar Typhi (associated with typhoid fever [73]), *Salmonella enterica* serovar Typhimurium (gastroenteritis [74]), *Escherichia coli* (urinary infections, traveler’s diarrhea [75]), and *Morganella morganii* (an opportunistic pathogen [76]). Our analysis revealed that both the aminobenzoic acid-derived Schiff Bases and their synthetic precursors exhibited no detectable antimicrobial activity against the Gram-negative bacteria tested. To further investigate whether the outer membrane barrier is responsible for this inactivity, we employed *Salmonella enterica* serovar Typhi mutants with enhanced membrane permeability. Specifically, we used mutants lacking the outer membrane protein OmpA (*S.* Typhi Δ*ompA*), a key element in maintaining membrane stability, as well as mutants deficient in the *yibP* gene (*S.* Typhi Δ*yibP*), which is involved in the architecture of the envelope. The absence of these components compromises the integrity of the outer membrane, thereby increasing susceptibility to agents such as vancomycin, an antibiotic that typically cannot penetrate the outer membrane of Gram-negative bacteria [77,78]. Despite employing these mutants, neither the aminobenzoic acid-derived Schiff Bases nor their synthetic precursors exhibited any detectable antimicrobial activity. This lack of activity in mutants designed to compromise the outer membrane barrier suggests that it is not solely due to limited compound penetration. Instead, it indicates that the intrinsic antimicrobial potency of these compounds against Gram-negative bacteria is insufficient.

Based on these results, which showed no antimicrobial effects of the aminobenzoic acid-derived Schiff Bases against the Gram-negative bacteria tested, we proceeded to evaluate their activity against Gram-positive bacteria. Unlike Gram-negative organisms, Gram-positive bacteria lack an outer membrane, an important barrier that can limit compound accessibility, potentially allowing greater penetration of the Schiff bases to their target sites [19,79,80,81]. Accordingly, we assessed the inhibitory effects of these compounds on several Gram-positive species. In this study, we examined *Bacillus subtilis* (a sporulating bacterium that produces a variety of beneficial metabolites [82]), *Streptococcus agalactiae* (associated with neonatal meningitis [83]), *Streptococcus pyogenes* (a causative agent of impetigo, erysipelas, cellulitis, and pharyngitis [84]), *Enterococcus faecalis* (implicated in bloodstream infections and infective endocarditis [85]), *Staphylococcus aureus* (a major human pathogen responsible for a wide range of clinical infections [86]), and *Staphylococcus haemolyticus* (commonly linked to nosocomial infections [87]).

The results summarized in Table 5 indicate that the antimicrobial activity of the Schiff bases is highly dependent on the nature of the halogen substituents. Notably, SB-3 (derived from 3,5-dichlorosalicyaldehyde) and SB-4 (from 3,5-dibromo-2-hydroxybenzaldehyde) generally exhibited no detectable inhibitory effects against several Gram-positive strains, including *Bacillus subtilis*, *Enterococcus faecalis*, and *Staphylococcus aureus* (strains 2, 6, and 7), despite the precursor aldehydes showing activity in some cases. In contrast, the diiodo derivative SB-5 consistently demonstrated measurable antimicrobial activity. Moreover, for both *Staphylococcus aureus* (strains 6 and 7) and *Staphylococcus haemolyticus*, only SB-5 showed inhibition, with MIC values ranging from 8.6 to 14.1 µM.

These findings suggest that incorporating iodine atoms at positions 3 and 5 of the phenolic ring significantly enhances the antimicrobial potency of the Schiff bases. The improved activity of SB-5 may be attributed to iodine’s higher polarizability and favorable steric properties, which likely facilitate more effective interactions with bacterial targets [88,89].

Halogens such as chlorine, bromine, and iodine, when incorporated into small organic molecules, exhibit distinctive properties due to their anisotropic surface charge distributions [90]. These uneven charge distributions create localized regions of positive charge, which facilitate molecular interactions and potentially enhance biological activities, including antimicrobial effects [90]. Iodine is particularly notable for increasing the electropositive surface area when attached to an aromatic ring. This modification not only improves the molecule’s interaction with biological targets but also enhances the lipophilicity of the Schiff base, thereby promoting more effective penetration through microbial lipid membranes and access to intracellular targets [90]. Additionally, iodine substitution may affect the Schiff base’s ability to form hydrogen bonds and participate in other crucial interactions with microbial enzymes or proteins, potentially disrupting essential cellular processes [90]. Moreover, while metal complexes of Schiff bases exhibit antimicrobial activity, the incorporation of iodine into the phenol ring further enhances their efficacy [91].

Overall, our results underscore the critical role of halogen substitution in modulating the bioactivity of Schiff bases, supporting the further development of diiodo derivatives as promising antimicrobial agents.

In general, Schiff bases are evaluated using aerobic or facultative anaerobic bacteria because they are easier to handle. In contrast, studies involving strictly anaerobic bacteria are less common [92] due to the technical challenges of their cultivation and experimental manipulation. To assess the antimicrobial effect of SB-3, SB-4, and SB-5 in anaerobic bacteria, anaerobic cultures of *Clostridioides difficile* (nosocomial antibiotic-associated diarrhea [93]) strain R20291 and *Blautia coccoides* (probiotic [94]) strain ATCC-29236 were prepared in Brain-Heart Infusion (BHI) medium supplemented with 0.1% cysteine, with additional supplements to prevent *C. difficile* sporulation. Following a 16 h incubation under anaerobic conditions, the minimum inhibitory concentrations (MICs) of compounds SB-3, SB-4, and SB-5 were determined, and bacterial growth was assessed by measuring the optical density (OD_600_) at 24 and 48 h.

As shown in Table 6, both *C. difficile* and *B. coccoides* exhibited similar MICs for SB-3 and SB-4 (1.0 µM). For SB-5, *C. difficile* demonstrated a MIC of 0.1 µM, while *B. coccoides* was more sensitive, with a MIC of 0.01 µM. These findings indicate that the antimicrobial potency of the aminobenzoic acid-derived Schiff base against strict anaerobes is both strain- and compound-dependent. The enhanced activity of SB-5 against *C. difficile* and *B. coccoides* suggests that the diiodo substitution increases antimicrobial efficacy in these strains. Overall, these results underscore the critical role of halogen substitution in modulating the antimicrobial properties of these compounds against anaerobic pathogens.

**Figure 11:**
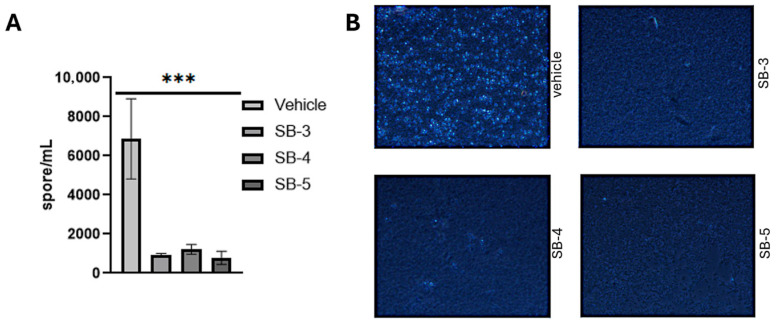
Evaluation of Spore Production of *Clostridioides difficile* Under SB-3, SB-4, and SB-5 Treatment. (**A**) Quantification of spores observed in 10 different fields across three biological replicates. DMSO was used as the vehicle control (10% *v*/*v*). (**B**) Representative images showing the difference in spore counts between the vehicle control (each bright sphere represents a spore) and the spores observed under each treatment condition. *n* = 3; magnification: 1000×; Image width: 60 μm. One-way ANOVA followed by Tukey’s post hoc test, *** *p* < 0.001.

Schiff bases, known for their diverse biological activities, have been extensively studied for their antimicrobial properties. However, their specific impact on the anti-sporulation effect in sporulated bacteria is not directly addressed. A bacterial spore is a dormant, highly resistant structure formed by certain bacteria in response to unfavorable conditions, enabling long-term survival until the environment becomes favorable for growth [93]. To determine whether the Schiff bases SB-3, SB-4, and SB-5 could influence *Clostridioides difficile* sporulation, a key mechanism by which this pathogen persists and spreads, we conducted a sporulation assay at sub-MICs (one-tenth of the respective MICs). This approach enabled us to assess potential inhibitory effects on endospore formation without completely halting bacterial growth, thereby providing insight into the broader antimicrobial potential of these compounds. Figure 11A illustrates the quantification of spores, showing that all three Schiff bases significantly decreased spore counts compared to the vehicle control (DMSO, 10% *v*/*v*) after incubation. Representative images (Figure 11B) further highlight the reduction in bright, spherical spores under each treatment condition relative to the control. Collectively, these findings suggest that even at sublethal doses, SB-3, SB-4, and SB-5 can interfere with *C. difficile* sporulation, indicating a broader antimicrobial potential beyond simple bactericidal or bacteriostatic effects.

Our findings indicate that halogen substitution, particularly iodine, significantly enhances the antimicrobial activity of these aminobenzoic acid-derived Schiff Bases against Gram-positive bacteria, whereas none of the compounds showed activity against Gram-negative strains. These results establish a solid basis for further investigation into the structure-activity relationships of halogenated Schiff bases.

In our series, halogenation (most notably iodination in SB-5) likely enhances biocidal potency through complementary physicochemical effects: (i) greater membrane partitioning/uptake driven by increased lipophilicity upon halogen substitution, a well-established medicinal chemistry strategy for improving cell permeability (with larger halogens typically conferring larger effects) [95,96]; (ii) directional halogen bonding (XB) between σ-hole-bearing C–X groups (I > Br > Cl) and electron-rich sites in proteins or membrane constituents, which strengthens noncovalent recognition and can increase residence time at microbial targets [97,98]; (iii) electronic/redox tuning of the azomethine pharmacophore, as electron-withdrawing halogens polarize the C=N linkage and shift redox potentials, consistent with our cyclic voltammetry trends and with literature correlating substituent (Hammett) effects to redox behavior in related Schiff systems and organic scaffolds [99,100]; and (iv) conformational pre-organization by intramolecular O–H⋯N hydrogen bonding (IHB), which enforces planarity and electronic delocalization favorable for target engagement and SAR read-through across the halogen series [24]. The lack of activity against Gram-negative bacteria at the tested concentrations is also consistent with the outer-membrane permeability barrier, which restricts the entry of hydrophobic agents, even when potent against Gram-positive organisms [101,102].

In addition to their antimicrobial properties, the safety of these compounds was assessed via HeLa cell viability assays (see below). The differential cytotoxic profiles of SB-3, SB-4, and SB-5, when considered in conjunction with their antibacterial activity, highlight the potential for selective targeting. Similarly, antifungal assays against *Botrytis cinerea* provide further context for their broader bioactivity, although the observed fungistatic effects suggest that additional optimization is needed for fungicidal applications.

### 2.7. HeLa Cell Viability Assays

Given that the clinical utility of an antimicrobial agent depends not only on its efficacy against pathogens but also on its safety in host cells, it is crucial to assess the cytotoxicity of candidate compounds early in the development process. The MTT assay, which quantitatively measures cell viability by reducing MTT tetrazolium to formazan by metabolically active cells, is widely recognized as a reliable method for this purpose [103]. In this study, we used the MTT assay to assess the cytotoxicity of the Schiff base derivatives SB-3, SB-4, and SB-5 on HeLa cells. This evaluation is essential because a compound’s antimicrobial potential must be balanced against any adverse effects on mammalian cell viability to ensure a favorable therapeutic index. Moreover, understanding the concentration thresholds at which these compounds become cytotoxic provides invaluable guidance for dosage optimization and further structural modifications. Across all three compounds, HeLa viability displayed clear concentration-dependent behavior consistent with dose–response curves over the tested ranges (Figures S32–S34).

Across the panel, antibacterial effects were observed at micromolar exposures (Table 5), whereas HeLa cell viability (MTT) remained largely preserved within the millimolar ranges tested for SB-3, SB-4, and SB-5 (Figures S32–S34; vehicles matched to each series’ top dose). Importantly, the supplementary datasets did not identify a lower toxic threshold for HeLa cells. Thus, while the data are consistent with a separation between antibacterial activity and mammalian viability under our conditions, the magnitude of that separation cannot be quantified here and should not be overinterpreted. Overall, these findings emphasize the necessity of early cytotoxicity screening to inform dose selection and guide subsequent relationship studies.

### 2.8. Cytotoxicity Assays Botrytis cinerea

Given the critical need for novel antifungal agents in agriculture and related fields, we extended our evaluation of these Schiff bases to *Botrytis cinerea*, a necrotrophic fungal pathogen responsible for significant crop losses [104]. Previous studies by our group have demonstrated that structurally related Schiff bases (e.g., L1) exhibit promising antifungal activity against *B. cinerea* at 26 °C [105,106], justifying further investigation of the new derivatives SB-3, SB-4, and SB-5 as potential biocides.

In this study, *B. cinerea* strain B05.10 cultures were treated in vitro with a single dose of each compound (SB-3 at 30.7 µM, SB-4 at 37.9 µM, and SB-5 at 26.3 µM) for 6 days. Although none of the compounds achieved complete growth inhibition (i.e., a fungicidal effect), a fungistatic effect was observed during the initial 3 days of treatment, as evidenced by a noticeable reduction in mycelial growth compared to the DMSO vehicle control. By day 6, however, the fungal growth recovered, indicating that while the compounds delayed spore germination and mycelial expansion, they did not wholly suppress fungal viability under the tested conditions (Figure S35).

In conclusion, although SB-3, SB-4, and SB-5 did not exhibit complete fungicidal activity against *Botrytis cinerea*, the transient fungistatic effect observed during the early stages of growth suggests that these compounds may influence fungal development under certain conditions. However, the overall limited antifungal activity suggests that further structural modifications or alternative application strategies are necessary to enhance their efficacy as potential biocidal agents.

## 3. Materials and Methods

### 3.1. Materials and Instruments

All reagents for the synthesis were purchased from Aldrich and used without further purification. Fourier-transform infrared (FTIR) spectra were recorded on a Tracer-100 FT-IR spectrophotometer (Shimadzu, Kyoto, Japan) using KBr pellets. Proton nuclear magnetic resonance (^1^H NMR), HHCOSY, carbon-13 nuclear magnetic resonance (^13^C NMR), and DEPT-45 spectra for SB-3 to SB-5 were acquired on a Bruker AVANCE 400 spectrometer (Bruker, Billerica, MA, USA) at 25 °C (400 MHz for ^1^H and 100 MHz for ^13^C), with chemical shifts reported in ppm in deuterated DMSO. Melting points were determined using a Stuart Scientific SMP3 apparatus (Stuart, Staffordshire, UK) in open capillary tubes and were uncorrected. Elemental analyses (CHNS) were performed on a Thermo Flash 2000 Series instrument (Thermo Scientific, Waltham, MA, USA) equipped with a thermal conductivity detector (TCD).

UV-Vis absorption spectra of SB-3, SB-4, and SB-5 were recorded on an Agilent 8454 diode-array spectrophotometer (Agilent Technologies, Santa Clara, CA, USA) over the range of 250–650 nm in air-saturated methanol and DMSO (spectroscopic grade). Molar absorptivities (ε) at each λ_max_ were obtained from Beer-Lambert plots (slope of A vs. c, 5–50 μM) using 1.0 cm quartz cuvettes; solvent blanks were subtracted prior to acquisition. Steady-state emission spectra were collected at room temperature on a FluoroMax-4 spectrofluorometer (Horiba Jobin-Yvon, Edison, NJ, USA), with excitation set near the maximum absorption of each compound in the corresponding solvent. Fluorescence quantum yields (Φ) were determined by the comparative method using 9,10-diphenylanthracene in cyclohexane (Φ = 1.00; λ_exc_ = 366 nm) as the reference standard [107,108].

High-resolution mass spectrometry (HRMS) analyses were carried out using a Bruker Compact quadrupole time-of-flight (qTOF) mass spectrometer (Germany) coupled with an Apollo II ion funnel electrospray ionization (ESI) source. Mass spectra were acquired by direct infusion, with calibration performed using the Chrom Tec LC/MS calibration standard (Agilent Technologies, Santa Clara, CA, USA; Fondequip EQM170172).

For the electrochemical studies, the working solution consisted of 1 × 10^−3^ M of the test compounds and 1 × 10^−1^ M tetrabutylammonium hexafluorophosphate (TBAPF_6_) as the supporting electrolyte in anhydrous DMF. Prior to each experiment, the solution was purged with high-purity argon, and an argon atmosphere was maintained throughout, as previously described. A polycrystalline, non-annealed platinum disc (2 mm in diameter) served as the working electrode, while a platinum gauze, separated from the main compartment by a fine sintered glass frit, served as the counter electrode. All potentials are referenced to an Ag/AgCl electrode in tetramethylammonium chloride, calibrated to match the potential of a saturated calomel electrode (SCE) at room temperature [105,109]. Electrochemical experiments were conducted at ambient temperature using a CHI900B bipotentiostat (CH Instruments, Austin, TX, USA) with control and data acquisition performed via CHI 9.12 software.

### 3.2. Procedure for Preparing SB-3, SB-4, and SB-5

All starting materials were procured from Merck and Aldrich and used without further purification. The Schiff bases SB-3, SB-4, and SB-5 were synthesized via the direct condensation of 3,4-diaminobenzoic acid with the corresponding aldehydes at room temperature under continuous stirring for over 24 h (Scheme S1). The reactions consistently afforded yields exceeding 75%.

#### 3.2.1. Synthesis of (E)-4-Amino-3-((3,5-di-chloride-2-hydroxybenzylidene)amino) Benzoic Acid (SB-3)

SB-3 was synthesized via the condensation of 3,4-diaminobenzoic acid (precursor A) with 3,5-dichloro-2-hydroxybenzaldehyde (precursor B) in a 1:1 molar ratio. The reaction was carried out in 20 mL of methanol at room temperature, with continuous stirring for 24 h, without heating or an inert atmosphere (Scheme S1). Following reaction completion, the precipitate was filtered, washed with a 50:50 (*v*/*v*) mixture of ethanol and diethyl ether, and dried under vacuum. This procedure afforded a light orange, amorphous solid in 78% yield with a melting point of 242 °C (decomposition observed).

FTIR analysis (KBr, cm^−1^) revealed characteristic absorptions at 3479 and 3379 (νOH), 3070 and 2974 (νNH_2_), 2549 (νC=C), 1683 (νC=O), 1625 (νN=C), and 1600 (νC=C). The ^1^H NMR spectrum (400 MHz, DMSOd_6_, ppm) displayed signals at δ 13.65 (s, 1H, –OH), 12.30 (s, 1H, –COOH), 8.97 (s, 1H, H4), 7.86 (s, 1H, H3), 7.72 (s, 2H, H5 and H6), 7.64 (d, J = 8.4 Hz, 1H, H1), 6.80 (d, J = 8.5 Hz, 1H, H2), and 6.01 (s, 2H, –NH_2_). The ^13^C NMR spectrum (400 MHz, DMSOd_6_, ppm) showed resonances at δ 167.71 (–COOH), 161.02 (–N=C–), 155.00, 148.31, 132.68, 132.56, 130.86, 122.92, 122.53, 121.20, 118.85, and 114.87, while the DEPT45 spectrum confirmed signals at δ 160.86, 132.58, 131.19, 130.61, 120.82, and 114.92. UV/Vis spectroscopy performed in methanol at room temperature revealed absorption maxima at 278 nm (ε = 5182.00 M^−1^ cm^−1^) and 392 nm (ε = 4950.88 M^−1^ cm^−1^), whereas in DMSO the maxima were observed at 287 nm (ε = 13,731.67 M^−1^ cm^−1^) and 410 nm (ε = 14,869.74 M^−1^ cm^−1^). High-resolution mass spectrometry (ESI^+^) provided an [M + H]^+^ ion at *m*/*z* 324.9973, which is in good agreement with the calculated value of 324.0068 for C_14_H_10_Cl_2_N_2_O_3_.

#### 3.2.2. Synthesis of (E)-4-Amino-3-((3,5-di-bromide-2-hydroxybenzylidene)amino) Benzoic Acid (SB-4)

SB-4 was synthesized by condensing 3,4-diaminobenzoic acid (precursor A) with 3,5-dibromo-2-hydroxybenzaldehyde (precursor C) following the same procedure as described in Section 3.2.1. The reaction was carried out in methanol at room temperature under continuous stirring for 24 h. After completion, the resulting precipitate was filtered, washed with a 50:50 (*v*/*v*) mixture of ethanol and diethyl ether, and dried under vacuum, affording a light orange amorphous solid in 80% yield with a melting point of 237–238 °C.

FTIR analysis (KBr, cm^−1^) showed characteristic absorptions at 3483 and 3381 (νOH), 3064 and 2972 (νNH_2_), 2549 (νC=C), 1681 (νC=O), 1622 (νN=C), and 1597 (νC=C). The ^1^H NMR spectrum (400 MHz, DMSO-d_6_, ppm) displayed signals at δ 13.84 (s, 1H, –OH), 12.33 (s, 1H, –COOH), 8.94 (s, 1H, H4), 7.99 (s, 1H, H3), 7.92 (s, 1H, H5), 7.72 (s, 1H, H6), 7.64 (d, J = 8.3 Hz, 1H, H1), 6.81 (d, J = 8.5 Hz, 1H, H2), and 6.00 (s, 2H, –NH_2_). The ^13^C-NMR spectrum (400 MHz, DMSO-d_6_, ppm) exhibited resonances at δ 168.02 (–COOH), 161.32 (C=N), 156.36, 148.00, 137.61, 134.56, 133.24, 130.90, 122.55, 120.89, 118.86, 114.87, 111.58, and 109.89. The DEPT-45 spectrum confirmed carbon signals at δ 161.26, 137.63, 134.56, 130.61, 128.35, 120.83, and 114.65.

UV/Vis spectroscopy performed in methanol at room temperature revealed absorption maxima at 279 nm (ε = 16,293.16 M^−1^ cm^−1^) and 393 nm (ε = 5587.50 M^−1^ cm^−1^); in DMSO, the maxima were observed at 288 nm (ε = 16,293.16 M^−1^ cm^−1^) and 407 nm (ε = 5587.50 M^−1^ cm^−1^). High-resolution mass spectrometry (ESI^+^) yielded an [M + H]^+^ ion at *m*/*z* 412.9035, which is consistent with the calculated value of 411.9058 for C_14_H_10_Br_2_N_2_O_3_.

#### 3.2.3. Synthesis of (E)-4-Amino-3-((3,5-di-iodide-2-hydroxybenzylidene)amino) Benzoic Acid (SB-5)

SB-5 was synthesized via direct condensation of 3,4-diaminobenzoic acid with 3,5-di-iodide-2-hydroxybenzaldehyde under the conditions described in Section 3.2. Following the reaction, an orange-colored, amorphous solid was obtained in 79% yield, with a melting point of 175 °C (decomposition observed).

FTIR analysis (KBr, cm^−1^) revealed characteristic absorptions at 3462 and 3367 (νOH), 3064 and 2972 (νNH_2_), 2550 (νC=C), 1680 (νC=O), 1616 (νN=C), and 1595 (νC=C). The ^1^H NMR spectrum (400 MHz, DMSO-d_6_, ppm) showed signals at δ 13.99 (s, 1H, –OH), 12.33 (s, 1H, –COOH), 8.85 (s, 1H, H4), a multiplet at 8.21–8.06 (m, 2H, H3 and H5), 7.71 (s, 1H, H6), 7.64 (d, J = 8.4 Hz, 1H, H1), 6.80 (d, J = 8.5 Hz, 1H, H2), and 5.98 (s, 2H, –NH_2_). The ^13^C NMR spectrum (400 MHz, DMSO-d_6_, ppm) exhibited resonances at δ 167.70 (–COOH), 161.68 (–N=C–), 160.04, 148.30, 147.62, 141.33, 132.95, 130.91, 122.24, 121.57, 118.86, and 115.25. DEPT-45 analysis confirmed carbon signals at δ 161.48, 148.31, 141.33, 135.11, 130.62, 121.07, and 114.94.

UV/Vis spectroscopy performed in methanol at room temperature revealed absorption maxima at 282 nm (ε = 5493.82 M^−1^ cm^−1^) and 395 nm (ε = 6274.50 M^−1^ cm^−1^); in DMSO, the corresponding maxima were observed at 290 nm (ε = 17,415.71 M^−1^ cm^−1^) and 407 nm (ε = 17,439.92 M^−1^ cm^−1^). High-resolution mass spectrometry (ESI^+^) yielded an [M + H]^+^ ion at *m*/*z* 508.8863, which aligns well with the calculated value of 507.8781 for C_14_H_10_I_2_N_2_O_3_.

### 3.3. Structure Determination

Crystals suitable for X-ray diffraction analysis of compound SB-5 were obtained as described previously and mounted using MiTeGen MicroMounts. Table 2 summarizes the experimental and crystallographic data for SB-5. At the same time, selected bond distances and angles for all studied compounds are provided in Table S4. Intramolecular interactions are detailed in Table 3, and additional information on the crystal, data collection, and structure solution is available in the Supplementary Materials (Table S4).

Intensity data were collected at room temperature using a Bruker Smart Apex diffractometer equipped with a two-dimensional CMOS Photon100 detector (Bruker AXS, Karlsruhe, Germany) and a graphite monochromator, employing Mo-Kα radiation. Data were acquired with a frame separation of 0.31° and an exposure time of 10 s per frame at 296 K. Data integration was performed using APEX3 software, with absorption corrections applied using SADABS.

The structure of SB-5 was solved by the Patterson method and refined with the ShelXl package via full-matrix least-squares minimization on F^2^, as implemented in Olex2 [110,111]. All non-hydrogen atoms were refined anisotropically, and hydrogen atoms were positioned in idealized locations. A solvent mask was applied, revealing 144 electrons in a volume of 576 Å^3^ within one void per unit cell, consistent with the presence of 3.6 water molecules per formula unit.

### 3.4. Hirshfeld Surface Analysis

Intermolecular interactions within the crystalline lattice were examined using Hirshfeld surface analysis, a technique that quantifies the contribution of each atom’s electron density (the “promolecule”) to the overall electron density of the crystal (the “procrystal” system). This method utilizes a normalized contact distance, *d_norm_*, defined as (Equation (1)):

(1) $$d_{norm}=\;\frac{d_{i}-r_{i}^{VdW}}{r_{i}^{VdW}}+\frac{d_{e}-r_{e}^{VdW}}{r_{e}^{VdW}}$$

where *d_i_* and *d_e_* represent the distances from the surface to the nearest interior and exterior nuclei, respectively, and *r^VdW^* is the corresponding van der Waals radius. Hirshfeld surface analysis has become a powerful tool for elucidating the variety of intermolecular interactions in crystal packing. Moreover, the corresponding two-dimensional fingerprint plots [112,113,114,115] allow for a quantitative comparison of these interactions by highlighting regions of close and distant contacts.

In this study, CrystalExplorer 17.5 (http://hirshfeldsurface.net/, Last accessed: 1 October 2025) was employed to calculate the Hirshfeld surfaces and generate the associated 2D fingerprint plots for the L1-4 compounds using the crystallographic information files (CIFs) as input. The electrostatic potential was mapped onto Hirshfeld surfaces using the 3-21G basis set at the Hartree–Fock level, over a range of ±0.002 a.u., using the TONTO computational package integrated within CrystalExplorer. For the generation of the fingerprint plots, hydrogen-bond lengths were normalized to standard neutron diffraction values (C–H = 1.083 Å, N–H = 1.009 Å, O–H = 0.983 Å) [116].

### 3.5. Antimicrobial Activity

#### 3.5.1. Minimum Inhibitory Concentration (MIC) for Aerobic and/or Facultative Bacteria

The in vitro antimicrobial activity of compounds SB-3 to SB-5, along with the corresponding precursors (A: 3,4-diaminobenzoic acid; B: 3,5-dichloride-2-hydroxybenzaldehyde; C: 3,5-dibromide-2-hydroxybenzaldehyde; and D: 3,5-di-iodide-2-hydroxybenzaldehyde), was evaluated against a panel of clinical Gram-positive isolates, including *Bacillus subtilis*, *Streptococcus agalactiae*, *Streptococcus pyogenes*, *Enterococcus faecalis*, *Staphylococcus aureus*, and *Staphylococcus haemolyticus*, obtained from the Hospital Clínico de la Universidad de Chile (Santiago, Chile). In addition, the activity against Gram-negative pathogens was assessed using *Salmonella enterica* subsp. *enterica* serovar Typhimurium (ATCC14028s) [68,69], *Salmonella enterica* subsp. *enterica* serovar Typhi STH2370 and its Δ*ompA* and Δ*yibP* mutants [77,117,118], as well as clinical isolates of *Escherichia coli* and *Klebsiella pneumoniae*. MIC is defined as the lowest concentration of the tested compound at which no microbial growth is observed after incubation [119]. MIC determinations were performed as described in [3]. Briefly, microorganisms were cultured in Luria–Bertani (LB) broth (10 g/L Bacto tryptone, 5 g/L Bacto yeast extract, and 5 g/L NaCl) at 37 °C with shaking until an optical density at 600 nm (OD_600_) of 1.4 was reached, indicating the onset of the stationary phase. Microbial suspensions, standardized to 0.5 McFarland in PBS, were subsequently diluted 1:1000 in LB broth. The inoculated wells were incubated at 37 °C for 24 h. Stock solutions of the compounds were prepared in dimethyl sulfoxide (DMSO), and DMSO alone served as the vehicle control. A compound was considered to exhibit detectable antimicrobial activity only if its inhibitory effect exceeded that of the vehicle control. All experiments were performed in biological and technical triplicate.

#### 3.5.2. Minimum Inhibitory Concentration (MIC) for Anaerobic Bacteria

The in vitro antimicrobial activity of compounds SB-3, SB-4, and SB-5 against anaerobic bacteria was evaluated using *Clostridioides difficile* strain R20291 and *Blautia coccoides* strain ATCC 29236. These strains were cultured under strictly anaerobic conditions in Brain–Heart Infusion (BHI) medium supplemented with 0.1% cysteine, using a COY Lab Type B anaerobic chamber (gas composition: 87% N_2_, 5.4% CO_2_, and 7.6% H_2_) at 37 °C without agitation. To prevent contamination and sporulation of *C. difficile*, the solid BHI medium was further supplemented with 8 µg/mL cefoxitin, 250 µg/mL cycloserine, 0.2% glucose, 0.2% fructose, and 0.2% taurocholate [120,121]. Overnight cultures (16 h) were utilized to determine the MIC. Stock solutions of SB-3, SB-4, and SB-5 were prepared at a concentration of 10^−4^ M in DMSO and serially diluted tenfold. MIC assays were performed in microtiter plates with at least four technical replicates. To further inhibit sporulation in *C. difficile* during the assay, the medium was supplemented with 0.2% fructose, 0.2% glucose, and 0.2% taurocholate. Cultures were adjusted to an initial optical density at 600 nm (OD_600_) of 0.02 and incubated at 37 °C under anaerobic conditions. Bacterial growth was assessed by measuring the OD_600_ at 24 and 48 h. A compound was considered to exhibit detectable antimicrobial activity only if its inhibitory effect exceeded that of the vehicle control. All experiments were performed in biological and technical triplicate.

#### 3.5.3. Sporulation Assay

*Clostridioides difficile* spore production was evaluated using an overnight culture grown in Brain-Heart Infusion (BHI) medium supplemented with 0.2% fructose and 0.2% taurocholate under anaerobic conditions for 16 h. Subsequently, sub-MICs of each compound (SB-3 at 10^−7^ M, SB-4 at 10^−7^ M, and SB-5 at 10^−8^ M) were added, and the cultures were incubated for an additional 8 h. A 1:500 dilution of each culture was then plated onto a 70:30 sporulation medium (63 g/mL Bacto peptone [BD Difco], 3.5 g/mL proteose peptone [BD Difco], 0.7 g/mL ammonium sulfate [(NH_4_)_2_SO_4_], 1.06 g/mL Tris base, 11.1 g/mL brain heart infusion extract [BD Difco], and 1.5 g/mL yeast extract [BD Difco] and 2.48 mM L-cysteine [120] and incubated at 37 °C under anaerobic conditions for 120 h. Spores were harvested by resuspending the cells in 1 mL of 1× PBS, fixed in agarose plugs, and visualized using phase-contrast microscopy at 1000× magnification with immersion oil. Spore quantification was performed in 10 different fields across three biological replicates using ImageJ software 1.54p [122,123].

#### 3.5.4. MTT Assay

HeLa cells were obtained from ATCC, a certified supplier of authenticated cell lines and microbial strains for biomedical research. HeLa cell viability was assessed using the MTT assay [124] in a 96-well plate format, with 35,000 cells seeded per well. Cells were cultured in Dulbecco’s Modified Eagle Medium (DMEM) supplemented with 10% fetal bovine serum, 1× penicillin/streptomycin, and 1× antibiotic/antimycotic, and maintained at 37 °C in a humidified atmosphere with 5% CO_2_. Stock solutions of compounds SB3, SB4, and SB5 were prepared by dissolving precise amounts of each in DMSO. Thus, final concentrations of 169.17 mM SB3, 133.00 mM SB4, and 108.27 mM SB5 were obtained. For SB3, they were serially diluted to achieve test concentrations of 0.154, 0.308, 0.615, 1.230, and 1.845 mM. For SB4, they were serially diluted to achieve test concentrations of 0.121, 0.242, 0.483, 0.966, and 1.449 mM. For SB5, they were serially diluted to achieve test concentrations of 0.098, 0.197, 0.394, 0.787, and 1.181 mM. Subsequently, 200 µL of each dilution was added to the respective wells, and the plates were incubated at 37 °C with 5% CO_2_ for 24 h. Following incubation, a working solution of MTT (5 mg/mL diluted 1:10 in culture medium) was added to each well, and the plates were incubated for an additional 3 h to allow viable cells to convert MTT into formazan crystals. Finally, 100 µL of DMSO was added to each well to solubilize the formazan, and the resulting solution was thoroughly mixed to ensure complete dissolution. Absorbance was measured at 570 nm and 630 nm using a BioTek microplate reader (Agilent BioTek Technologies, Santa Clara, CA, USA), and cell viability was quantified from the resulting absorbance values.

#### 3.5.5. *Botrytis cinerea* Inhibition Assay

*Botrytis cinerea* B05.10 (a standard laboratory strain [125]) was obtained from the Phytopathogenic Fungi Laboratory at Andrés Bello University (Santiago, Chile). Stock cultures were established by inoculating Petri dishes containing Potato Dextrose Agar (PDA, Difco) and incubating them at 26 °C in the dark for 3 days. After incubation, the plates were stored at 4 °C and later used as inocula for subsequent experiments.

To evaluate the antifungal activity of compounds SB-3, SB-4, and SB-5, we assessed their effect on the growth of mycelia. Petri dishes (90 mm in diameter) containing 20 mL of PDA (Difco) were supplemented with 0.0307 mM of SB-3, 0.0379 mM of SB-4, or 0.263 mM of SB-5 (10 ppm of each compound), dissolved in DMSO (Merck). A medium without any compound served as the negative control. The dishes were then incubated in the dark at 26 ± 2 °C for six days. Initial growth measurements were recorded, and after continued incubation under identical conditions, final growth was assessed by measuring the colony diameter (cm). All experiments were performed in biological triplicate, with both incubation and measurements conducted in darkness to prevent light-induced growth stimulation [126].

#### 3.5.6. Statistical Analysis for Biological Assays

All data are expressed as the mean ± standard error of the mean (SEM) based on three independent biological replicates, each performed with three technical replicates. Statistical comparisons were conducted using one-way ANOVA followed by Tukey’s post hoc test.

### 3.6. Computational Details

Computed 3D maps of molecular electrostatic potential (MEP) and local hyper-softness (LHS) were generated at the B3LYP/Def2-TZVPPD level of theory [65,127,128]. MEP calculations were routinely performed using Gaussian 09. For LHS, the Cubegen and Cubman utilities (Gaussian, Wallingford, CT, USA) were employed to generate the required cube files. For more details, see ESI.

## 4. Conclusions

We synthesized and validated three aminobenzoic acid-derived Schiff bases (SB-3, SB-4, SB-5) bearing intramolecular hydrogen bonding, confirming their structures by FTIR, ^1^H/^13^C NMR, UV-Vis, and HRMS (with single-crystal X-ray/Hirshfeld analysis for SB-5). Halogen substitution tuned physicochemical and redox behavior (cyclic voltammetry), in agreement with MEP/LHS descriptors, establishing clear structure–property links. Biocidal assays revealed selective antibacterial activity against Gram-positive and strict anaerobic bacteria, led by the diiodo analogue SB-5 (low µM MICs), with no detectable effect on Gram-negative strains at the tested doses; SB-5 also showed a more favorable HeLa profile than SB-4. Antifungal testing against *Botrytis cinerea* showed only a transient fungistatic effect. Overall, iodine substitution most effectively enhances the electronic/redox features, as well as antibacterial potency, positioning SB-5 as a selective lead for further optimization.

## Figures and Tables

**Table 1:** Data from UV–vis of SB-3, SB-4, and SB-5 in methanol and DMSO.

Compound	Solvent	λ_max_, nm	ε, mol^−1^ dm^3^ cm^−1^	Assignment
SB-3	MeOH	278 392	5182.00 4950.88	n → π * and π → π * π → π *
SB-4	MeOH	279 393	16,293.16 5587.50	n → π * and π → π * π → π *
SB-5	MeOH	282 395	5493.82 6274.50	n → π * and π → π * π → π *
SB-3	DMSO	287 410	13,731.67 14,869.74	n → π * and π → π * π → π *
SB-4	DMSO	288 407	17,347.46 5571.82	n → π * and π → π * π → π *
SB-5	DMSO	290 407	17,415.71 17,439.92	n → π * and π → π * π → π *

**Table 2:** Crystal data collection and structure refinement parameters for compound SB-5.

Compound	SB-5
Empirical formula	C_14_H_10_I_2_N_2_O_3_
Formula weight (g mol^−1^)	508.04
Temperature (K)	296.15
Crystal system	monoclinic
Space group	P2_1_/c
a (Å)	15.9861 (12)
b (Å)	4.6188 (4)
c (Å)	27.612 (2)
α (°)	90
β (°)	105.867 (3)
γ (°)	90
Volume (Å^3^)	1961.1 (3)
Z	4
ρ_calc_ (g cm^−3^)	1.721
μ (mm^−1^)	3.215
F (000)	952.0
Crystal size (mm^3^)	0.075 × 0.017 × 0.014
Radiation	MoKα (λ = 0.71073)
2Θ range for data collection (°)	3.066 to 50
Index ranges	−19 ≤ h ≤ 19, −5 ≤ k ≤ 5, −32 ≤ l ≤ 32
Reflections collected	42,895
Independent reflections	3460 [R_int_ = 0.1428, R_sigma_ = 0.0611]
Data/restraints/parameters	3460/0/193
Goodness-of-fit on F^2^	1.097
Final R indexes [I ≥ 2σ (I)]	R_1_ = 0.0579, wR_2_ = 0.1530
Final R indexes [all data]	R_1_ = 0.1130, wR_2_ = 0.1877
Largest diff. peak/hole/e Å^−3^	1.44/−0.63

**Table 3:** Intramolecular and intermolecular hydrogen-bonding interaction parameters for compound SB-5.

D-H⋅⋅⋅A	D-H (Å)	H⋅⋅⋅A (Å)	D⋅⋅⋅A (Å)	∠D-H⋅⋅⋅A (°)
O(1)-H(1)⋅⋅⋅N(1)	0.82	1.80	2.529 (11)	148.0
O(3)-H(3)⋅⋅⋅O(2) ^1^	0.82	1.81	2.616 (10)	168.4
N(2)-H(2B)⋅⋅⋅O(1) ^2^	0.86	2.18	2.965 (11)	152.4

^1^ 1 − X, −1 − Y, 1 − Z; ^2^ 1 − X, −1/2 + Y, 1/2 − Z.

**Table 4:** Summarized Irreversible Oxidation and Reduction (in Volts) of SB-1, SB-3, SB-4, and SB-5 in anhydrous dimethylformamide (DMF) under an argon atmosphere.

Compound	Ox ^i^_(irr)_	Red ^i^_(rev)_
SB-3	0.98 V	−1.05 V
SB-4	1.04 V	−1.04 V
SB-5	1.02 V	−1.06 V

**Table 5:** Determination of the Minimal Inhibitory Concentration (MIC) of aminobenzoic acid-derived Schiff Bases (SB-3 to SB-5) of Gram-positive bacteria (*n* = 3).

Species	Precursor 1	MIC Precursor 1 (µM)	Precursor 2	MIC Precursor 2 (µM)	Aminobenzoic Acid-Derived Schiff Base	MIC Aminobenzoic Acid-Derived Schiff Base (µM)
*Bacillus subtilis*	3,5-dichlorosalicyaldehide	No effect	3,4-diaminobenzoic	No effect	SB-3	No effect
	3,5-dibromo-2-hydroxybenzaldehyde	No effect	3,4-diaminobenzoic	No effect	SB-4	No effect
	2-hydroxy-3,5-diiodobenzaldehyde	6.3 ± 0.0	3,4-diaminobenzoic	No effect	SB-5	12.6 ± 0.0
*Streptococcus agalactiae*	3,5-dichlorosalicyaldehide	25.3 ± 0.0	3,4-diaminobenzoic	No effect	SB-3	No effect
	3,5-dibromo-2-hydroxybenzaldehyde	17.3 ± 2.3	3,4-diaminobenzoic	No effect	SB-4	25.3 ± 0.0
	2-hydroxy-3,5-diiodobenzaldehyde	3.9 ± 0.5	3,4-diaminobenzoic	No effect	SB-5	6.3 ± 1.0
*Streptococcus pyogenes*	3,5-dichlorosalicyaldehide	20.5 ± 2.3	3,4-diaminobenzoic	No effect	SB-3	20.5 ± 2.3
	3,5-dibromo-2-hydroxybenzaldehyde	9.4 ± 1.1	3,4-diaminobenzoic	No effect	SB-4	5.5 ± 0.5
	2-hydroxy-3,5-diiodobenzaldehyde	3.4 ± 0.7	3,4-diaminobenzoic	No effect	SB-5	3.5 ± 0.3
*Enterococcus faecalis*	3,5-dichlorosalicyaldehide	50.5 ± 0.0	3,4-diaminobenzoic	No effect	SB-3	No effect
	3,5-dibromo-2-hydroxybenzaldehyde	50.5 ± 0.0	3,4-diaminobenzoic	No effect	SB-4	No effect
	2-hydroxy-3,5-diiodobenzaldehyde	10.2 ± 1.1	3,4-diaminobenzoic	No effect	SB-5	14.1 ± 1.5
*Staphylococcus aureus* strain 2	3,5-dichlorosalicyaldehide	No effect	3,4-diaminobenzoic	No effect	SB-3	No effect
	3,5-dibromo-2-hydroxybenzaldehyde	12.6 ± 0.0	3,4-diaminobenzoic	No effect	SB-4	No effect
	2-hydroxy-3,5-diiodobenzaldehyde	6.3 ± 0.0	3,4-diaminobenzoic	No effect	SB-5	12.6 ± 0.0
*Staphylococcus aureus* strain 6	3,5-dichlorosalicyaldehide	50.5 ± 0.0	3,4-diaminobenzoic	No effect	SB-3	No effect
	3,5-dibromo-2-hydroxybenzaldehyde	11.8 ± 0.7	3,4-diaminobenzoic	No effect	SB-4	No effect
	2-hydroxy-3,5-diiodobenzaldehyde	6.3 ± 0.0	3,4-diaminobenzoic	No effect	SB-5	8.6 ± 1.1
*Staphylococcus aureus* strain 7	3,5-dichlorosalicyaldehide	No effect	3,4-diaminobenzoic	No effect	SB-3	No effect
	3,5-dibromo-2-hydroxybenzaldehyde	12.6 ± 0.0	3,4-diaminobenzoic	No effect	SB-4	No effect
	2-hydroxy-3,5-diiodobenzaldehyde	6.3 ± 0.0	3,4-diaminobenzoic	No effect	SB-5	8.6 ± 1.1
*Staphylococcus haemolyticus*	3,5-dichlorosalicyaldehide	No effect	3,4-diaminobenzoic	No effect	SB-3	No effect
	3,5-dibromo-2-hydroxybenzaldehyde	No effect	3,4-diaminobenzoic	No effect	SB-4	No effect
	2-hydroxy-3,5-diiodobenzaldehyde	12.6 ± 0.0	3,4-diaminobenzoic	No effect	SB-5	14.1 ± 1.5

No effect: The treatment’s effect is not significantly different from that observed with vehicle control.

**Table 6:** Determination of the Minimal Inhibitory Concentration (MIC, µM) of aminobenzoic acid-derived Schiff Base (SB-3 to SB-5) of anaerobic, Gram-positive bacteria (*n* = 3).

Bacteria	SB-3	SB-4	SB-5
*Clostridioides difficile*	1.0 ± 0.0	1.0 ± 0.0	0.1 ± 0.0
*Blautia coccoides*	1.0 ± 0.0	1.0 ± 0.0	0.01 ± 0.0

## Data Availability

The original contributions presented in this study are included in the article/Supplementary Materials. Further inquiries can be directed to the corresponding authors. Supplementary data CCDC reference number 2389299 contains the supplementary crystallographic data for this paper. These data can be obtained free of charge at https://www.ccdc.cam.ac.uk/structures/search?access=referee&searchdepnums=2389299&searchauthor=Evys (accessed on 5 November 2025) [or from the Cambridge Crystallographic Data Centre, 12, Union Road, Cambridge CB2 1EZ, UK; Fax: (internat.) +44-1223/336-033; E-mail:deposit@ccdc.cam.ac.uk.

## Supplementary Materials

The following supporting information can be downloaded at: https://www.mdpi.com/article/10.3390/ijms262110801/s1.
