# Supplementary material for: Synthesis, Physicochemical Characterization, and Biocidal Evaluation of Three Novel Aminobenzoic Acid-Derived Schiff Bases Featuring Intramolecular Hydrogen Bonding
Source: Int J Mol Sci. 2025 Nov 6;26(21):10801. doi: 10.3390/ijms262110801 (PMC12608797; doi:10.3390/ijms262110801)
Supplement: Supplementary file 1 [file ijms-26-10801-s001.zip › ijms-3922605-supplementary.pdf]

Supplementary Materials

Synthesis, Physicochemical Characterization, and Biocidal Evaluation of Three Novel Aminobenzoic Acid-Derived Schiff Bases Featuring Intramolecular Hydrogen Bonding

Alexander Carreño [1,2,*], Vania Artigas [2], Belén Gómez-Arteaga [2], Evys Ancede-Gallardo [2], Marjorie Cepeda-Plaza [1], Jorge I. Martínez-Araya [1], Roxana Arce [1,3], Manuel Gacitúa [4], Camila Videla [2,5], Marcelo Preite [6], María Carolina Otero [5], Catalina Guerra [7], Rubén Polanco [7], Ignacio Fuentes [8,9], Pedro Marchant [8], Osvaldo Inostroza [10,11], Fernando Gil [10,11] and Juan A. Fuentes [8,*]

[1] Departamento de Ciencias Químicas, Facultad de Ciencias Exactas, Universidad Andres Bello, Av. República 275, Santiago 8370146, Chile; marjorie.cepeda@unab.cl (M.C.-P.); jorge.martinez@unab.cl (J.I.M.-A.); roxana.arce@unab.cl (R.A.)
[2] Laboratorio de Síntesis Organometálica, Centro de Nanociencias Aplicadas (CANS), Facultad de Ciencias Exactas, Universidad Andres Bello, Av. República 330, Santiago 8370186, Chile; vania.artigas@pucv.cl (V.A.); belen.gomez@usach.cl (B.G.-A.); eancedeg@gmail.com (E.A.-G.); camila.videla.e@gmail.com (C.V.)
[3] Millennium Institute on Green Ammonia as Energy Vector (MIGA), Av. Vicuña Mackenna 4860, Macul, Santiago 7820436, Chile
[4] Facultad de Ingeniería y Ciencias, Universidad Diego Portales, Ejército 441, Santiago 8370191, Chile; manuel.gacitua@mail.udp.cl
[5] Escuela de Química y Farmacia, Facultad de Medicina, Universidad Andres Bello, Sazié 2320, Santiago 7591538, Chile; maria.otero@unab.cl
[6] Departamento de Química Orgánica, Facultad de Química y de Farmacia, Pontificia Universidad Católica de Chile, Av. Vicuña Mackenna 4860, Santiago 7820436, Chile; mpreite@uc.cl
[7] Laboratorio de Hongos Fitopatógenos, Centro de Biotecnología Vegetal (CBV), Facultad de Ciencias de la Vida, Universidad Andres Bello, Av. República 330, Santiago 8370186, Chile; c.guerraramrez@uandresbello.edu (C.G.); rpolanco@unab.cl (R.P.)
[8] Laboratorio de Genética y Patogénesis Bacteriana, Centro de Investigación de Resiliencia a Pandemias,
Facultad de Ciencias de la Vida, Universidad Andres Bello, República 330, Santiago 8370186, Chile; ignaciofuentesc547@gmail.com (I.F.); marchant573@gmail.com (P.M.)
[9] Doctorado en Biotecnología, Facultad de Ciencias de la Vida, Universidad Andrés Bello, República 330, Santiago 8370186, Chile
[10] School of Medicine, Faculty of Medicine, Universidad de los Andes, Santiago 7620001, Chile; osv.inostroza.t@gmail.com (O.I.); frgil@uandes.cl (F.G.)
[11] Microbiota-Host Interactions & Clostridia Research Group, Center for Biomedical Research and Innovation (CIIB), Universidad de los Andes, Av. Monseñor Álvaro del Portillo 12455, Santiago 7620001, Chile
* Correspondence: alexander.carreno@unab.cl (A.C.); jfuentes@unab.cl (J.A.F.)

Supplementary Schemes

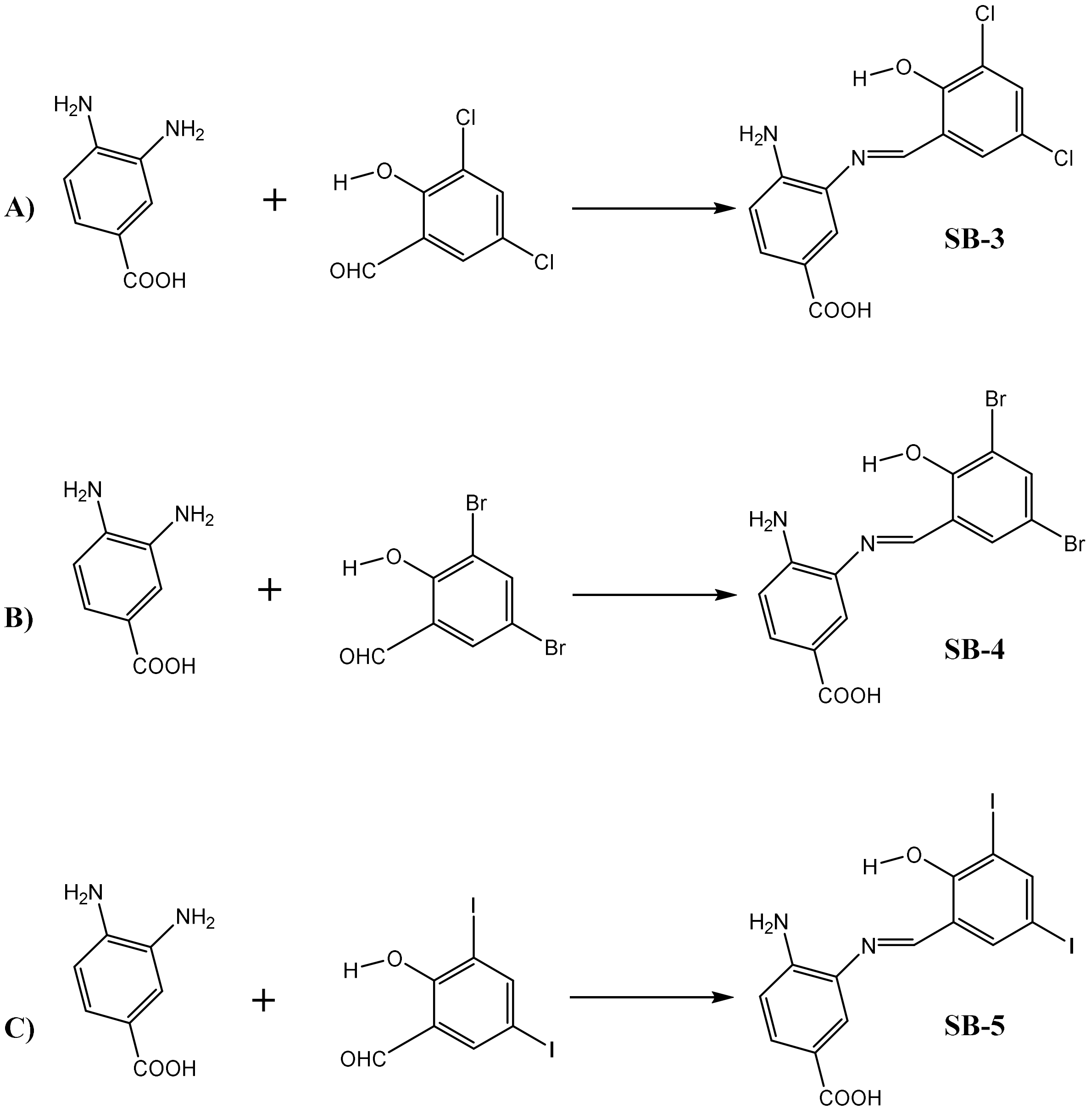

Scheme S1. Synthetic route of A) SB-3, B) SB-4, and C) SB-5 in methanol as solvent at room temperature.

Supplementary Table

Table S1. Characteristic constants of Metal-free Schiff bases SB-3, SB-4, and SB-5.

**Schiff base**	**M.F.**	**M.W. (g/mol)**	**Yield (%)**	**Solid color**	**Melting Points (°C)**
SB-3	$C_{14}H_{10}Cl_2N_2O_3$	325.15	78	light orange	242, dec.
SB-4	$C_{14}H_{10}Br_2N_2O_3$	414.05	80	light orange	237-238
SB-5	$C_{14}H_{10}I_2N_2O_3$	508.05	79	orange	175, dec.

M.F.: Molecular formula; **M.W.**: Molecular weight

Table S2. Selected proton assignments (in ppm) of SB-3 to SB-5 in deuterated DMSO.

**Proton**	**SB-3**	**SB-4**	**SB-5**
-OH	13.65 (s)	13.84 (s)	13.99 (s)
-COOH	12.30 (s)	12.33 (s)	12.33 (s)
$-NH_2$	6.01 (s)	6.00 (s)	5.98 (s)
H1	7.64 (d)	7.64 (d)	7.64 (d)
H2	6.80 (d)	6.81 (d)	6.80 (d)
H3	7.86 (s)	7.99 (s)	8.21-8.06 (m)
H4	8.97 (s)	8.94 (s)	8.85 (s)

For the arbitrary numbering of protons, see Figure S7.

(s): singlet; (d): doublet; (m): multiplet

Table S3. Selected carbon assignments (in ppm) of SB-3 to SB-5 in deuterated DMSO.

**Compound**	**carbonyl (-COOH) / ppm**	**azomethine (-N=C-)/ ppm**
**SB-3**	167.71	161.02
**SB-4**	168.02	161.32
**SB-5**	167.70	161.68

Table S4. Bond distances (Å) and angles (°) for SB-5.

**Bond distances (Å)**			
I(1)-C(2)	2.077(10)	C(6)-C(5)	1.394(13)
I(2)-C(4)	2.097(10)	C(6)-C(7)	1.429(14)
O(1)-C(1)	1.339(11)	C(1)-C(2)	1.401(13)
O(2)-C(14)	1.241(12)	C(13)-C(12)	1.386(13)
O(3)-C(14)	1.306(12)	C(2)-C(3)	1.365(13)
N(1)-C(8)	1.397(12)	C(9)-C(10)	1.416(15)
N(1)-C(7)	1.285(11)	C(3)-C(4)	1.385(14)
N(2)-C(9)	1.357(12)	C(14)-C(12)	1.455(14)
C(8)-C(13)	1.368(13)	C(5)-C(4)	1.394(14)
C(8)-C(9)	1.404(13)	C(12)-C(11)	1.393(14)
C(6)-C(1)	1.410(13)	C(11)-C(10)	1.337(14)
**Angles (°)**			
C(7)-N(1)-C(8)	124.4(8)	C(8)-C(9)-C(10)	118.3(9)
N(1)-C(8)-C(9)	116.1(9)	C(2)-C(3)-C(4)	119.5(10)
C(13)-C(8)-N(1)	124.1(9)	O(2)-C(14)-O(3)	122.6(10)
C(13)-C(8)-C(9)	119.8(9)	O(2)-C(14)-C(12)	122.1(10)
C(1)-C(6)-C(7)	120.4(9)	O(3)-C(14)-C(12)	115.3(9)
C(5)-C(6)-C(1)	119.1(9)	C(4)-C(5)-C(6)	119.7(10)
C(5)-C(6)-C(7)	120.4(9)	C(13)-C(12)-C(14)	120.5(9)
O(1)-C(1)-C(6)	120.2(9)	C(13)-C(12)-C(11)	117.2(9)
O(1)-C(1)-C(2)	120.2(9)	C(11)-C(12)-C(14)	122.3(9)
C(2)-C(1)-C(6)	119.5(9)	N(1)-C(7)-C(6)	122.1(9)
C(8)-C(13)-C(12)	121.9(9)	C(10)-C(11)-C(12)	123.1(10)
C(1)-C(2)-I(1)	118.7(7)	C(3)-C(4)-I(2)	119.0(7)
C(3)-C(2)-I(1)	120.1(8)	C(3)-C(4)-C(5)	121.1(9)
C(3)-C(2)-C(1)	121.1(9)	C(5)-C(4)-I(2)	119.8(8)
N(2)-C(9)-C(8)	121.9(9)	C(11)-C(10)-C(9)	119.7(10)
N(2)-C(9)-C(10)	119.8(9)		

Table S5. Global reactivity descriptors for SB-3, SB-4, and SB-5. All values are given in atomic units.

**Global Reactivity Descriptors**		**FDA**		
**Name**	**Symbol**	**SB-3**	**SB-4**	**SB-5**
First Vertical Ionization Potential	I_1	*0,280*	*0,279*	*0,276*
First Vertical Electron Affinity	A_1	*0,0426*	*0,0442*	*0,0448*
Electronic Chemical Potential	*m*	*-0,161*	*-0,162*	*-0,160*
Molecular Hardness	*h*	*0,238*	*0,235*	*0,231*
Global Softness	*S*	*4,211*	*4,254*	*4,332*
Electrophilicity	*w*	*0,0548*	*0,0556*	*0,0556*
Electron-donating Power	w^-	*0,205*	*0,207*	*0,206*
Electron-accepting Power	w^+	*0,0438*	*0,0451*	*0,0455*
Net Electrophilicity	D*w*	*0,249*	*0,252*	*0,251*

Global reactivity descriptors.

Several reactivity descriptors were proposed from the Conceptual Density Functional Theory. Those that do not depend upon position vector **r** are called global reactivity descriptors. From the so-called Canonical Ensemble, where the energy $\boldsymbol{E}$ is assumed as a function of $\boldsymbol{N}$, the number of electrons, and as a functional of the external potential, $\boldsymbol{v}(\mathbf{r})$ in the first place, we introduce the electronic chemical potential $\boldsymbol{\mu} = \left(\frac{\partial E}{\partial N}\right)_{v(\mathrm{r})} = -\boldsymbol{\chi}$, which is expressed in $\boldsymbol{hartree} \cdot \boldsymbol{e^{-1}}$,and $\boldsymbol{\chi}$ is the electronegativity according to the Mülliken's definition.

It measures the escaping tendency of electrons from equilibrium, and its working formula, based on the finite difference approximation (FDA), is $\boldsymbol{\mu} = -\mathbf{0.5}(\boldsymbol{I_1} + \boldsymbol{A_1})$ where I_1 is the first vertical ionization potential, and A_1 is the first vertical electron affinity.

The molecular hardness, $\boldsymbol{\eta} = \left(\frac{\partial^2 E}{\partial N^2}\right)_{v(\mathrm{r})}$ is expressed in $\boldsymbol{hartree} \cdot \boldsymbol{e^{-2}}$ and it is understood as the resistance to charge transfer; its working formula based on the FDA is $\boldsymbol{\eta} = \boldsymbol{I_1} - \boldsymbol{A_1}$. The global softness is defined as $\boldsymbol{S} = \boldsymbol{\eta^{-1}} = \left(\frac{\partial^2 E}{\partial N^2}\right)^{-1}_{v(r)}$ and it is expressed in $\boldsymbol{hartree^{-1}} \cdot \boldsymbol{e^{2}}$; it quantifies the ease of charge transfer of a system, its working formula, based on the FDA, is $\boldsymbol{S} = (\boldsymbol{I_1} - \boldsymbol{A_1})^{-1}$.

The molecular hyper-hardness, $\boldsymbol{\gamma} = \left(\frac{\partial^3 E}{\partial N^3}\right)_{v(\mathrm{r})}$ is expressed in $\boldsymbol{hartree} \cdot \boldsymbol{e^{-3}}$ and whose physical meaning and relevance are under study. Even so, its working formula based on the FDA is given by $\boldsymbol{\gamma} = \mathbf{0.5}(\boldsymbol{I_1} + \boldsymbol{A_1} - \boldsymbol{I_2} - \boldsymbol{A_2})$ and usually its absolute value is smaller than that of $\boldsymbol{\mu}$ and $\boldsymbol{\eta}$. In order to take into account the electronic chemical potential and the molecular hardness at the same time, the electrophilicity index $\boldsymbol{\omega}$ was proposed as $\boldsymbol{\omega} = \frac{\mu^2}{2\eta}$.

It measures the energy change of an electrophile when it becomes saturated with electrons, by considering the case in which an electrophilic species is immersed in an idealized, zero-temperature free-electron sea of zero chemical potential. Its working formula corresponds to $\boldsymbol{\omega} = \frac{(I_1+A_1)^2}{8(I_1-A_1)}$ which is based on the FDA. To quantify the response to charge donation and charge acceptance, the electron-donating and electron-accepting powers are defined as follows, respectively: $\boldsymbol{\omega^-} = \frac{(\mu^-)^2}{2\eta^-}$ and $\boldsymbol{\omega^+} =$

$\frac{(\mu^+)^2}{2\eta^+}$. They work as follows: a smaller value of $\boldsymbol{\omega}^-$ of a system makes it a better electron donor, whereas a larger $\boldsymbol{\omega}^+$ value corresponds to a greater ability to accept charge. Working formulae are defined as follows $\boldsymbol{\omega}^- = \frac{(3I_1+A_1)^2}{16(I_1-A_1)}$ and $\boldsymbol{\omega}^+ = \frac{(I_1+3A_1)^2}{16(I_1-A_1)}$ which are based on the FDA. In order to quantify these capabilities in just one term, the net electrophilicity $\boldsymbol{\Delta\omega}^\pm$ was proposed, and it corresponds to an electron-accepting power relative to electron-donating power; its working formula is given by: $\boldsymbol{\Delta\omega}^\pm = \boldsymbol{\omega}^+ - (-\boldsymbol{\omega}^-) = \boldsymbol{\omega}^+ + \boldsymbol{\omega}^-$.

Local reactivity: Local Hyper-softness

From the Conceptual Density Functional Theory, a local reactivity descriptor that reveals sites on a molecule that are susceptible to undergo nucleophilic and electrophilic attacks, trending to form covalent bonds, is the Fukui function, which presents two forms: the nucleophilic Fukui function $f^+(\mathbf{r})$ (to quantify nucleophilic attacks) and the electrophilic Fukui function $f^-(\mathbf{r})$ (to quantify electrophilic attacks). They are scalar fields ranging from 0 to 1, and, under the FDA, they are written in terms of electron densities:

$$\boldsymbol{f}^+(\mathbf{r}) = \boldsymbol{\rho}_{\boldsymbol{N+1}}(\mathbf{r}) - \boldsymbol{\rho}_{\boldsymbol{N}}(\mathbf{r})$$

$$\boldsymbol{f}^-(\mathbf{r}) = \boldsymbol{\rho}_{\boldsymbol{N}}(\mathbf{r}) - \boldsymbol{\rho}_{\boldsymbol{N-1}}(\mathbf{r})$$

These parameters can be plotted as 3D isosurfaces revealing electrophilic and nucleophilic regions on a molecule, respectively; its unit is $bohr^{-3}$.

To avoid using these two Fukui functions separately, a second-order Fukui function, also called a dual descriptor, has been defined by Christophe Morell et al. Its working formula is presented as follows:

$$\boldsymbol{f}^{(2)}(\mathbf{r}) = \boldsymbol{\rho}_{\boldsymbol{N+1}}(\mathbf{r}) - 2\boldsymbol{\rho}_{\boldsymbol{N}}(\mathbf{r}) + \boldsymbol{\rho}_{\boldsymbol{N-1}}(\mathbf{r})$$

Its unit is $e^{-1} \cdot bohr^{-3}$ where e means electron. It ranges from -1 to 1.

Nevertheless, neither the dual descriptor nor the Fukui functions can be used to compare local reactivities among different molecules because lobes become insignificant as the molecule's size increases.

The local hyper-softness (LHS) mends this intrinsic behavior of the dual descriptor and Fukui functions; its advantages are explained in J. Math. Chem. 62, 461–475 (2024).**[40]** LHS is a local reactivity descriptor that considers the molecular size, and whose working formula, based on the FMOA, is presented as follows:

$$s^{(2)}(\mathrm{r}) \approx S^2 f^{(2)}(\mathrm{r})$$

Where S^2 is the squared global softness. $s^{(2)}(\mathbf{r})$. Its unit is $e^3 \cdot hartree^{-2} \cdot bohr^{-3}$. This descriptor's advantage is that it allows comparison of local reactivity among different molecules; hence, we used it in the present work to assess the local reactivity of the molecules under analysis.

3D maps of local hyper-softness

The most accurate way to compute any reactivity descriptor of the Conceptual DFT implies using the finite difference approximation (FDA) because of the discrete nature of $\boldsymbol{N}$, the number of electrons.

FDA to obtain a 3D picture of LHS

$$\left(\frac{\partial^2 E}{\partial N^2}\right)^{-1}_{v(\mathrm{r})} = \boldsymbol{S} = (\boldsymbol{I_1} - \boldsymbol{A_1})^{-1}$$

Where $\boldsymbol{I_1} = \boldsymbol{E}(\boldsymbol{N}-\mathbf{1}) - \boldsymbol{E}(\boldsymbol{N})$ and $\boldsymbol{I_2} = \boldsymbol{E}(\boldsymbol{N}-\mathbf{2}) - \boldsymbol{E}(\boldsymbol{N}-\mathbf{1})$ are the first and second vertical ionization potentials. While $\boldsymbol{A_1} = \boldsymbol{E}(\boldsymbol{N}) - \boldsymbol{E}(\boldsymbol{N}+\mathbf{1})$ and $\boldsymbol{A_2} = \boldsymbol{E}(\boldsymbol{N}+\mathbf{1}) - \boldsymbol{E}(\boldsymbol{N}+\mathbf{2})$ are the first and second vertical electron affinities.

$\boldsymbol{E}(\boldsymbol{N}-\mathbf{2})$, $\boldsymbol{E}(\boldsymbol{N}-\mathbf{1})$, $\boldsymbol{E}(\boldsymbol{N})$, $\boldsymbol{E}(\boldsymbol{N}+\mathbf{1})$, and $\boldsymbol{E}(\boldsymbol{N}+\mathbf{2})$ correspond to the total energy of the system with $\boldsymbol{N}-\mathbf{2}$, $\boldsymbol{N}-\mathbf{1}$, $\boldsymbol{N}$, $\boldsymbol{N}+\mathbf{1}$, and $\boldsymbol{N}+\mathbf{2}$ electrons, respectively, computed with the molecular structure optimized with $\boldsymbol{N}$ electrons.

$$\left(\frac{\partial \boldsymbol{\rho}(\mathbf{r})}{\partial \boldsymbol{N}}\right)_{v(\mathrm{r})} = \boldsymbol{f}(\mathbf{r}) = \mathbf{0.5}\big(\boldsymbol{\rho}_{N+1}(\mathbf{r}) - \boldsymbol{\rho}_{N-1}(\mathbf{r})\big)$$

$$\left(\frac{\partial^2 \rho(\mathrm{r})}{\partial N^2}\right)_{v(\mathrm{r})} = \boldsymbol{f}^{(2)}(\mathbf{r}) = \boldsymbol{\rho}_{N+1}(\mathbf{r}) - \mathbf{2}\boldsymbol{\rho}_{N}(\mathbf{r}) + \boldsymbol{\rho}_{N-1}(\mathbf{r})$$

$$\left(\frac{\partial^3 E}{\partial N^3}\right)_{v(\mathrm{r})} = \boldsymbol{\gamma} = \mathbf{0.5}(\boldsymbol{I_1} + \boldsymbol{A_1} - \boldsymbol{I_2} - \boldsymbol{A_2})$$

$\rho_{N+1}(\mathrm{r})$, $\rho_N(\mathrm{r})$, and $\rho_{N-1}(\mathrm{r})$ stand for the electron densities for the system bearing $N+1$, N, and $N-1$ electrons.

All these working formulae lead to the working formula of LHS.

$$\left(\frac{\partial^2 \boldsymbol{\rho}(\mathbf{r})}{\partial \boldsymbol{\mu}^2}\right)_{\boldsymbol{v}(\mathbf{r})} = \boldsymbol{s}^{(2)}(\mathbf{r}) = \boldsymbol{S}^2 \boldsymbol{f}^{(2)}(\mathbf{r}) - \boldsymbol{S}^3 \boldsymbol{\gamma} \boldsymbol{f}(\mathbf{r})$$

More details on the working formula for the LHS are available in the referenced work [40].

Supplementary Figures

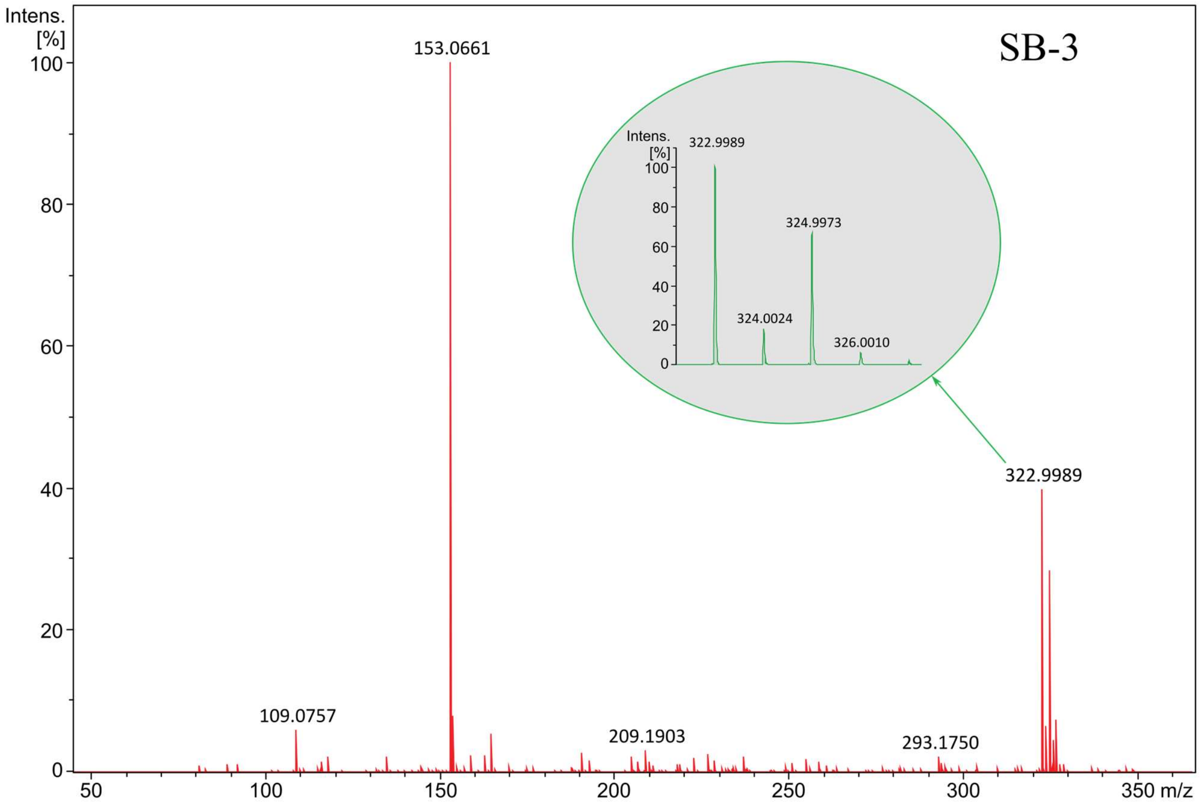

Figure S1. HRMS of SB-3.

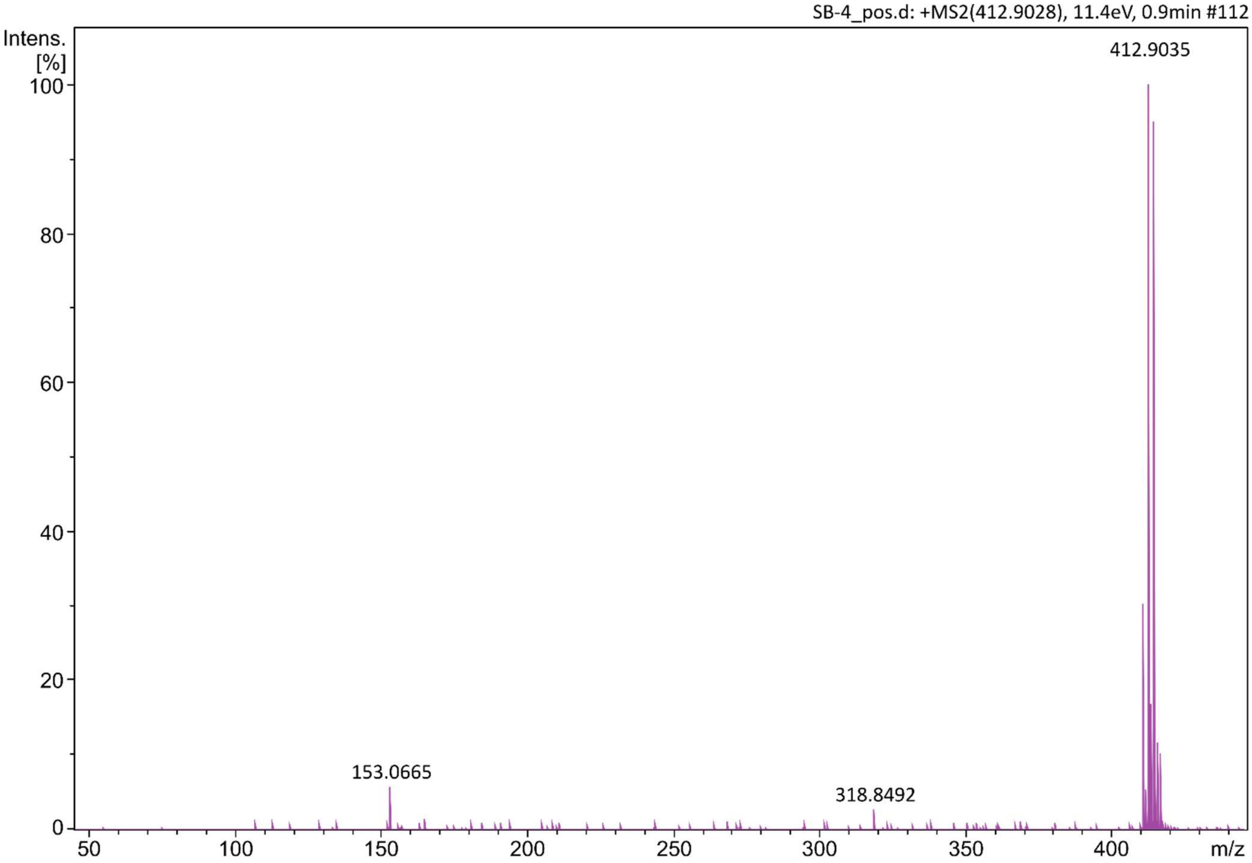

Figure S2. HRMS of SB-4.

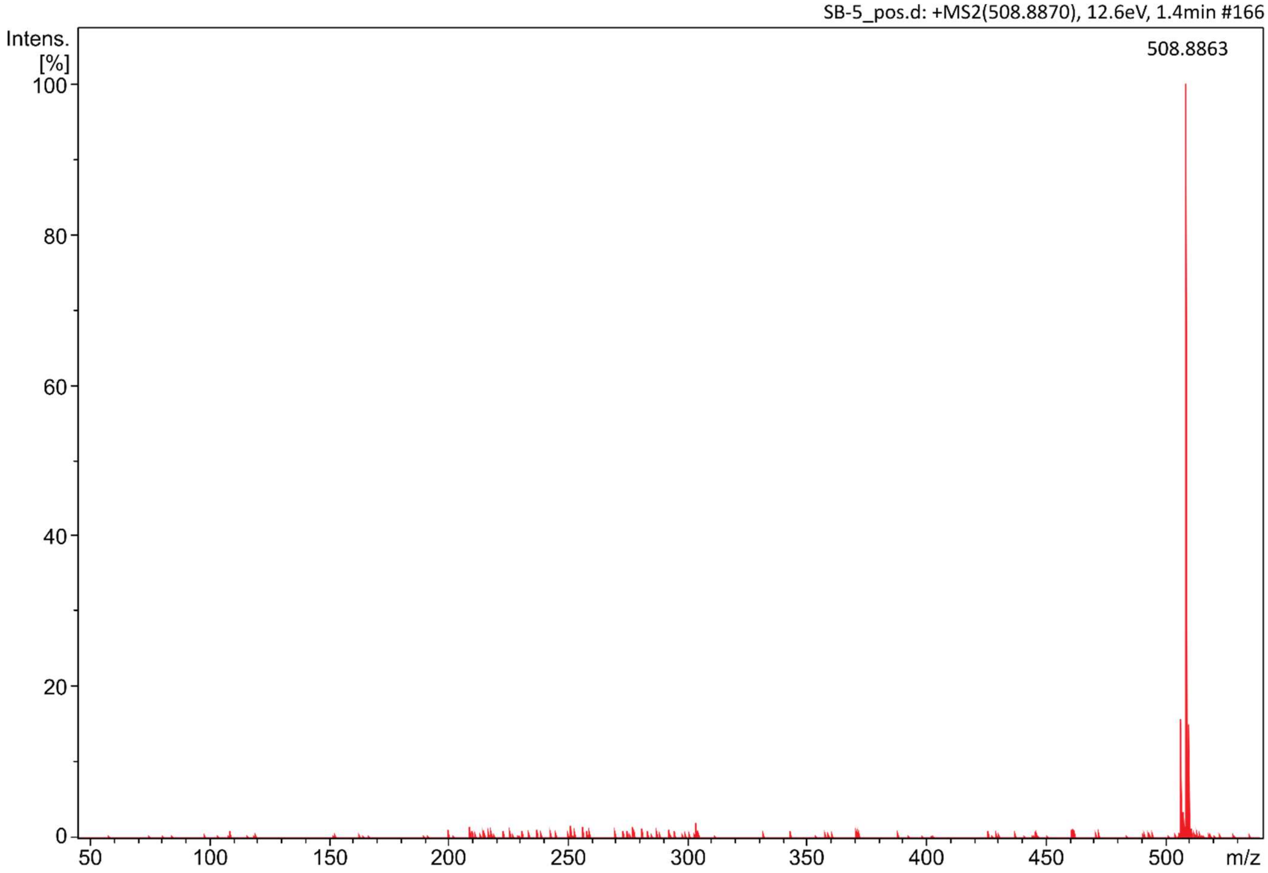

Figure S3. HRMS of SB-5.

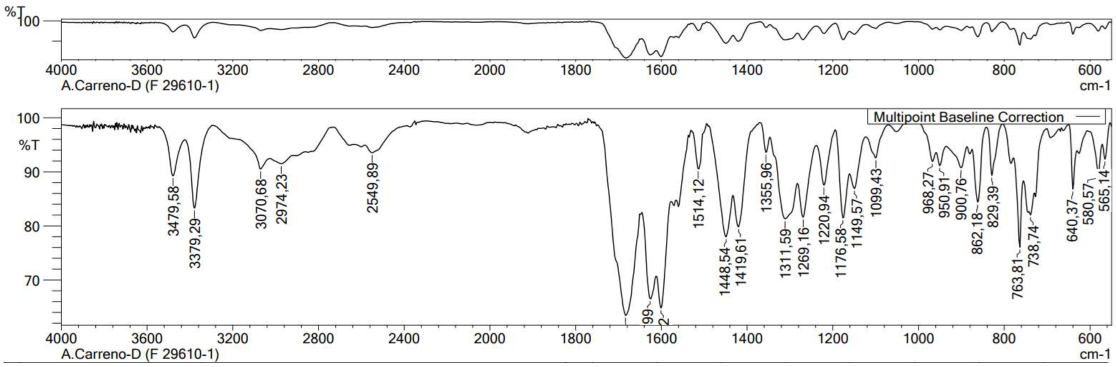

Figure S4. FTIR of SB-3.

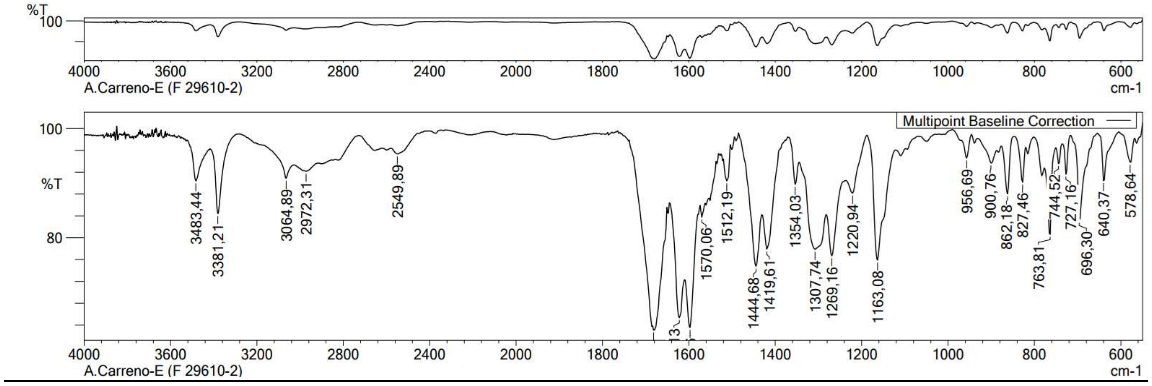

Figure S5. FTIR of SB-4.

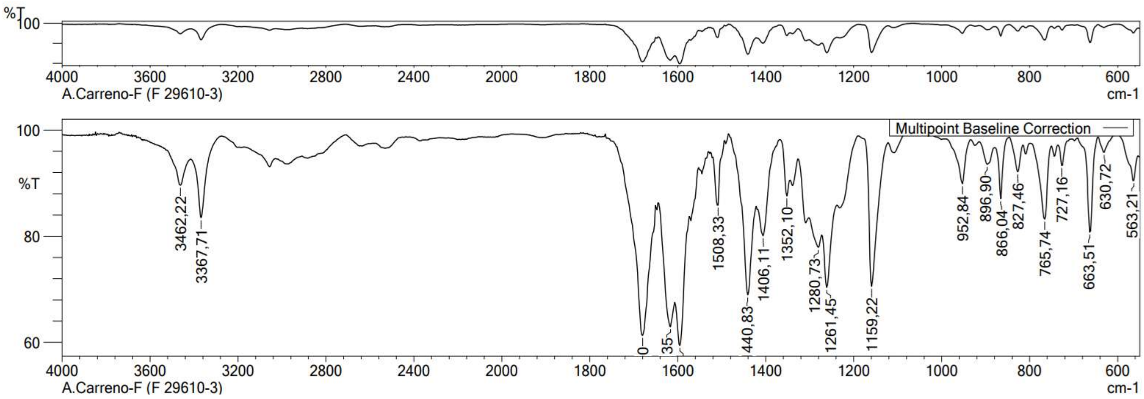

Figure S6. FTIR of SB-5.

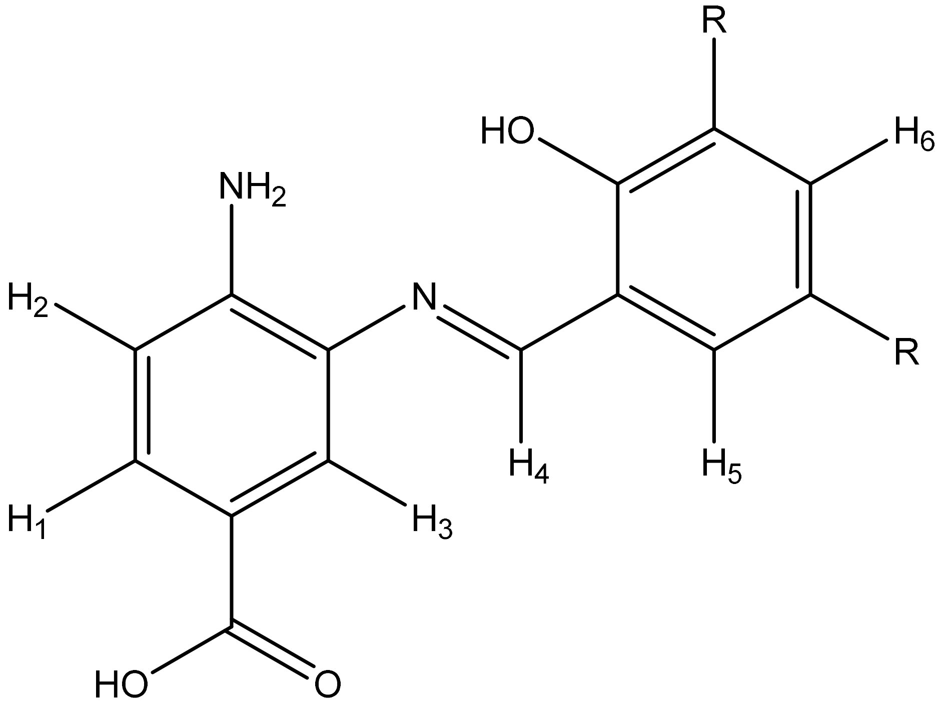

Figure S7. Arbitrary numbering of protons for SB-3, SB-4, and SB-5.

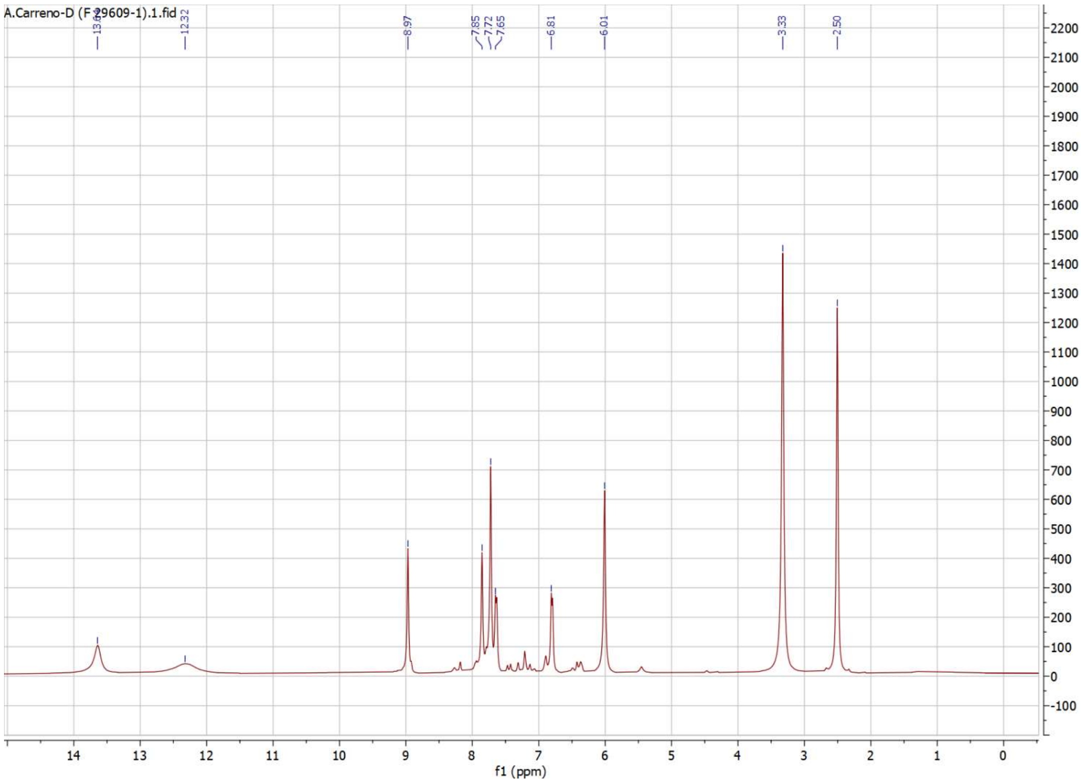

Figure S8. ^{1}H-NMR spectrum of SB-3 in deuterated DMSO.

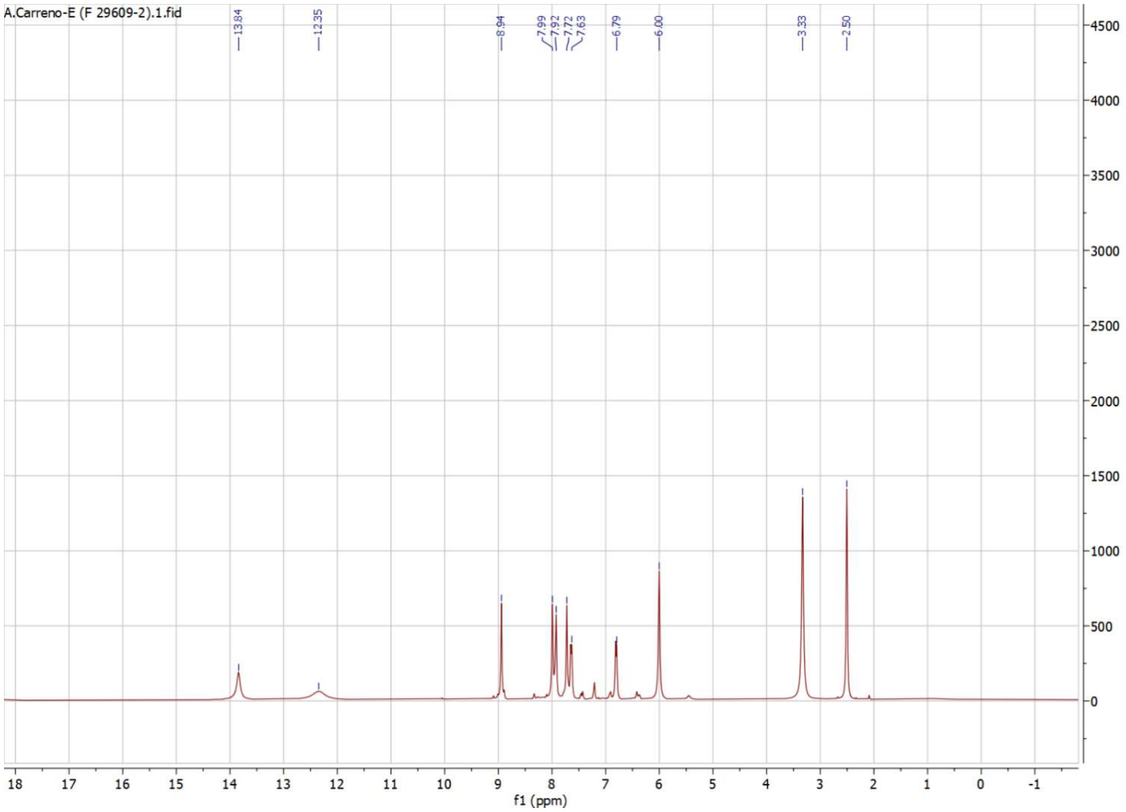

Figure S9. ^{1}H-NMR spectrum of SB-4 in deuterated DMSO.

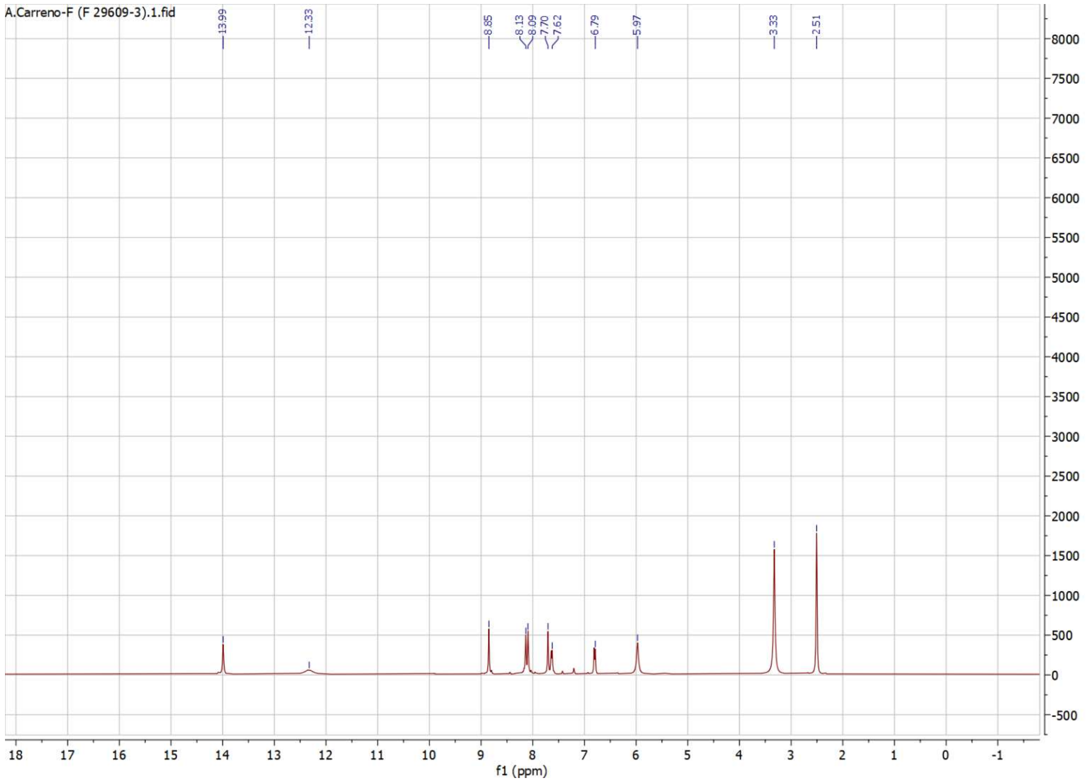

Figure S10. ¹H-NMR spectrum of SB-5 in deuterated DMSO.

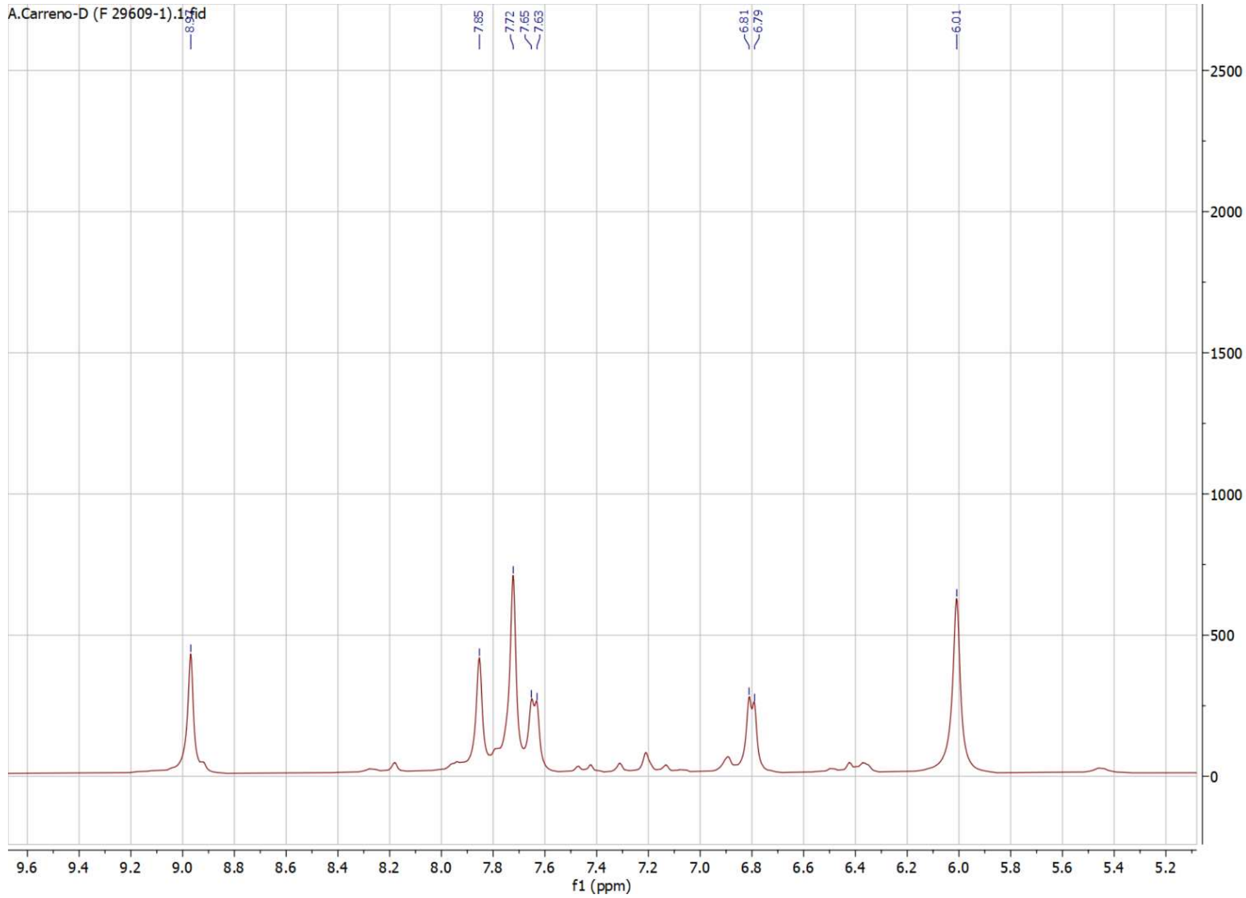

Figure S11. Expanded aromatic ^{1}H-NMR spectrum of SB-3 in deuterated DMSO.

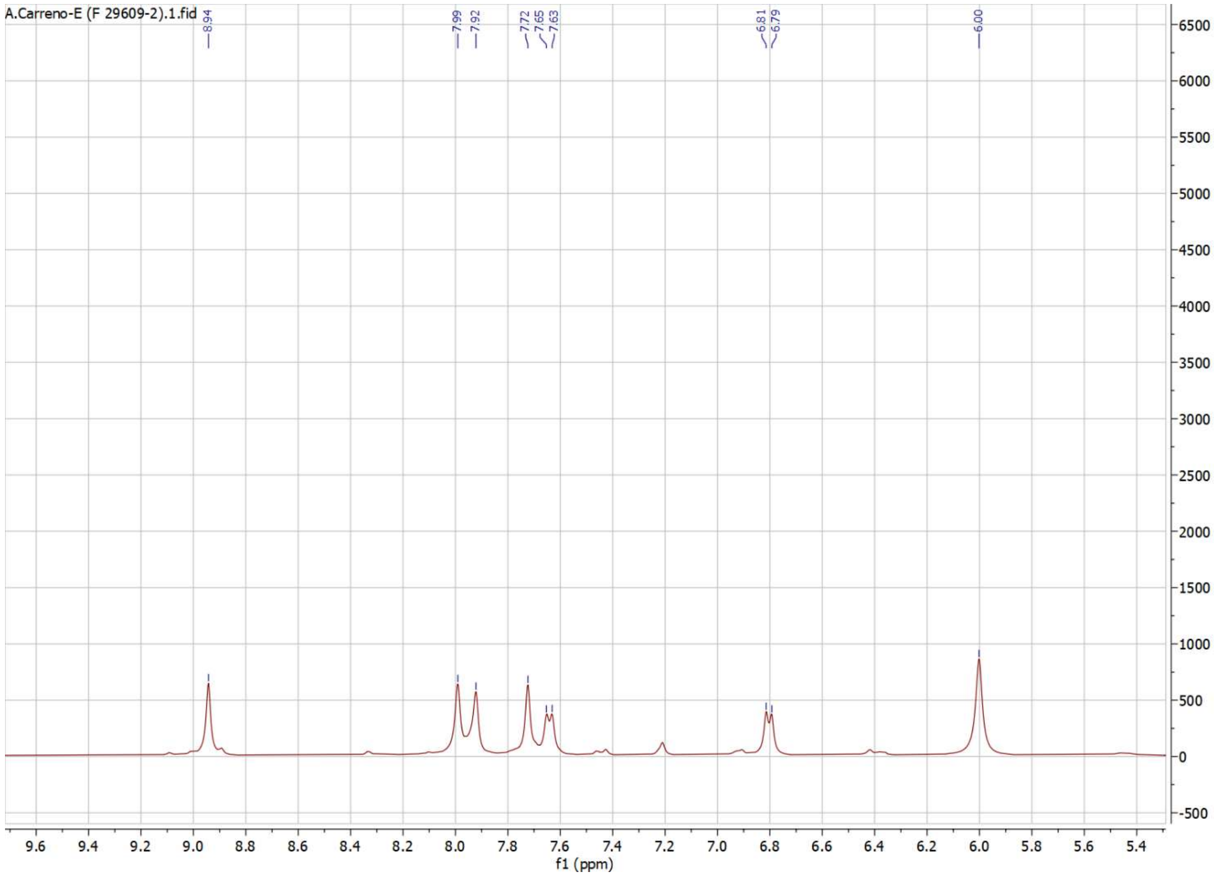

Figure S12. Expanded aromatic ^{1}H-NMR spectrum of SB-4 in deuterated DMSO.

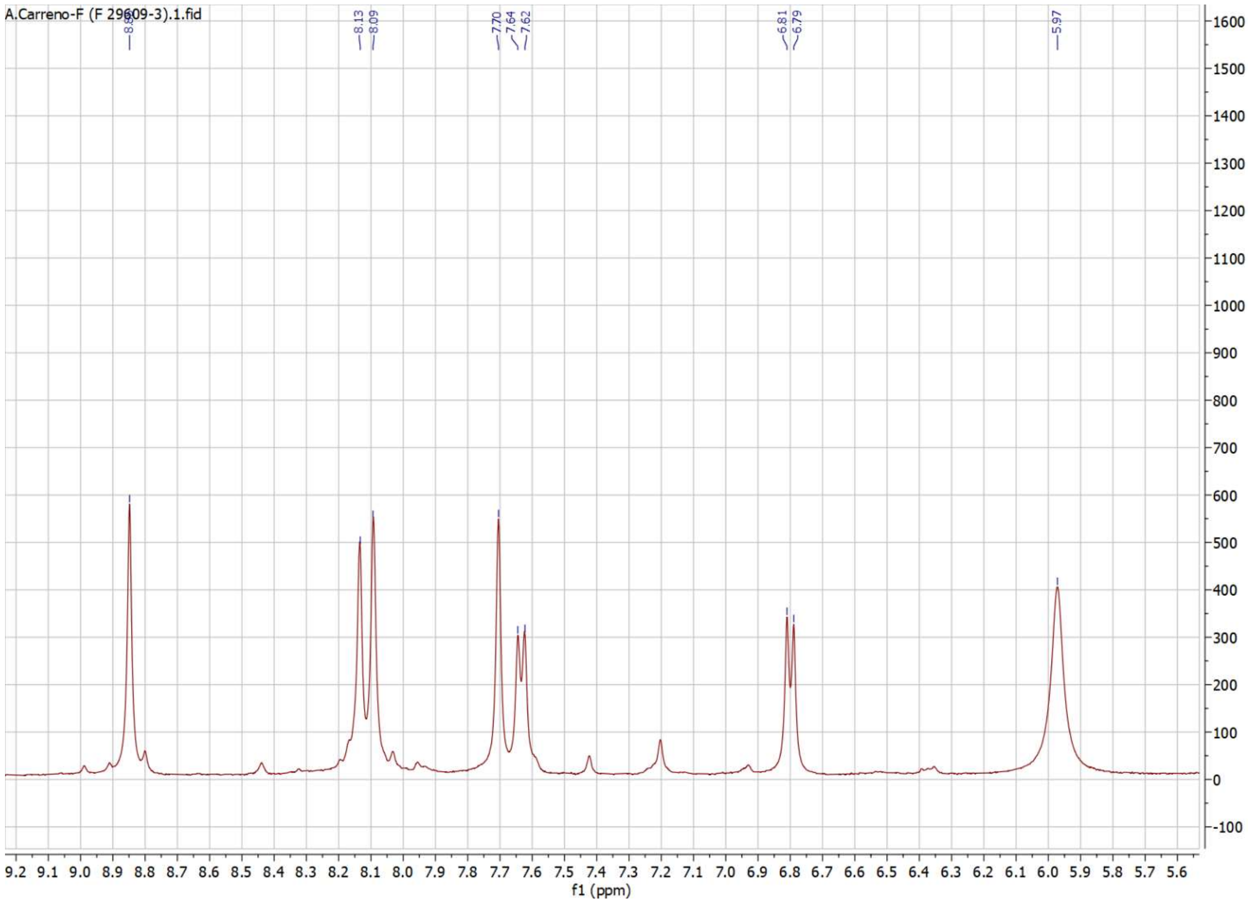

Figure S13. Expanded aromatic ^{1}H-NMR spectrum of SB-5 in deuterated DMSO.

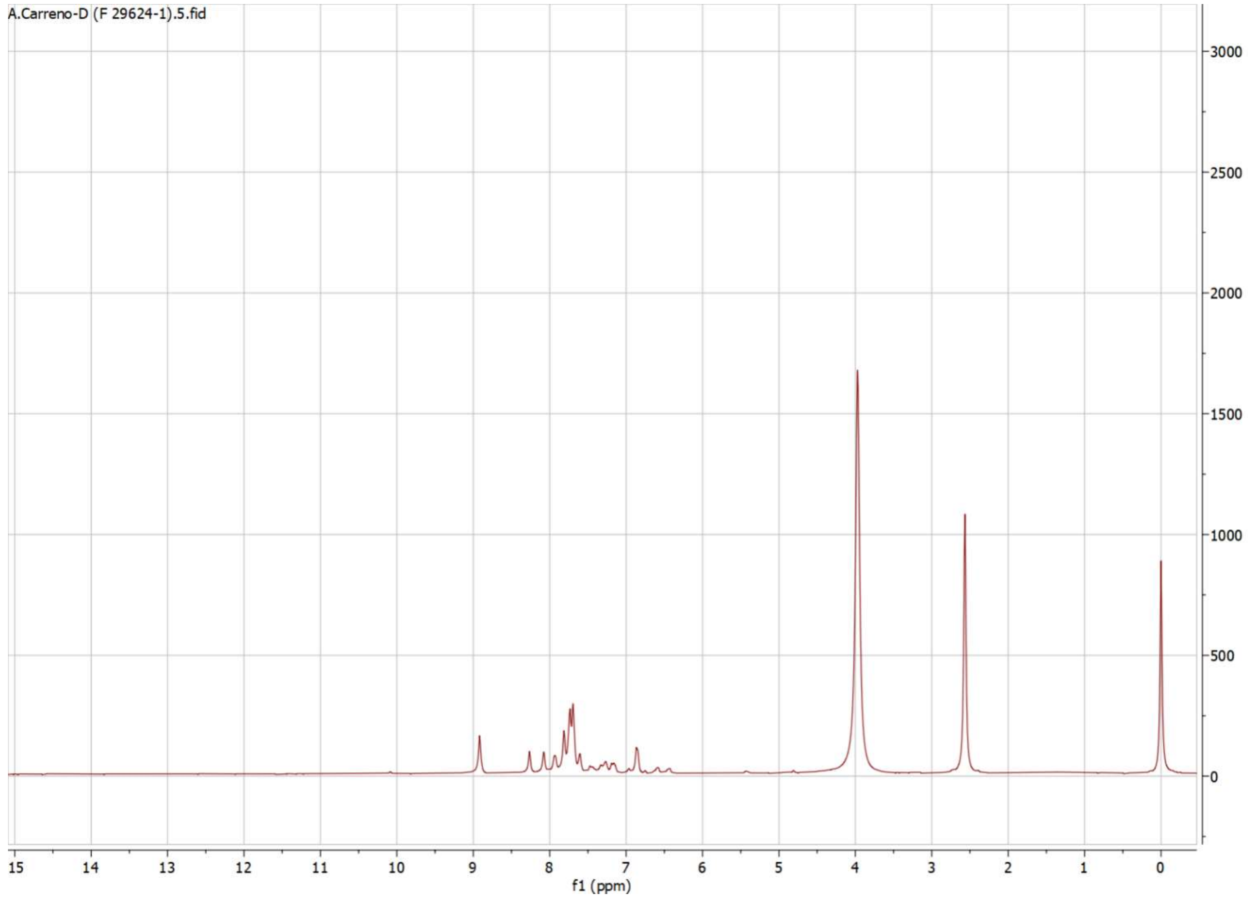

Figure S14. D_2O exchange of SB-3 in deuterated DMSO.

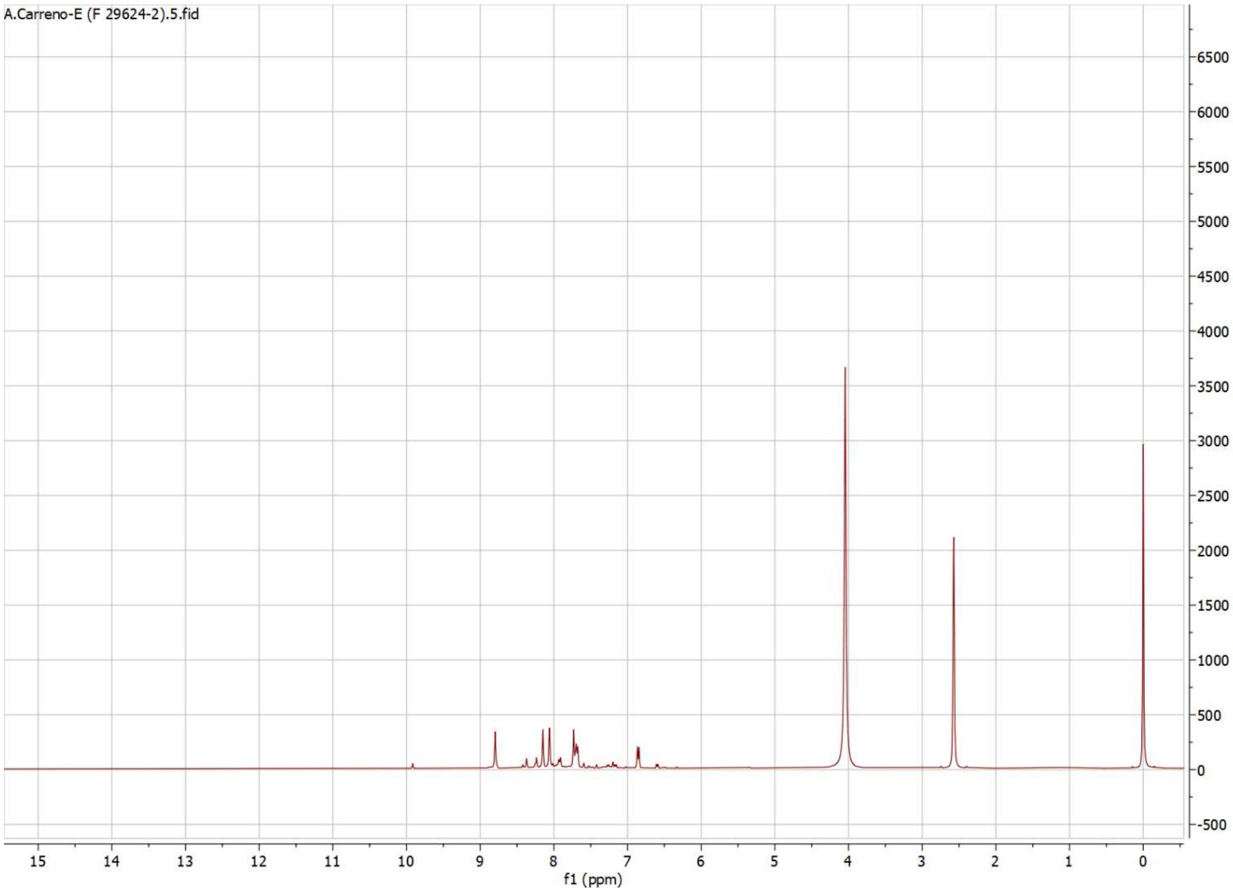

Figure S15. D_2O exchange of SB-4 in deuterated DMSO.

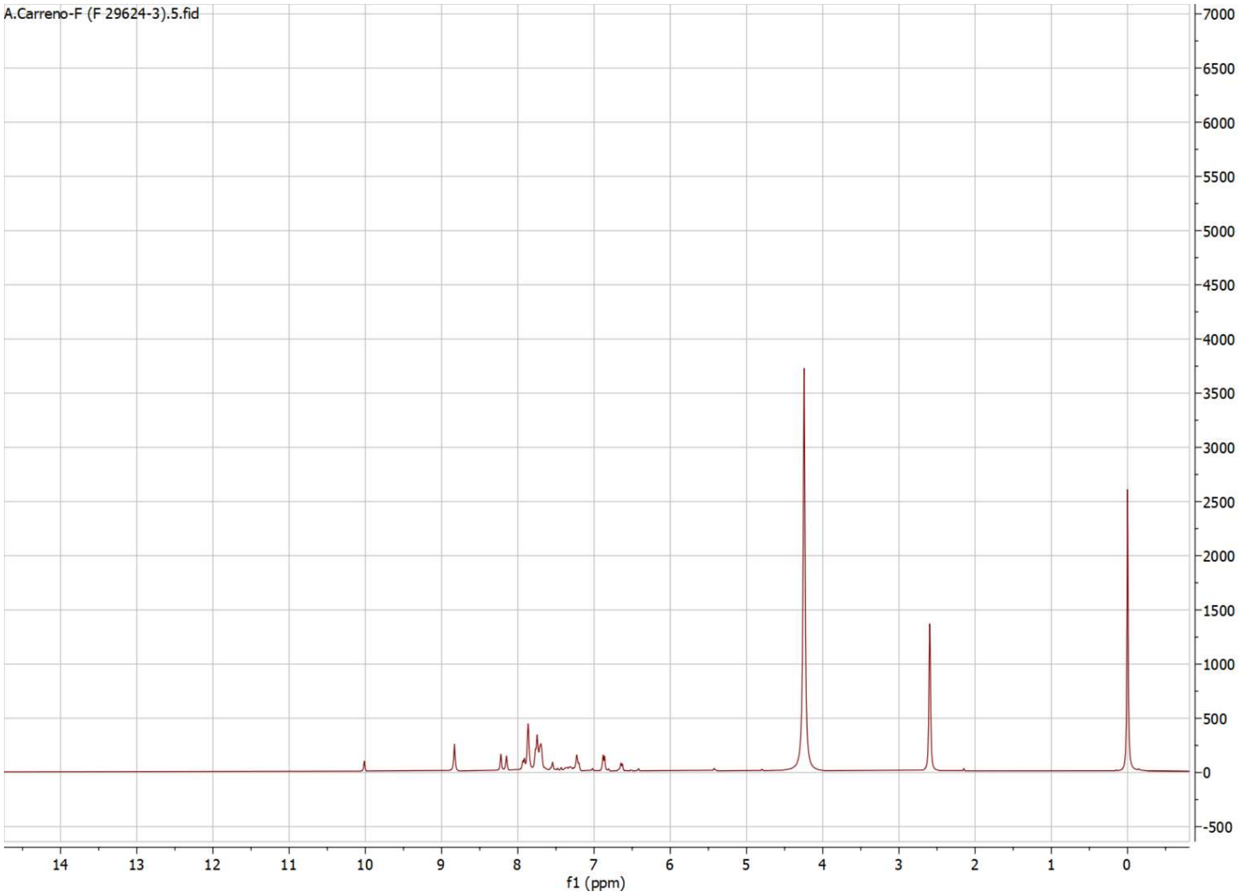

Figure S16. D_2O exchange of SB-5 in deuterated DMSO.

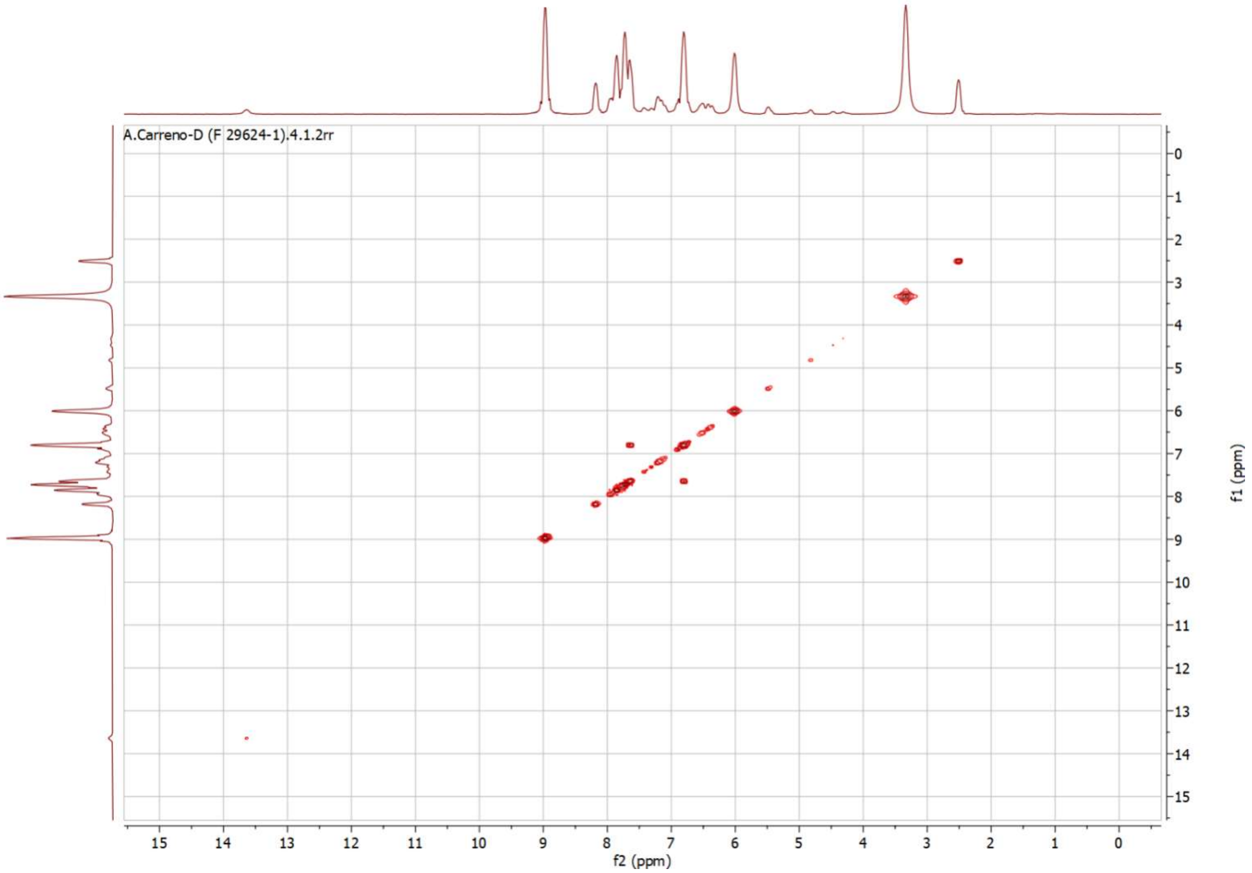

Figure S17. HHCOSY of SB-3 in deuterated DMSO.

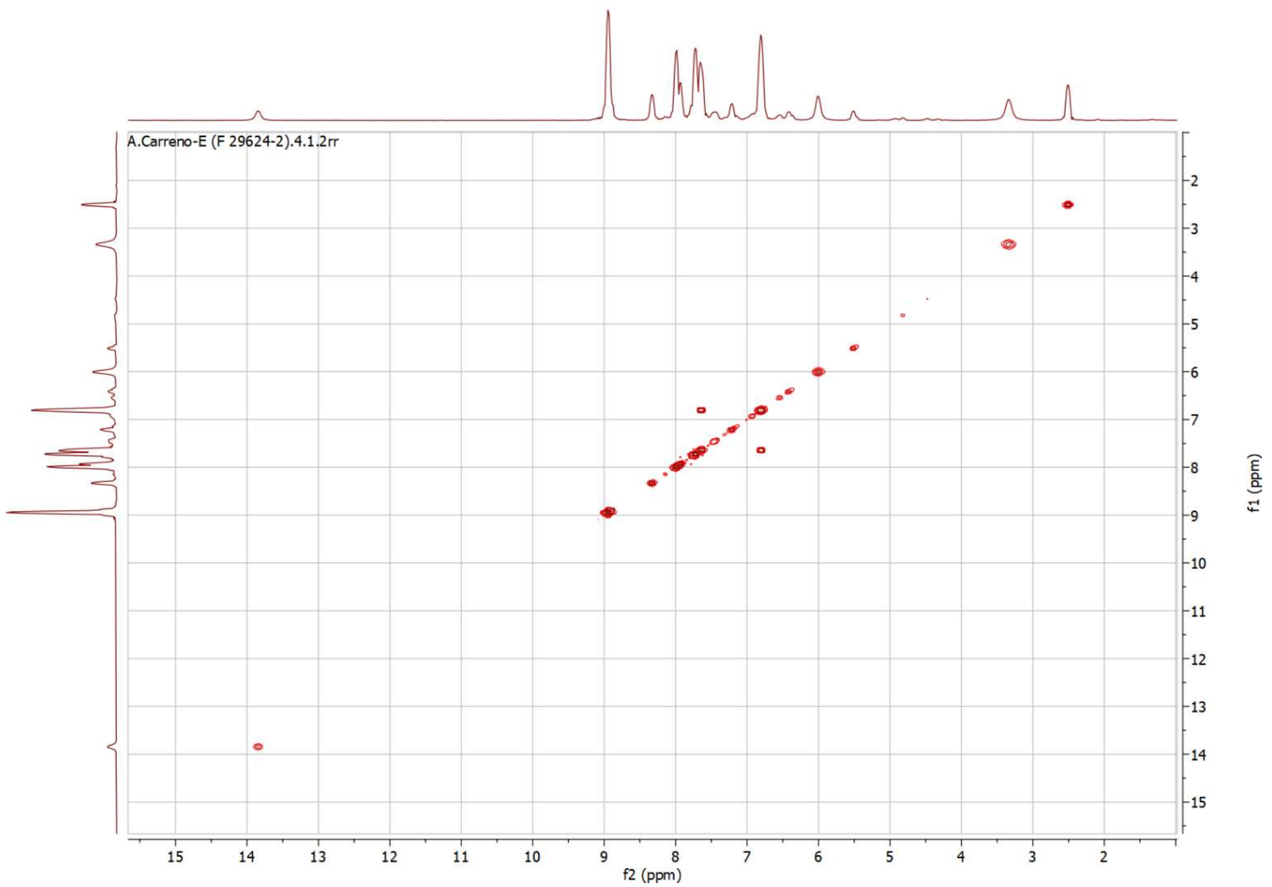

Figure S18. HHCOSY of SB-4 in deuterated DMSO.

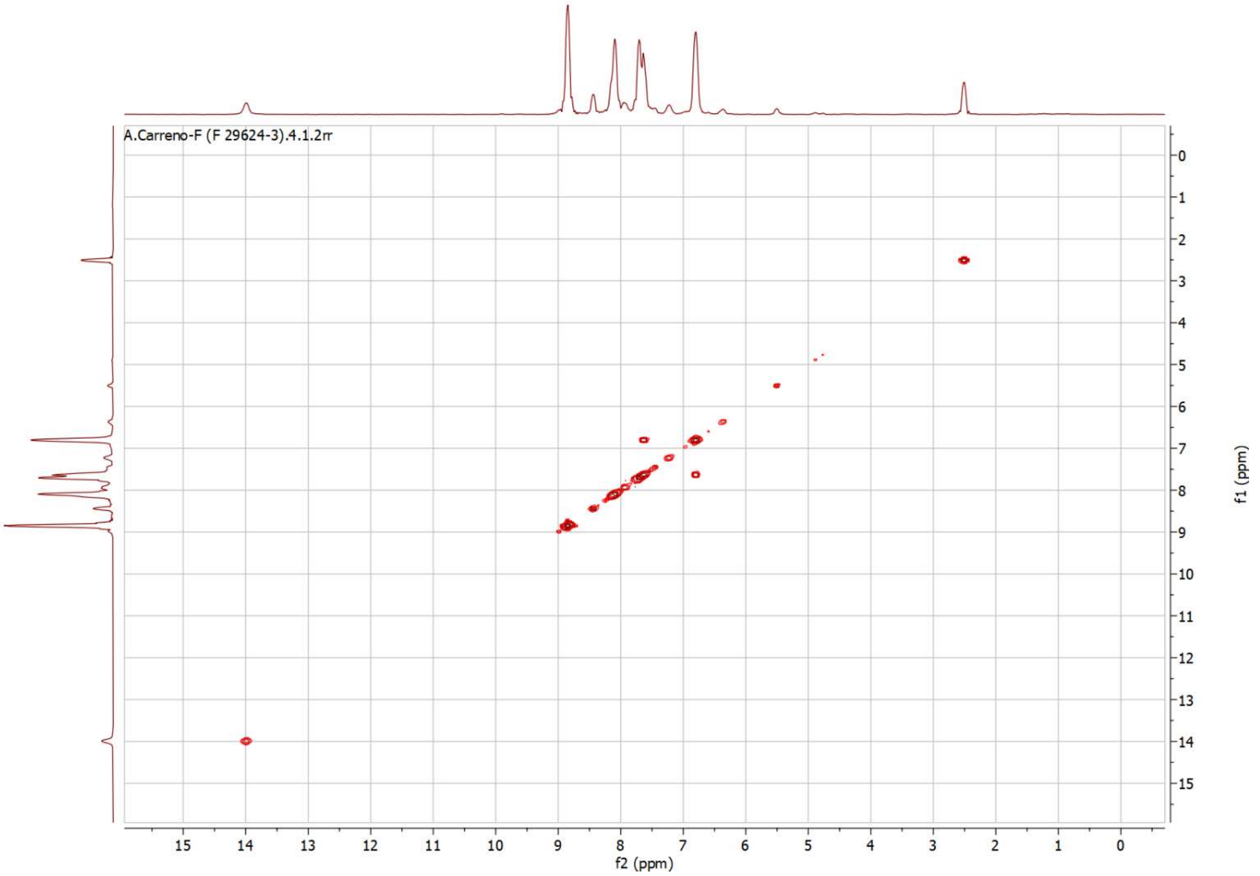

Figure S19. HHCOSY of SB-5 in deuterated DMSO.

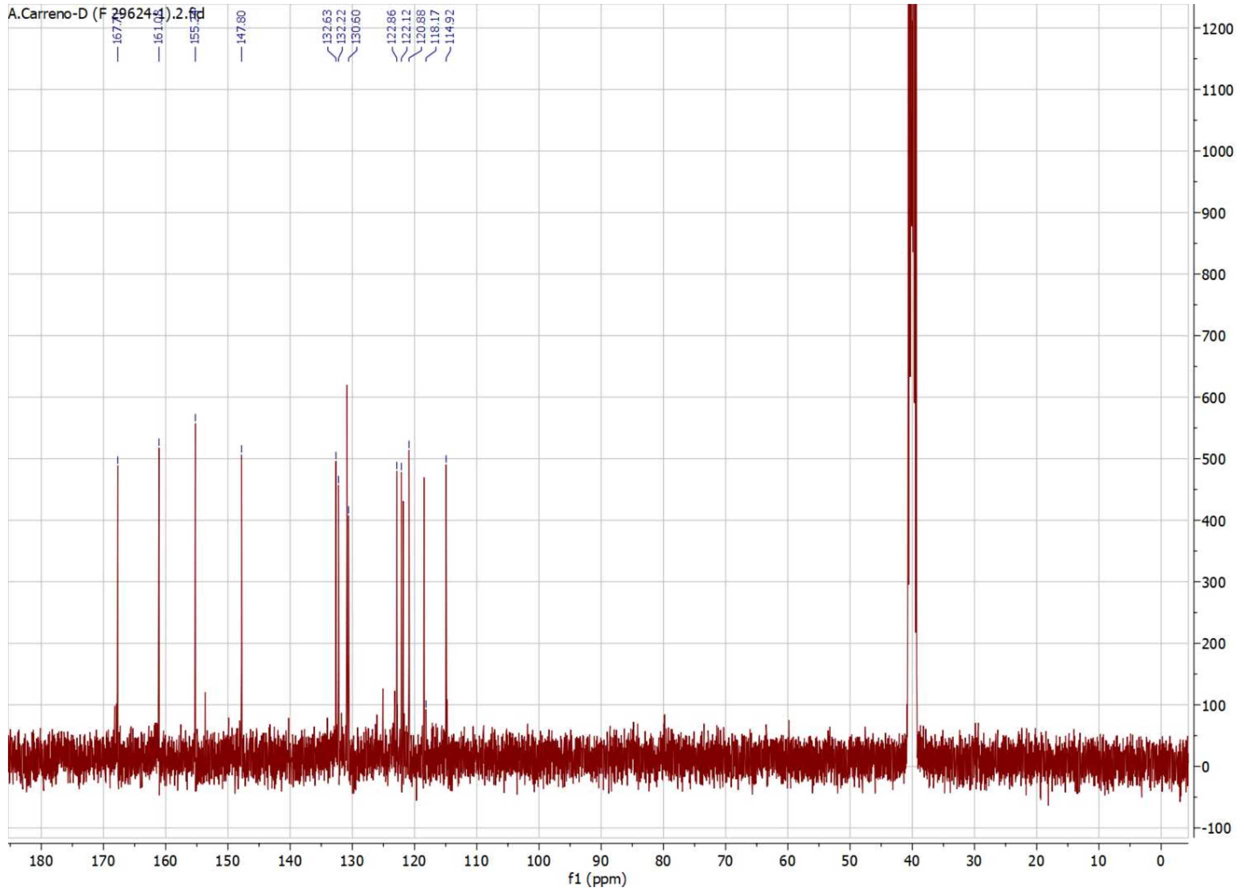

Figure S20. ^{13}C-NMR of SB-3 in deuterated DMSO.

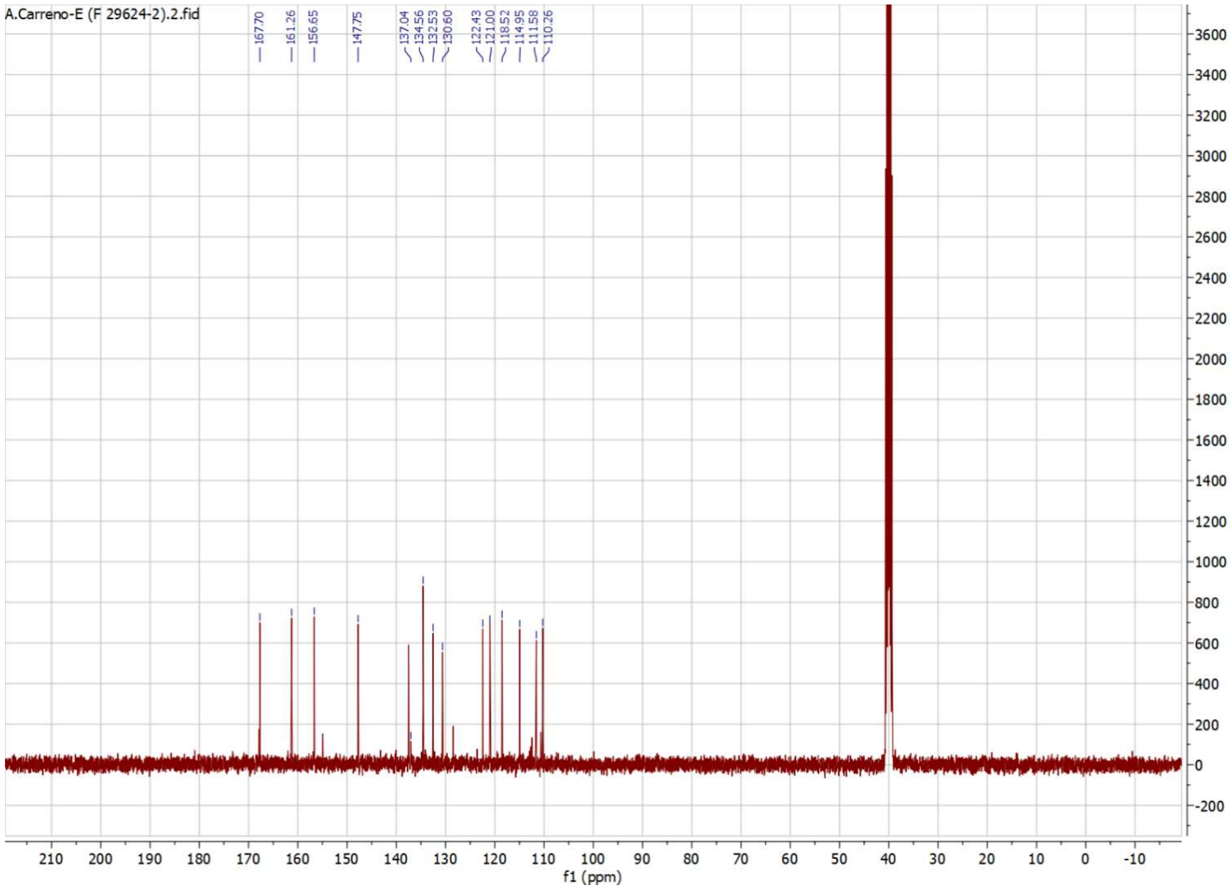

Figure S21. ^{13}C-NMR of SB-4 in deuterated DMSO.

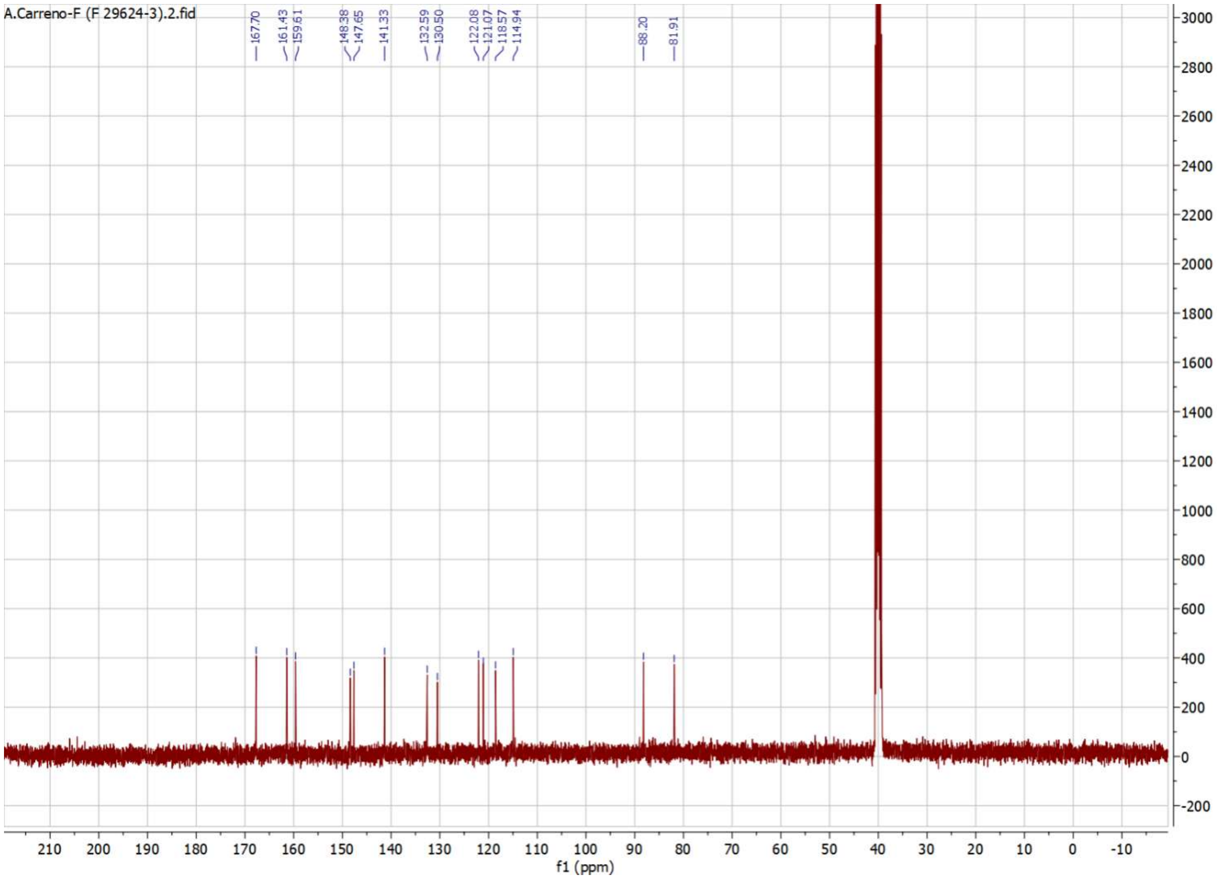

Figure S22. ^{13}C-NMR of SB-5 in deuterated DMSO.

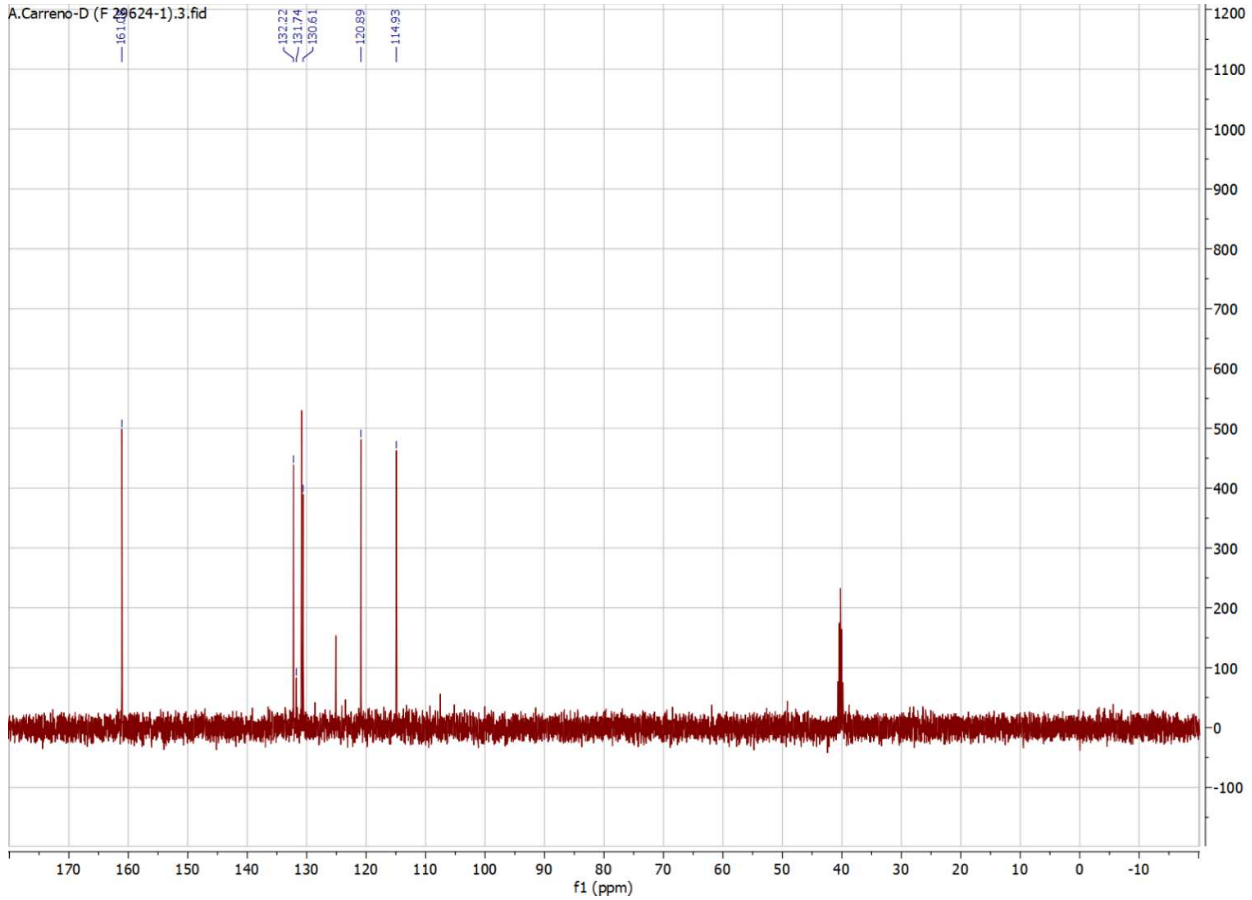

Figure S23. DEPT-45 of SB-3 in deuterated DMSO.

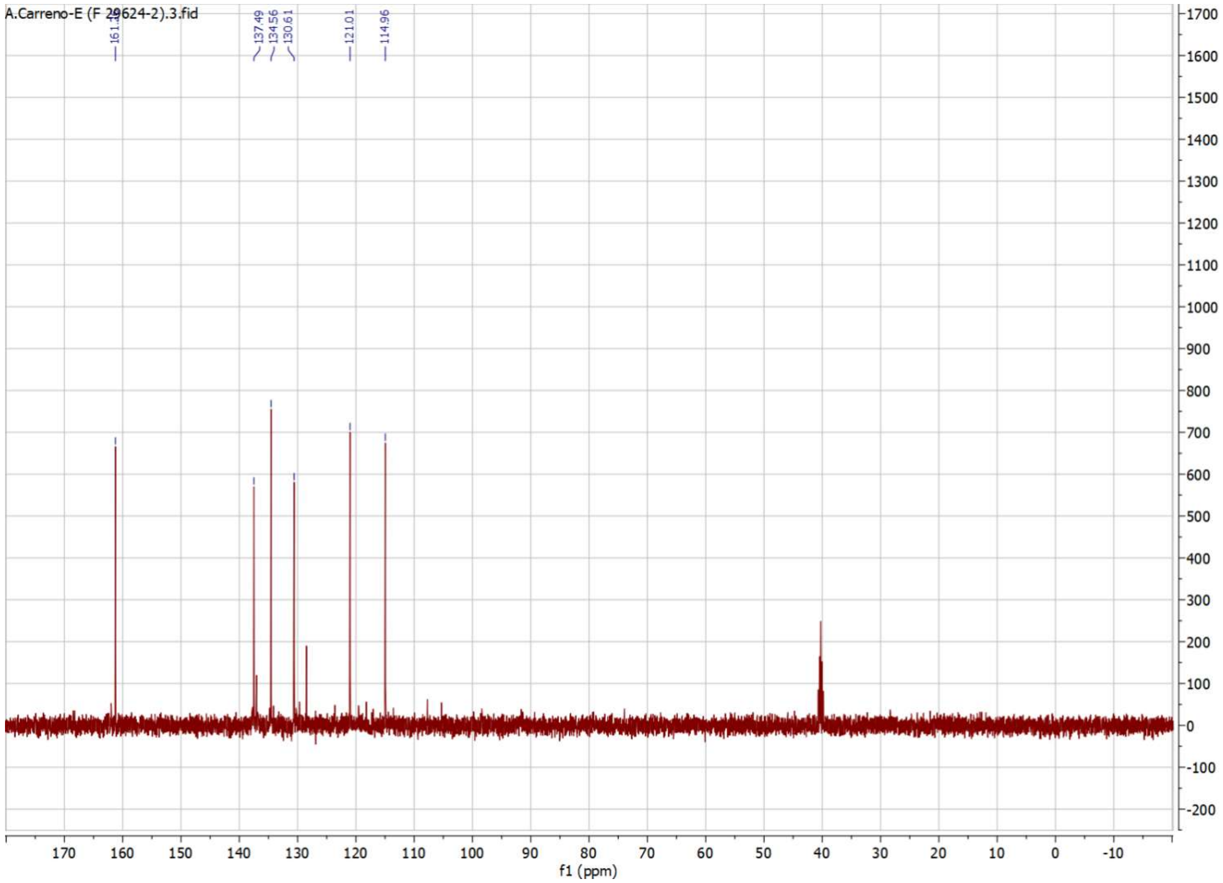

Figure S24. DEPT-45 of SB-4 in deuterated DMSO.

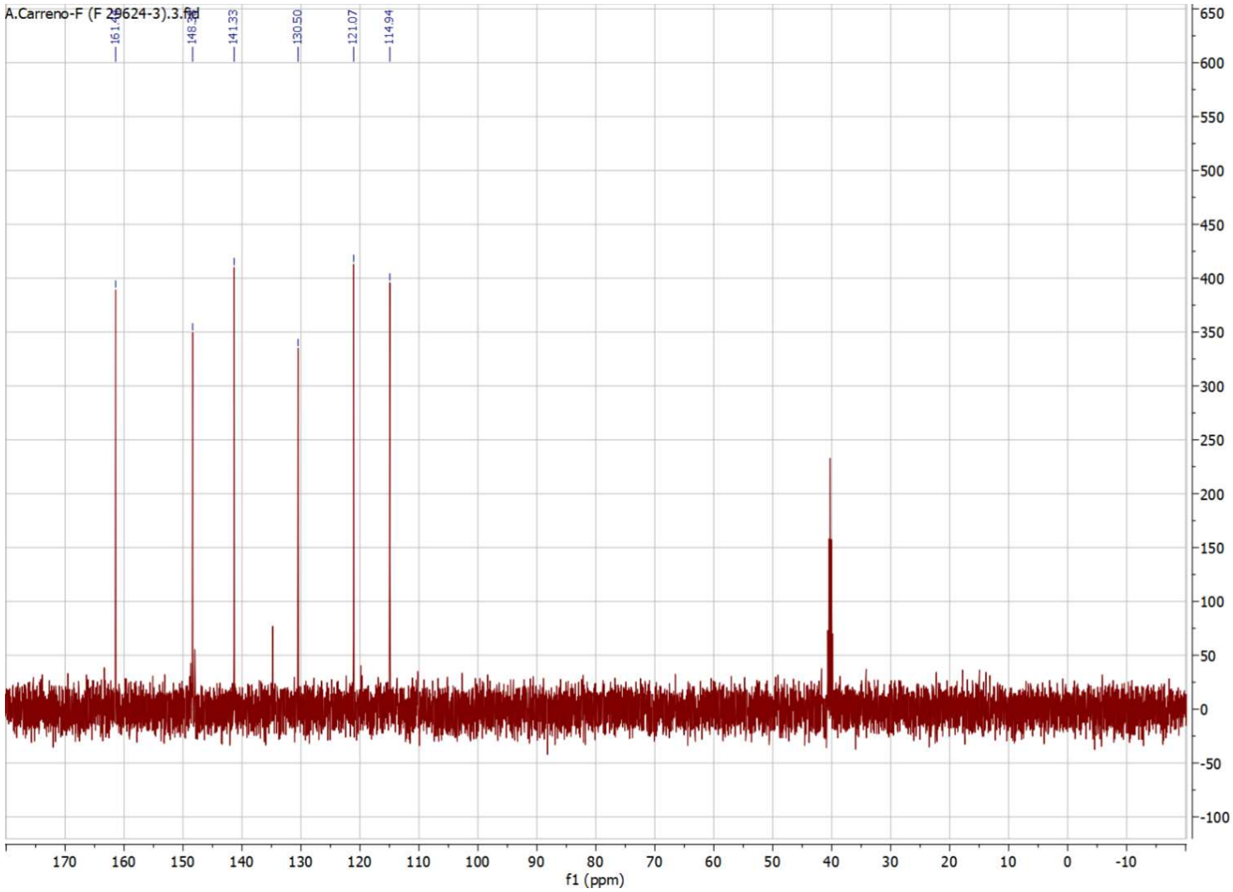

Figure S25. DEPT-45 of SB-5 in deuterated DMSO.

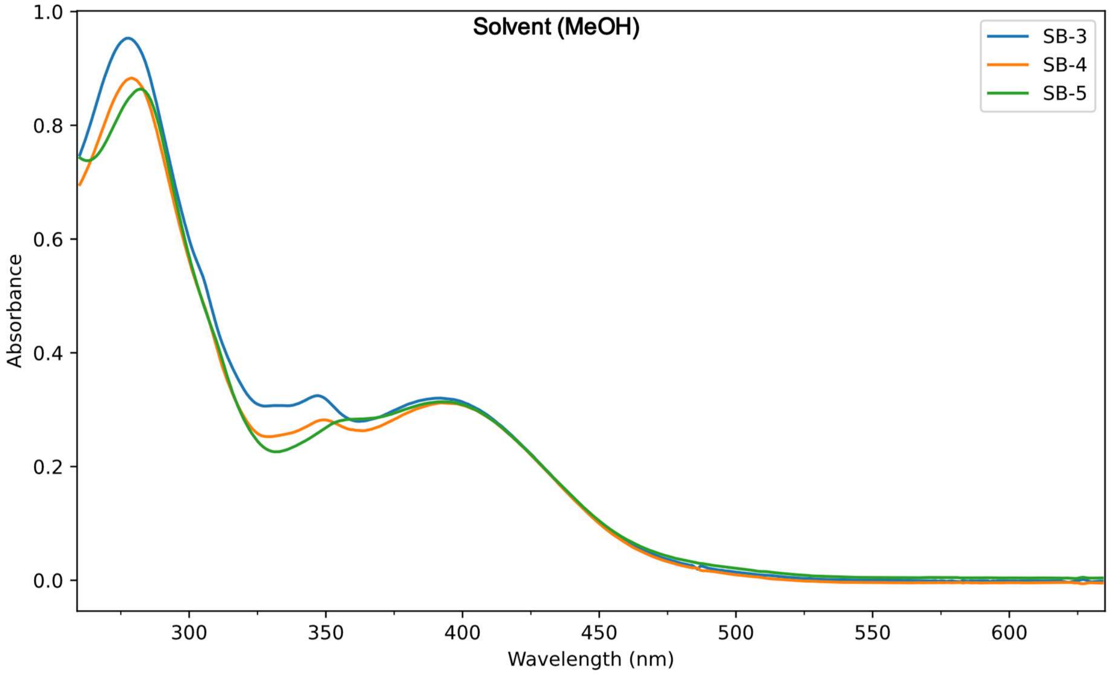

Figure S26. UV-Vis spectra of SB-3, SB-4, and SB-5 in aerated methanol (MeOH) at room temperature.

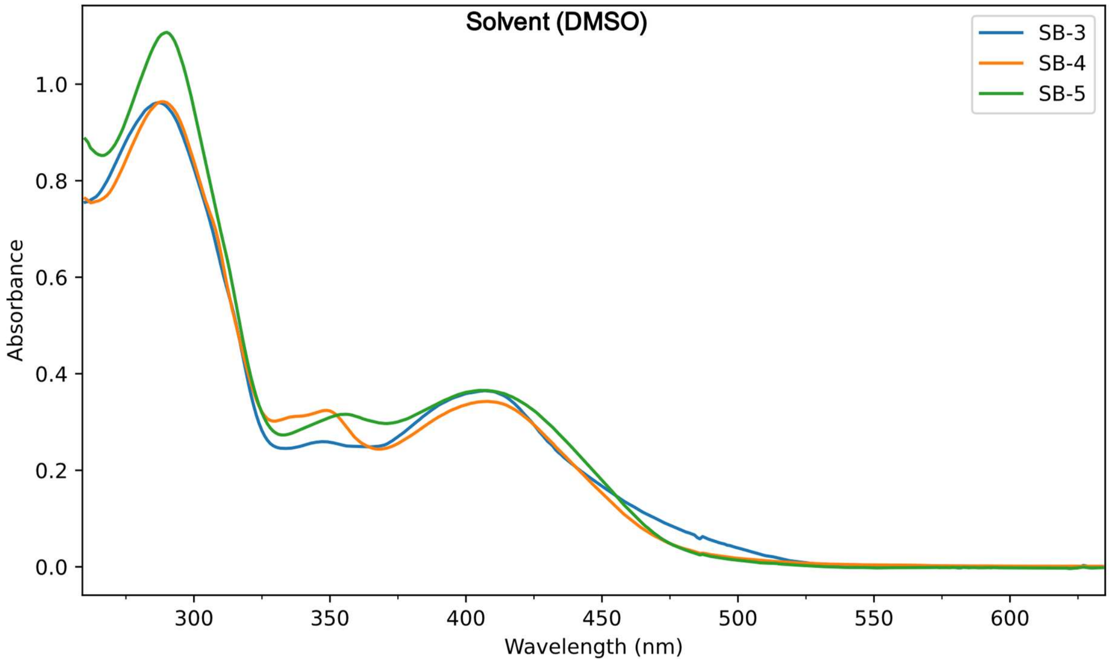

Figure S27. UV-Vis spectra of SB-3, SB-4, and SB-5 in aerated DMSO at room temperature.

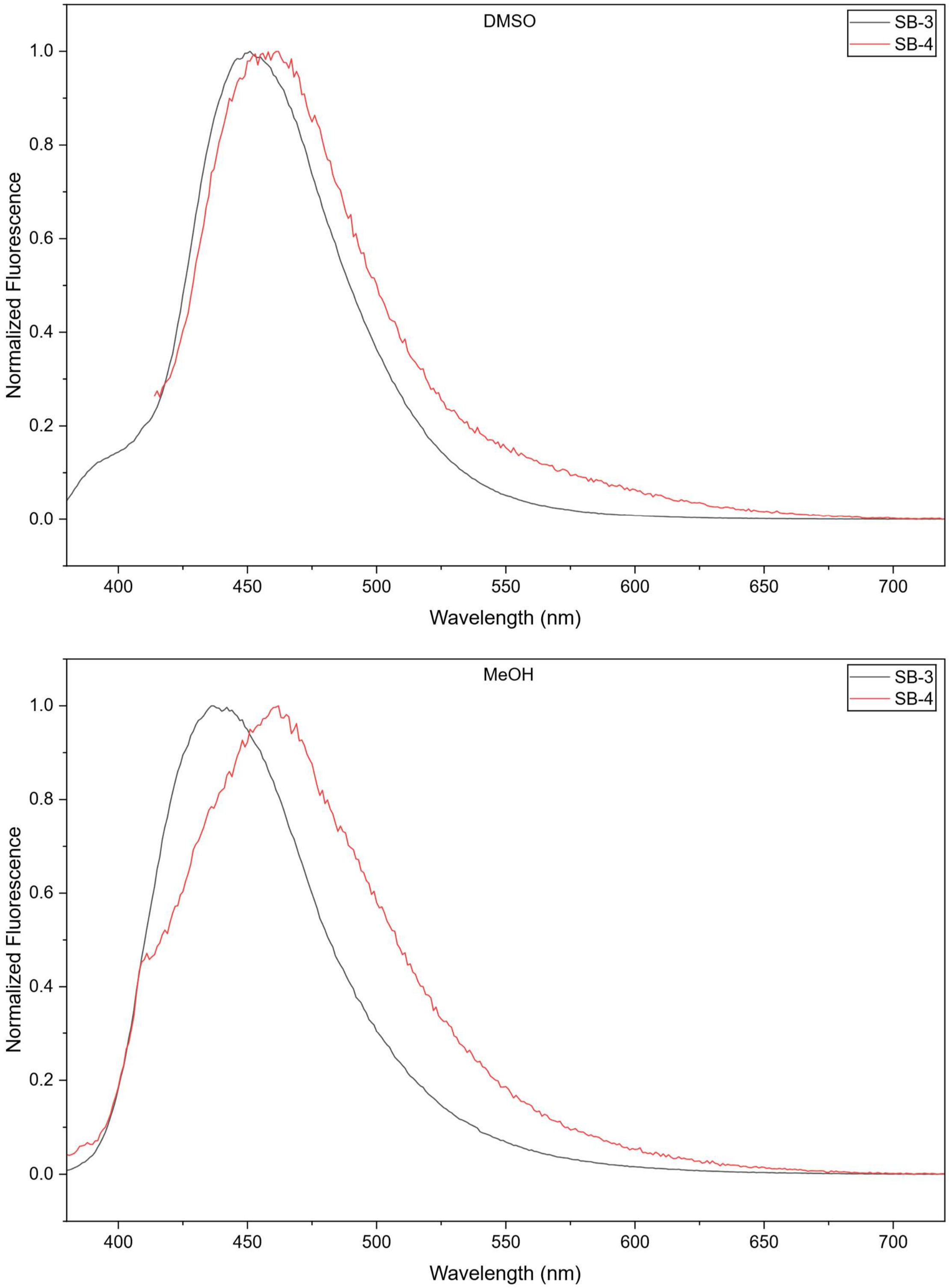

Figure S28. Normalized fluorescence of SB-3 and SB-4 in DMSO and MeOH solvents

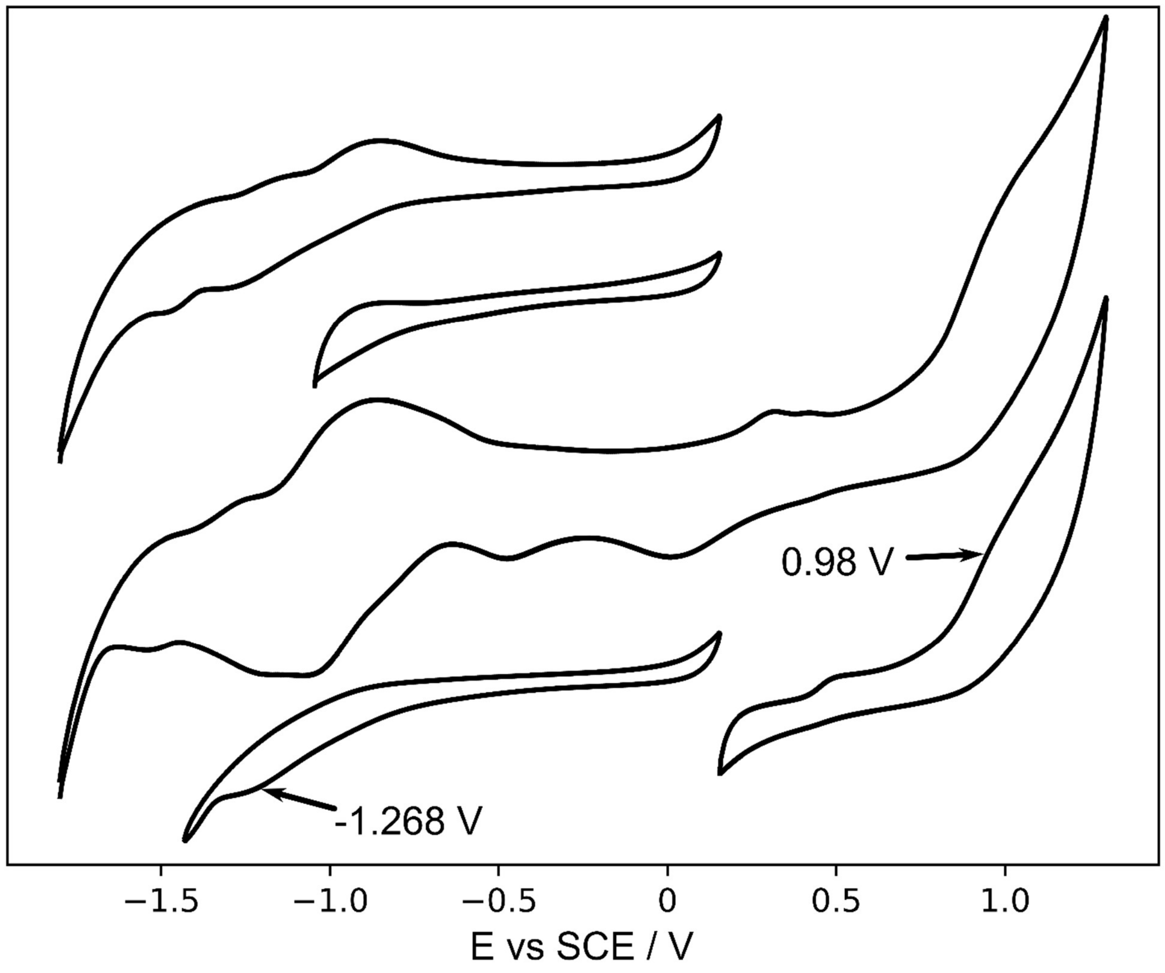

Figure S29. CV working-window study of SB-3. Interface: Pt | 1.0 x 10^{-5} M of analyte + 1.0 x 10^{-4} M $TBAPF_6$ in anhydrous ACN under an argon atmosphere.

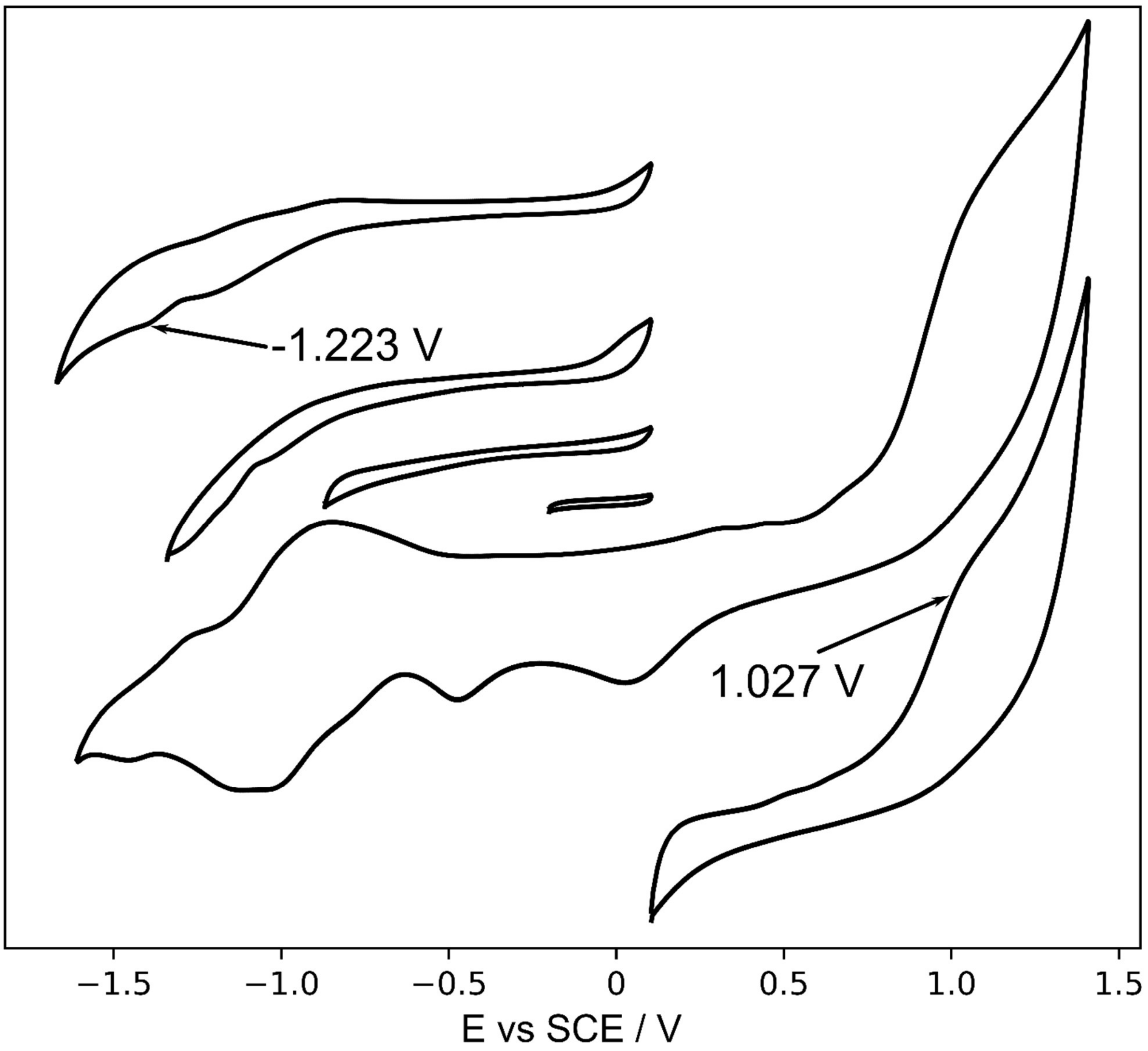

Figure S30. CV working-window study of SB-4. Interface: Pt ∣ 1.0 x 10^{-5} M of analyte + 1.0 x 10^{-4} M $TBAPF_6$ in anhydrous ACN under an argon atmosphere.

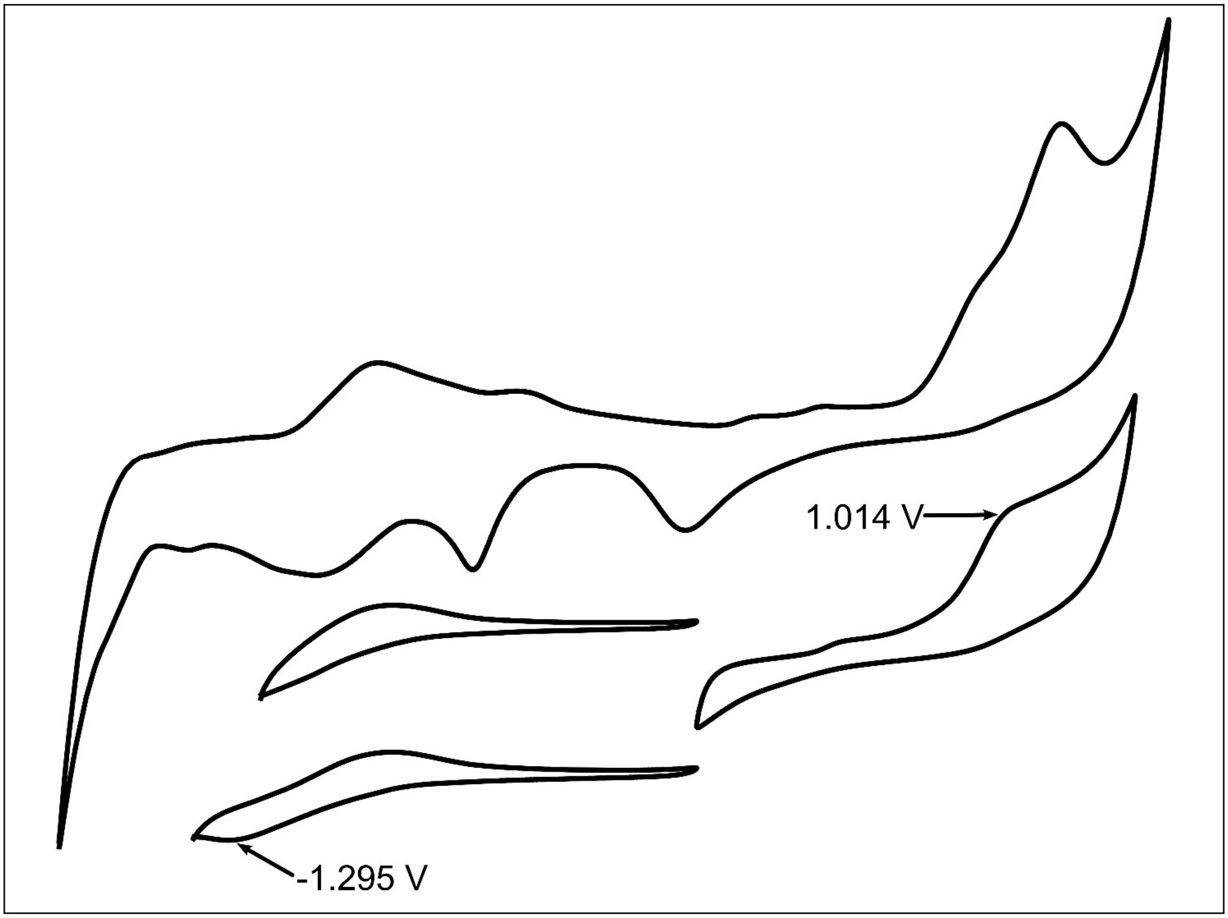

Figure S31. CV working-window study of SB-5. Interface: Pt | 1.0 x 10^{-5} M of analyte + 1.0 x 10^{-4} M $TBAPF_6$ in anhydrous ACN under an argon atmosphere.

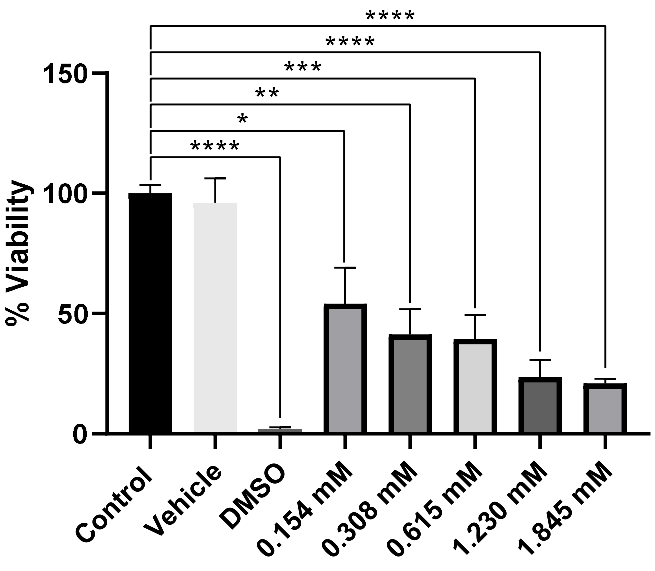

Figure S32. MTT viability of HeLa cells exposed to SB-3. HeLa cells were treated with SB-3 (dissolved in DMSO) for 24 h. Controls were: untreated (medium only, black bar, *control*), vehicle (DMSO alone at the same final volume fraction as in the highest SB-3 dose, light grey bar, *vehicle*), and a death control (5% v/v DMSO). Viability was normalized to the untreated control and reported as mean ± SEM (n = 3). Group differences versus control were evaluated by one-way ANOVA with Dunnett's post-hoc test. Significance codes: * $p < 0.05$; ** $p < 0.01$; *** $p < 0.001$; **** $p < 0.0001$ (two-sided; post-hoc–adjusted where applicable).

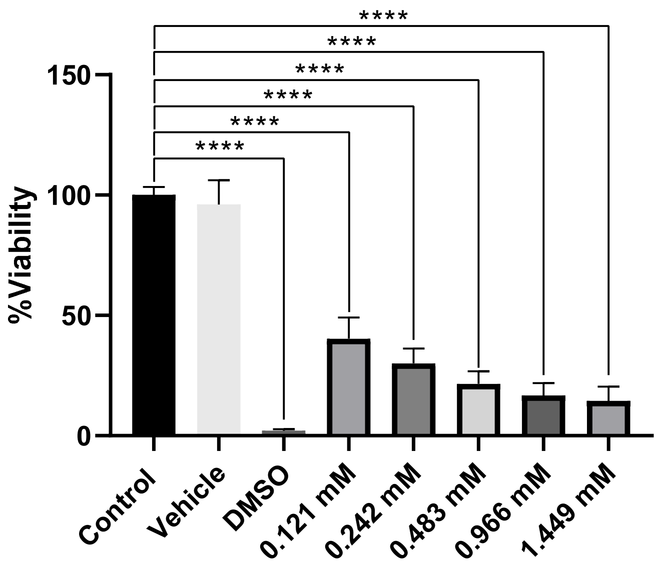

Figure S33. MTT viability of HeLa cells exposed to SB-4. HeLa cells were treated with SB-4 (dissolved in DMSO) for 24 h. Controls were: untreated (medium only, black bar, *control*), vehicle (DMSO alone at the same final volume fraction as in the highest SB-4 dose, light grey bar, *vehicle*), and a death control (5% v/v DMSO). Viability was normalized to the untreated control and reported as mean ± SEM (n = 3). Group differences versus control were evaluated by one-way ANOVA with Dunnett's post-hoc test. Significance codes: **** $p < 0.0001$ (two-sided; post-hoc–adjusted where applicable).

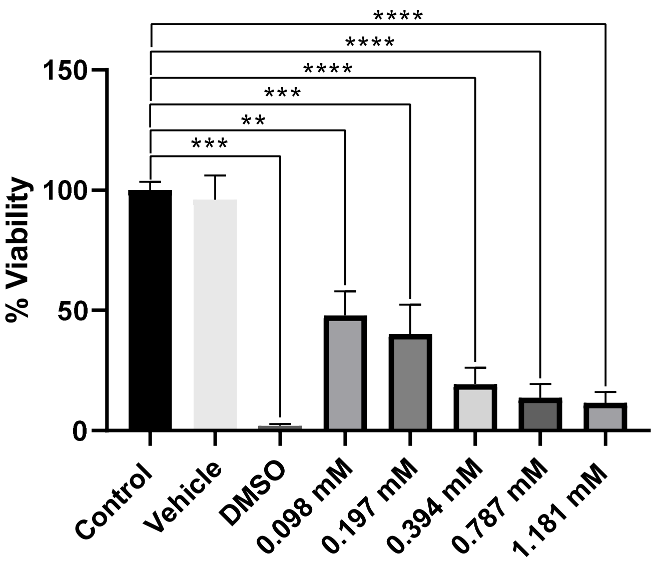

Figure S34. MTT viability of HeLa cells exposed to SB-5. HeLa cells were treated with SB-5 (dissolved in DMSO) for 24 h. Controls were: untreated (medium only, black bar, *control*), vehicle (DMSO alone at the same final volume fraction as in the highest SB-5 dose, light grey bar, *vehicle*), and a death control (5% v/v DMSO). Viability was normalized to the untreated control and reported as mean ± SEM (n = 3). Group differences versus control were evaluated by one-way ANOVA with Dunnett's post-hoc test. Significance codes: ** $p < 0.01$; *** $p < 0.001$; **** $p < 0.0001$ (two-sided; post-hoc–adjusted where applicable).

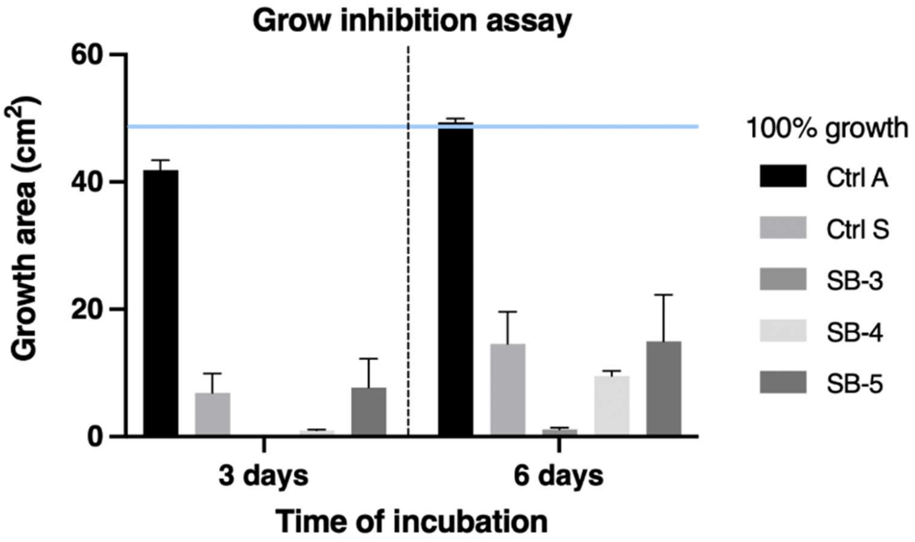

Figure S35. Mycelial growth of *Botrytis cinerea* B05.10 over 6 days in the presence of pyridine Schiff bases (SB-3, SB-4, and SB-5). Cultures were treated with each compound (dissolved in DMSO) or with DMSO alone (Ctrl S); an untreated control (Ctrl A) was also included. Growth area (expressed in cm^2) was measured on day 3 (left of the dotted line) and day 6 (right of the dotted line). The blue line represents 100% growth relative to the untreated control. The growth area was quantified using ImageJ (Fiji), and bars denote the mean ± SD from three biological replicates per treatment.
